# Atomic-level Ru-Ir mixing in rutile-type (RuIr)O_2_ for efficient and durable oxygen evolution catalysis

**DOI:** 10.1038/s41467-025-55910-1

**Published:** 2025-01-10

**Authors:** Yeji Park, Ho Yeon Jang, Tae Kyung Lee, Taekyung Kim, Doyeop Kim, Dongjin Kim, Hionsuck Baik, Jinwon Choi, Taehyun Kwon, Sung Jong Yoo, Seoin Back, Kwangyeol Lee

**Affiliations:** 1 Department of Chemistry and Research Institute for Natural Sciences, Korea University, Seoul, Republic of Korea; 2 Hydrogen Fuel Cell Research Center, Korea Institute of Science and Technology, Seoul, Republic of Korea; 3 Department of Chemical and Biomolecular Engineering, Institute of Emergent Materials, Sogang University, Seoul, Republic of Korea; 4 Department of Chemistry and Biological Engineering, Korea University, Seoul, Republic of Korea; 5 Korea Basic Science Institute (KBSI), Seoul, Republic of Korea; 6 Department of Chemistry, Incheon National University, Incheon, Republic of Korea; 7 Research Institute of Basic Sciences, Core Research Institute, Incheon National University, Incheon, Republic of Korea; 8 Division of Energy & Environment Technology, KIST school, University of Science and Technology (UST), Daejeon, Republic of Korea

**Keywords:** Hydrogen energy, Electrocatalysis, Electrocatalysis, Nanoparticles

## Abstract

The success of proton exchange membrane water electrolysis (PEMWE) depends on active and robust electrocatalysts to facilitate oxygen evolution reaction (OER). Heteroatom-doped-RuO_x_ has emerged as a promising electrocatalysts because heteroatoms suppress lattice oxygen participation in the OER, thereby preventing the destabilization of surface Ru and catalyst degradation. However, identifying suitable heteroatoms and achieving their atomic-scale coupling with Ru atoms are nontrivial tasks. Herein, to steer the reaction pathway away from the involvement of lattice oxygen, we integrate OER-active Ir atoms into the RuO_2_ matrix, which maximizes the synergy between stable Ru and active Ir centers, by leveraging the changeable growth behavior of Ru/Ir atoms on lattice parameter-modulated templates. In PEMWE, the resulting (RuIr)O_2_/C electrocatalysts demonstrate notable current density of 4.96 A cm^−2^ and mass activity of 19.84 A mg_Ru+Ir_^−1^ at 2.0 V. In situ spectroscopic analysis and computational calculations highlight the importance of the synergistic coexistence of Ru/Ir-dual-OER-active sites for mitigating Ru dissolution via the optimization of the binding energy with oxygen intermediates and stabilization of Ru sites.

## Abstract (web summary)

The development of durable catalysts for efficient hydrogen production from water faces challenges, particularly in the oxygen evolution reaction. Here, the authors report (RuIr)O_2_ electrocatalysts with enhanced performance, achieved by tuning the growth of Ru/Ir on lattice-modulated templates.

## Introduction

Renewable energy-powered proton exchange membrane water electrolysis (PEMWE) enables the cost-effective production of green hydrogen and thereby the establishment of a sustainable energy supply^1,2^. However, the large-scale deployment of PEMWE is hindered by the absence of efficient and durable electrocatalysts for the oxygen evolution reaction (OER)^3^. The commonly used IrO_2_ does not satisfactorily accelerate this reaction and is unstable in acidic environments^4–6^, whereas rutile-type RuO_2_ exhibits optimal affinity for OER intermediates (O*, OH*, and OOH*) and a high initial catalytic activity for OER but is more susceptible to metal-ion leaching than IrO_2_^7–9^.

Depending on the crystallinity of RuO_2_ and accessibility of its subsurface active sites, the RuO_2_-catalyzed OER can proceed through the lattice oxygen oxidation mechanism (LOM) or adsorbate evolution mechanism (AEM)^10,11^. The kinetically favorable LOM is intrinsically detrimental to catalyst stability, as the facile overoxidation of exposed surface Ru species affords soluble RuO_4_^2−^ ions, thus accelerating catalyst degradation and reducing OER performance^12,13^. Consequently, the stability of RuO_2_-based OER catalysts can be improved by suppressing the LOM and promoting the AEM.

The abovementioned mechanism steering can be achieved by doping RuO_2_ with foreign elements (e.g., Pt), which share their electrons with the neighboring active Ru sites^14–16^. However, a major drawback of this approach is the inactivity of Pt atoms as OER catalytic sites. To solve this problem, we herein strategically placed electron-donating Ir atoms near active Ru sites to improve the stability of RuO_2_-based OER catalysts and maximize the synergy between the two distinct OER active sites, namely Ru and Ir.

The Ir doping strategy relied on the formation of a well-mixed RuIr alloy, which, in turn, required the synchronous decomposition of Ru and Ir precursors. In typical solution-phase colloidal syntheses, the Ru precursor is reduced faster than the Ir precursor, which prevents the uniform distribution of Ru and Ir in the resulting catalysts. Given that the lattice mismatch between the template and growing metallic phase during template-mediated synthesis can affect the metal deposition rate^17–20^, we hypothesized that the use of large-lattice-mismatch templates may decelerate the initial deposition of Ru and thus favor the formation of mixed RuIr alloy phases. To prove this idea, we prepared templates with different surface lattice parameters, namely pristine and lattice-expanded Ni_3_S_4_ nanorods, demonstrating that the abated deposition and attachment of Ru on the latter template resulted in the formation of a well-mixed RuIr phase.

The thermal oxidation of the above RuIr phase on lattice-expanded Ni_3_S_4_ (e-Ni_3_S_4_) afforded a well-mixed rutile-type (RuIr)O_2_/C electrocatalyst with high OER performance. This catalyst showed a low overpotential of 174 mV at 10 mA cm^−2^ and maintained its initial activity over 360 h of operation at a high current density of 100 mA cm^−2^. When used as the anode catalyst layer of a PEMWE, (RuIr)O_2_/C achieved a high current density of >4.96 A cm^−2^ and mass activity of 19.84 A mg_Ru+Ir_^−1^ at 2.0 V, with minimal degradation observed over 250 h operation at 1.0 A cm^−2^. In situ X-ray absorption spectroscopy (XAS) and in situ differential electrochemical mass spectrometry (DEMS) analyses, combined with density functional theory (DFT) calculations, revealed that the incorporation of Ir atoms not only stabilized the local coordination environment around Ru, fostering the AEM pathway, but also facilitated Ir-to-Ru electron transfer via bridging oxygens, thereby hindering Ru dissolution during the OER. Furthermore, the coexistence of Ru and Ir at the cation sites of the rutile-type oxide phase at the atomic scale led to optimal oxygen-adsorbate binding energies and outstanding OER activity. Overall, the synergistic effect resulting from the atomic-level mixing of Ru and Ir effectively enhanced the activity and stability of the Ru-based OER catalyst under acidic conditions, providing valuable insights for the rational design of practical electrocatalysts. This study pave the way for the industrial-scale production of green hydrogen and thus contribute to establishing a sustainable society.

## Results

### Preparation of mixed rutile-type (RuIr)O_2_/C

**Fig. 1:**
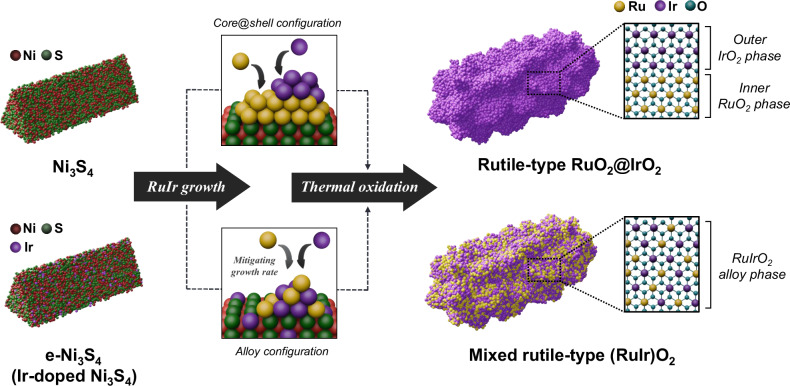
Synthesis process of Ru/Ir oxide-based nanostructures. Schematic illustration of Ru/Ir oxide-based nanostructures with different Ru/Ir atomic configurations. Red, green, yellow, purple, and cyan balls represent Ni, S, Ru, Ir, and O atoms, respectively.

**Fig. 2:**
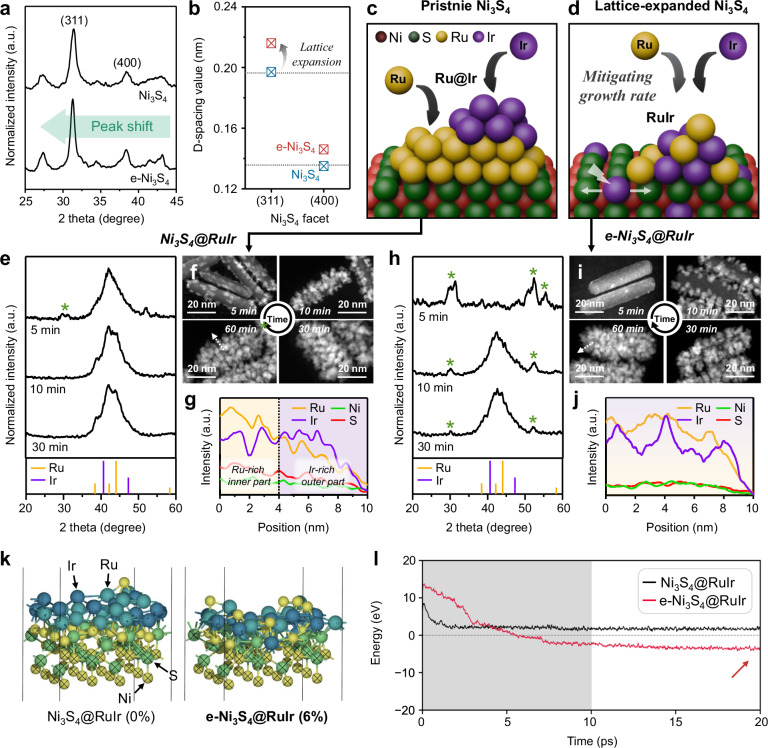
Growth mechanism of Ru/Ir on two distinct templates. **a** PXRD patterns of Ni_3_S_4_ and e- Ni_3_S_4_. **b** Comparison of the d-spacing values for the (311) and (400) facets of the Ni_3_S_4_ phase, observed in PXRD patterns in Fig. 2a. The two dashed lines represent the reference d-spacing values for the (311) and (400) facets. Schematic illustration of Ru and Ir growth on **c** Ni_3_S_4_ and **d** e-Ni_3_S_4_ templates, which afforded Ru@Ir and RuIr shell configurations, respectively. Green and red spheres denote Ni and S atoms. **e**, **h** PXRD patterns and **f**, **i** HAADF-STEM images (scale bar = 20 nm) of Ni_3_S_4_@RuIr and e- Ni_3_S_4_@RuIr obtained at reaction times of 5, 10, and 30 min. Color bars and asterisks in PXRD patterns indicate the reference peaks of *hcp* Ru (yellow, #01-088-2333), *fcc* Ir (purple, #06-0598), and Ni_3_S_4_ (green, #01-076-1813). Line profile analysis of **g** Ni_3_S_4_@RuIr and **j** e-Ni_3_S_4_@RuIr determined along the lines marked by arrows in **f** and **i**, respectively. **k** Final images of the AIMD trajectories of Ni_3_S_4_@RuIr and e-Ni_3_S_4_@RuIr. Light green, yellow, turquoise, and navy spheres denote Ni, S, Ru, and Ir, respectively. **l** Energy profiles for the AIMD trajectories in **k**, with the gray region indicating the 10 ps equilibrium process.

On the premise that the lattice parameter of the template surface is an important factor in the control of the atomic mixing between Ru and Ir atoms of the RuIr alloy phase (Fig. 1), we prepared the two types of templates: pristine Ni_3_S_4_ (Supplementary Fig. 1) and lattice-expanded Ni_3_S_4_ (e-Ni_3_S_4_) nanorods (Supplementary Fig. 2). The latter lattice-expanded template was prepared by introducing larger-sized Ir atoms (with a radius of 112 pm for Ir, compared to 110 pm for Ni) into the Ni_3_S_4_ framework. Notably, the powder X-ray diffraction (PXRD) pattern of e-Ni_3_S_4_ (Fig. 2a and Supplementary Fig. 2e) demonstrated a slight shift to a lower angle compared to Ni_3_S_4_, indicating the lattice expansion due to the embedding of Ir single atoms in the Ni_3_S_4_ matrix. The d-spacing analysis of Ni_3_S_4_ facets, derived from high-angle annular dark-field scanning transmission electron microscopy (HAADF-STEM) images and their corresponding fast Fourier transform (FFT) patterns (Fig. 2b and Supplementary Fig. 2d, f) revealed approximately 6% lattice expansion in the Ni_3_S_4_ phase of e-Ni_3_S_4_, providing compelling evidence of the structural modification achieved through Ir doping. Moreover, the X-ray photoelectron spectroscopy (XPS) analysis for Ni 2p and S 2p showed that the Ir dopant does not cause any changes in the electronic structure of the template but only alters the lattice structure (Supplementary Fig. 3 and Supplementary Tables 1, 2).

As anticipated, the Ru/Ir growth behavior depended on the lattice parameter of the template (Fig. 2c–j, Supplementary Fig. 4 and Supplementary Note 1). For pristine Ni_3_S_4_, rapid Ru deposition followed by Ir deposition on the Ni_3_S_4_ was observed during the early stages of the reaction. This stepwise growth resulted in the formation of a Ru@Ir inner shell@outer shell configuration (Ni_3_S_4_@RuIr; Fig. 2g and Supplementary Fig. 5) However, when e-Ni_3_S_4_ was reacted simultaneously with Ru^3+^ and Ir^3+^, the initial rate of Ru deposition decreased owing to a significant lattice mismatch between the growing Ru phase and e-Ni_3_S_4_ surface. Consequently, the atomic-level mixing of Ru and Ir was promoted, leading to the formation of a RuIr alloy structure (e-Ni_3_S_4_@RuIr; Fig. 2j and Supplementary Fig. 6).

To understand the correlation between the lattice size of the templates and the growth behavior of Ru/Ir atoms on them, we determined the average interatomic bond lengths (*d*_ave_) in bulk and shell structures using DFT calculations (Supplementary Fig. 7, Supplementary Note 2, and Supplementary Data 1). When Ru atoms were grown on the templates, the difference in *d*_ave_ between Ru bulk and e-Ni_3_S_4_@Ru (0.233 Å) was notably larger than that between bulk Ru and Ni_3_S_4_@Ru (0.098 Å), implying a lattice mismatch between the Ru atoms and e-Ni_3_S_4_ surface. In addition, the formation of Ru–Ru clusters upon the growth of Ru atoms on the e-Ni_3_S_4_ surface contributed to the decreased *d*_ave_ of e-Ni_3_S_4_@Ru (Supplementary Fig. 8). Conversely, the difference in *d*_ave_ between RuIr bulk and e-Ni_3_S_4_@Ru (0.121 Å) was similar to that between RuIr bulk and Ni_3_S_4_@Ru (0.144 Å), which suggested that the stability of the RuIr shell was maintained on the expanded core. Further Ab initio molecular dynamics (AIMD) simulations using a Ni_3_S_4_ core–metal shell interface model were conducted to rationalize the stabilization of the RuIr phase on the e-Ni_3_S_4_ surface (Fig. 2k, l). The RuIr shell structure was stabilized in the e-Ni_3_S_4_ model, which indicated that lattice expansion of the Ni_3_S_4_ phase promoted the simultaneous deposition of Ru and Ir atoms. Thus, our results suggest that template lattice modulation likely plays a significant role in influencing the Ru/Ir growth behavior.

**Fig. 3:**
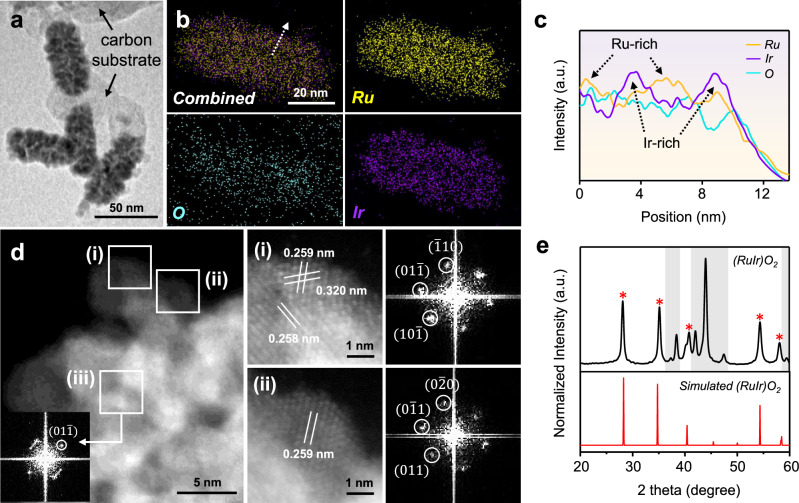
Characterization of (RuIr)O_2_/C electrocatalysts. **a** TEM image of (RuIr)O_2_/C prepared by the thermal oxidation of the RuIr phase on e-Ni_3_S_4_. **b** Combined and individual EDS elemental mapping images of Ru (yellow), Ir (purple), and O (cyan). The white arrow indicates the range of the line profile in **c**. **c** Line profile analysis of (RuIr)O_2_/C corresponding to the marked area indicated by the white arrow in **b**. **d** Normal and enlarged HAADF-STEM images of (RuIr)O_2_/C with corresponding FFT patterns. White boxes in **d** (i)–(ii) and (iii) indicate oxide and metallic species, respectively. **e** PXRD patterns of (RuIr)O_2_/C. Gray boxes denote the remaining metallic species in (RuIr)O_2_/C.

The e-Ni_3_S_4_@RuIr and Ni_3_S_4_@RuIr were loaded onto carbon support (Vulcan XC-72R) (Supplementary Fig. 9) to prevent nanoparticle aggregation, and the resulting composites (e-Ni_3_S_4_@RuIr/C and Ni_3_S_4_@RuIr/C, respectively) were annealed at 400 °C in 40% O_2_ balanced with N_2_ for 2 h. The transmission electron microscopy (TEM) image (Fig. 3a) of thermally oxidized e-Ni_3_S_4_@RuIr/C ((RuIr)O_2_/C) revealed patchy Ru/Ir branches with dendritic morphology on the e-Ni_3_S_4_ surface. The elemental mappings (Fig. 3b) and line profiles (Fig. 3c) obtained using energy-dispersive X-ray spectroscopy (EDS) exhibited a relatively uniform distribution of Ru and Ir in the alloy form within (RuIr)O_2_/C, in line with the atomic distribution observed in e-Ni_3_S_4_@RuIr/C prior to thermal oxidation. The lattice spacings of 0.259, 0.258, and 0.320 nm, corresponding to rutile-type oxide ($01\bar{1}$), ($10\bar{1}$), and ($\bar{1}10$) facets, respectively, were observed in the outer regions of (RuIr)O_2_/C by HAADF-STEM image and corresponding FFT patterns (parts i and ii of Fig. 3d and Supplementary Fig. 10b). In contrast, the inner region of (RuIr)O_2_/C showed the ($01\bar{1}$) (part iii of Fig. 3d) and ($004$) (Supplementary Fig. 10c) facets indicative of a residual metallic phase. The PXRD pattern of (RuIr)O_2_/C (Fig. 3e) closely matched those of simulated (RuIr)O_2_ (red) and residual metallic (gray box) structures, consistent with the HAADF-STEM (Fig. 3d) and high-resolution TEM (HRTEM) results (Supplementary Fig. 10). In contrast, the thermal oxidation of Ni_3_S_4_@RuIr/C resulted in different Ru/Ir configurations (RuO_2_@IrO_2_/C; Supplementary Figs. 11, 12). RuO_2_@IrO_2_/C exhibited a gradient atomic distribution, with the inner and outer regions predominantly composed of Ru- and Ir-rich oxides, respectively. The local electronic structures of (RuIr)O_2_/C and RuO_2_@IrO_2_/C were further investigated using XPS analysis (Supplementary Figs. 13, 14, and Supplementary Tables 3, 4). Both (RuIr)O_2_/C and RuO_2_@IrO_2_/C were primarily composed of metallic species before thermal oxidation (Supplementary Fig. 13). Thermal oxidation resulted in the oxidation of the Ru phase, whereas the Ir phase maintained its metallic character and was only minimally oxidized in both (RuIr)O_2_/C and RuO_2_@IrO_2_/C (Supplementary Fig. 14). In all cases, the Ni_3_S_4_-based templates were partially oxidized to NiO and partially retained as Ni_3_S_4_ during the thermal treatment (Supplementary Fig. 15 and Supplementary Note 3).

In summary, by regulating the lattice parameters of the Ni_3_S_4_ template surface, we prepared two types of Ru/Ir oxides with different atomic configurations while preserving the initial Ru/Ir atomic arrangements.

### Electrocatalytic OER performances of (RuIr)O_2_/C electrocatalyst

The electrocatalytic OER performances of (RuIr)O_2_/C and RuO_2_@IrO_2_/C were evaluated in N_2_-saturated 0.1 M HClO_4_ and compared with that of home-made RuO_2_ nanoparticles/C (RuO_2_ NPs/C) (Supplementary Fig. 16), commercial RuO_2_/C (com. RuO_2_/C), and commercial IrO_2_/C (com. IrO_2_/C). The cyclic voltammetry (CV) curves exhibited discernible reversible peaks corresponding to changes in the oxidation states of Ru and Ir within (RuIr)O_2_/C and RuO_2_@IrO_2_/C. As shown in Supplementary Fig. 17a, two distinct redox peaks for Ru^3+^/Ru^4+^ (0.7 V_RHE_) and Ir^4+^/Ir^5+^ (1.2$-$1.5 V_RHE_) were observed in (RuIr)O_2_/C. However, in the case of RuO_2_@IrO_2_/C (Supplementary Fig. 17b), a redox peak corresponding to Ru^4+^/Ru^6+^ was observed around 0.9 V_RHE_, which is not seen in (RuIr)O_2_/C^21,22^. These results imply that the Ir in (RuIr)O_2_/C undergoes over-oxidation instead of Ru, with rutile Ru^4+^ remaining stable, whereas, in RuO_2_@IrO_2_/C, overoxidized Ru is present but unstable.

**Fig. 4:**
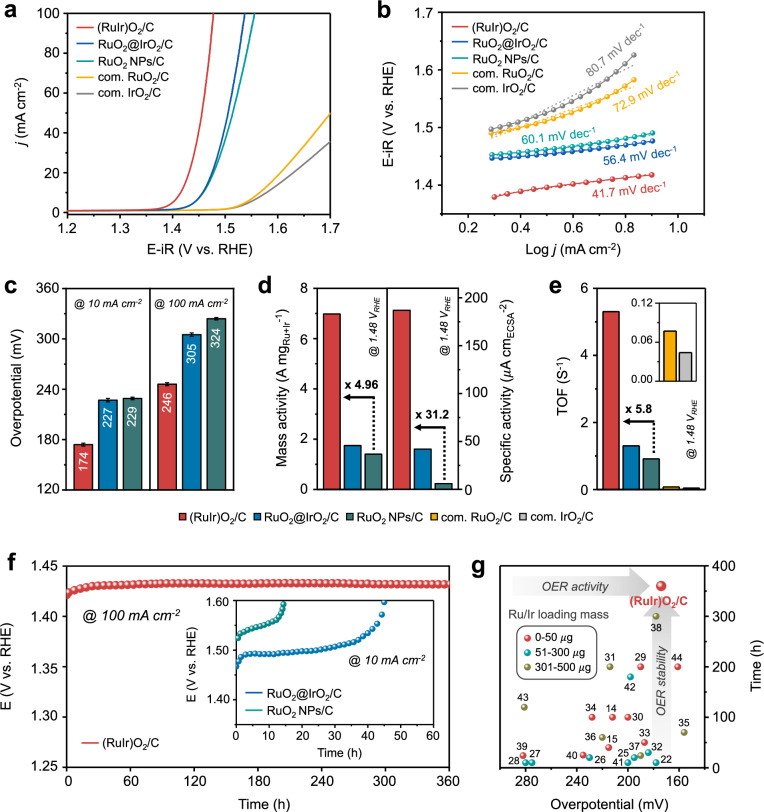
Electrochemical OER performance. **a** LSV curves of (RuIr)O_2_/C, RuO_2_@IrO_2_/C, RuO_2_ NPs/C, com. RuO_2_/C, and com. IrO_2_/C in 0.1 M HClO_4_ (pH = 1.02) at a scan rate of 5 mV s^−1^ and 1600 rpm. The noble metal loading was 50 µg_Ru+Ir_ cm^−2^ for each electrocatalysts. The measured potentials were 100% iR-compensated using the determined R_S_ value of 12 $\pm$ 0.3 Ω. **b** Tafel plots constructed based on the curves in Fig. 4a with fitted lines for Tafel slope. **c** Overpotentials required to achieve current densities of 10 mA cm^−2^ and 100 mA cm^−2^, **d** mass and specific activities, and **e** TOF values at 1.48 V_RHE_. **f** Chronopotentiometry test of (RuIr)O_2_/C at 100 mA cm^−2^ in 0.1 M HClO_4_ (inset: chronopotentiometry test of RuO_2_@IrO_2_/C and RuO_2_ NPs/C at 10 mA cm^−2^). **g** Comparison of catalytic performances of recently reported representative Ru/Ir-based electrocatalysts with different noble metal loadings for the OER in acidic electrolytes.

The linear sweep voltammetry (LSV) curves indicated that compared to the control-group catalysts, (RuIr)O_2_/C required a lower overpotential of 174 $\pm$ 1.8 mV and 246 $\pm$ 1.2 mV to achieve a current density of 10 mA cm^−2^ and 100 mA cm^−2^, respectively (Fig. 4a, c). LSV curves without iR-compensation were presented in Supplementary Fig. 18. Besides, the Tafel slope of (RuIr)O_2_/C (41.7 mV dec^−1^) was notably smaller than those of RuO_2_@IrO_2_/C (56.4 mV dec^−1^) and RuO_2_ NPs/C (60.1 mV dec^−1^) (Fig. 4b). These results suggested that (RuIr)O_2_/C exhibited notable catalytic activity and fast kinetics, indicating that the thorough mixing of Ru and Ir atoms considerably enhanced the OER performance. The results of electrochemical impedance spectroscopy (EIS) analysis at 1.40 V_RHE_ (Supplementary Fig. 19) revealed that (RuIr)O_2_/C showed low charge-transfer resistance and rapid electron transfer from the electrode to the catalyst surface during the OER, which agreed with the results of Tafel slope analysis.

To investigate the intrinsic activities of the Ru/Ir oxide–based catalysts, we compared their mass activities (MAs) and specific activities (SAs) at an overpotential of 250 mV (1.48 V_RHE_). The active metal (Ru and Ir) loadings of the electrodes were determined by inductively coupled plasma-atomic emission spectroscopy (ICP-AES) (Supplementary Table 5). The MA of (RuIr)O_2_/C (6.98 A mg_Ru+Ir_^−1^) exceeded those of RuO_2_@IrO_2_/C (1.75 A mg_Ru+Ir_^−1^) and RuO_2_ NPs/C (1.41 A mg_Ru_^−1^) 3.98- and 4.96-fold, respectively (Fig. 4d, left). The SAs were calculated by normalizing the OER polarization curves with respect to the electrochemically active surface area (ECSA), which, in turn, was determined from the dependence of double-layer capacitance (*C*_dl_) on different scan rate (Supplementary Fig. 20)^8,23,24^. The SA of (RuIr)O_2_/C (187 A cm_ECSA_^−2^) exceeded those of other samples, indicative of its comparable intrinsic activity (Fig. 4d, right and Supplementary Fig. 21). Furthermore, we calculated the turnover frequency (TOF) to demonstrate the efficiency of electrocatalysts for oxygen evolution. The TOF of (RuIr)O_2_/C (5.30 s^−1^) surpassed those of RuO_2_@IrO_2_/C (1.31 s^−1^), RuO_2_ NPs/C (0.92 s^−1^), com. RuO_2_/C (0.08 s^−1^), and com. IrO_2_/C (0.04 s^−1^) (Fig. 4e).

Additionally, we synthesized unsupported (RuIr)O_2_ and RuO_2_@IrO_2_ electrocatalysts using SiO_2_ as a sacrificial substrate instead of carbon. This approach eliminates the potential issue of carbon corrosion during the OER operation and allows for the evaluation of Ru/Ir atomic configurations and their impact on OER performance (Supplementary Fig. 22). As shown in Supplementary Fig. 23 and Supplementary Note 4, the unsupported catalysts retained the same atomic configuration as those synthesized on carbon supports. Although the overall performance of unsupported (RuIr)O_2_ showed a slight decrease compared to its carbon-supported counterpart ((RuIr)O_2_/C), it still exhibited enhanced OER activity compared to unsupported RuO_2_@IrO_2_ (Supplementary Fig 24). While the carbon support enhances electrochemical performance by preventing nanoparticle aggregation and improving electrical conductivity, the Ru-Ir atomic interaction within the mixed rutile-type oxide phase remains the primary factor driving the high OER performance.

The stability of (RuIr)O_2_/C during the OER operation was probed by chronopotentiometry (CP) measurements at a constant current density (Fig. 4f). (RuIr)O_2_/C demonstrated outstanding stability, showing only a slight increase in overpotential after 360 h at 100 mA cm^−2^, whereas both RuO_2_@IrO_2_/C and RuO_2_ NPs/C lost their initial activity within 40 and 15 h, respectively, at 10 mA cm^−2^. These results underscored the inherent robustness of (RuIr)O_2_/C during the long-term electrochemical OER in harsh acidic environments. Notably, the OER performance of (RuIr)O_2_/C exceeded those of recently reported Ru- and Ir-based OER catalysts (Fig. 4g and Supplementary Table 6)^14,15,22,25–44^. To uncover the structural changes occurring during the OER, we characterized the structures of the catalysts after OER operation. The results of TEM and EDS elemental mapping analyses (Supplementary Figs. 25–27) showed that the dendritic shell thickness of (RuIr)O_2_/C remained unchanged, and no noticeable detachment of the Ru/Ir shell was observed, which suggested that the structural integrity of (RuIr)O_2_/C was preserved. In contrast, the dendritic shell of RuO_2_@IrO_2_/C thinned due to the dendrite detachment. In addition, HRTEM image and corresponding FFT patterns (Supplementary Fig. 28) indicated that the crystallinity of (RuIr)O_2_/C was preserved, whereas that of RuO_2_@IrO_2_/C deteriorated because of Ru and Ir leaching. The results of inductively coupled plasma-mass spectrometry (ICP-MS) analysis confirmed negligible leaching of Ru and Ir from (RuIr)O_2_/C and considerable loss of Ru and Ir from RuO_2_@IrO_2_/C and RuO_2_ NPs/C during long-term OER operation (Supplementary Fig. 29).

The valence electronic structures of (RuIr)O_2_/C and RuO_2_@IrO_2_/C after the OER operation were examined by XPS. Compared with those of RuO_2_@IrO_2_/C, the Ru 3p_3/2_ (Supplementary Fig. 30) and Ir 4 f (Supplementary Fig. 31) peaks of (RuIr)O_2_/C were shifted toward lower and higher binding energies, respectively. This shift indicated a higher electron density at the Ru sites of (RuIr)_2_/C and implied facilitated electron transfer from Ir to Ru. Thus, in (RuIr)O_2_/C, Ru was protected from dissolution during the OER, and the rutile-type Ru^4+^ species were largely preserved, whereas RuO_2_@IrO_2_/C contained a considerable amount of overoxidized Ru in the form of Ru^6+^ (RuO_4_^2−^) (Supplementary Fig. 30c, d). Additionally, Ir in (RuIr)O_2_/C appeared as Ir^5+^, which suggested that Ir was overoxidized in preference to Ru (Supplementary Fig. 31c). The notable increase in the amorphous Ir^3+^ content of RuO_2_@IrO_2_/C suggested the oxidation of Ir in the shell to porous IrO_*x*_ during the OER (Supplementary Fig. 31d). This oxidation exposed the RuO_2_ core, leading to Ru overoxidation and thereby causing Ru dissolution. The O 1 s XPS spectra (Supplementary Fig. 32 and Supplementary Table 7) revealed the changes in the contribution of lattice oxygen (O_M-O_), hydroxyl groups (O_M-OH_), and adsorbed water (O_M-H2O_) in both (RuIr)O_2_/C and RuO_2_@IrO_2_/C after OER. For (RuIr)O_2_/C, the lattice oxygen was well retained, which emphasized the robustness of the surface oxygen species and high stability of (RuIr)O_2_/C, whereas for RuO_2_@IrO_2_/C, the lattice oxygen content decreased. Hence, the intermixed Ir atoms in the RuO_2_ phase emerged as a crucial factor for stabilizing the RuO_2_ matrix during the long-term OER.

In summary, the Ir atoms positioned at neighboring cation sites alongside the Ru atoms underwent sacrificial oxidation during the long-term OER, thereby safeguarding the Ru atoms against excessive oxidation and thus increasing catalyst stability.

### Performance of (RuIr)O_2_/C in single-cell PEMWE

**Fig. 5:**
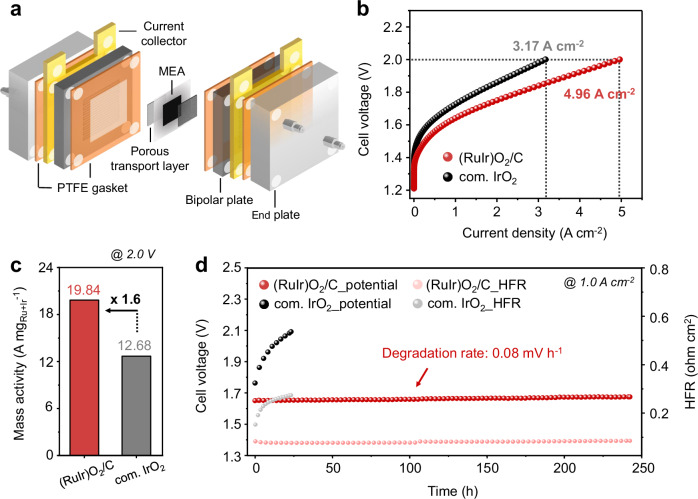
Single-cell performances in PEMWE. **a** Schematic illustration of the membrane-electrode assembly used for PEMWE. **b** Polarization curves for PEMWE using (RuIr)O_2_/C and com. IrO_2_ as anode catalysts and commercial Pt/C as cathode catalyst in 0.1 M HClO_4_ at 80 °C. The noble metal loadings were 0.25 mg_Ru+Ir_ cm^−2^ for the anode and 1 mg_Pt_ cm^−2^ for the cathode. Current densities at a cell voltage of 2.0 V are 4.96 A cm^−2^ and 3.17 A cm^−2^ for (RuIr)O_2_/C and com. IrO_2_, respectively. No cell volatges were iR compensated. **c** Mass activities (A mg_Ru+Ir_^−1^) of (RuIr)O2/C and com. IrO_2_ at a cell voltage of 2.0 V. **d** OER stability test of PEMWE cells with (RuIr)O_2_/C and com. IrO_2_ recorded at 1.0 A cm^−2^. The HFR on the right y-axis means high-frequency resistance.

To further assess the feasibility of using (RuIr)O_2_/C in practical PEMWE devices, we examined its single-cell performance using membrane electrode assemblies (MEAs) (Fig. 5a and Supplementary Fig. 33). The initial measurements focused on determining the catalyst loading per unit area, which directly influences single-cell OER performance. Interestingly, the catalytic activity of (RuIr)O_2_ deteriorated with increasing catalyst loading, indicating that the MEA thickness considerably affected the charge transport efficiency (Supplementary Fig. 34a)^45,46^. This observation was further supported by EIS analysis (Supplementary Fig. 34b, c), which revealed that both ohmic and charge-transfer resistances increased with thicker MEAs, underscoring the need to optimize catalyst loading for balancing activity and efficient charge transfer within the MEA^47^. Consequently, the examination of changes in the catalytic activity of (RuIr)O_2_/C showed that charge transfer was facilitated even at a low catalyst loading (0.25 mg cm^−2^), which resulted in high catalytic activity.

Next, we compared the activity of commercial IrO_2_ (com. IrO_2_) in a system using the same single-cell material. At a cell voltage of 2.0 V, (RuIr)O_2_/C achieved a high current density of 4.96 A cm^−2^, which was 56% higher than that observed for com. IrO_2_ (3.17 A cm^−2^) (Fig. 5b) and exhibited a notable MA of 19.84 A mg_Ru+Ir_^−1^ at a loading of only 0.25 mg_Ru+Ir_ cm^−2^ (Fig. 5c). The single-cell performance of (RuIr)O_2_/C demonstrates enhanced efficiency, achieving high activity even at low catalyst loading, highlighting its intrinsic capability for the OER. Furthermore, the EIS results obtained for (RuIr)O_2_/C and com. IrO_2_/C in the high-voltage region (2.0 V), where the mass-transfer effects were most pronounced, revealed enhanced membrane contact and rapid charge transfer on the surface of (RuIr)O_2_/C (Supplementary Fig. 35). The long-term assessment of the PEMWE performance using MEAs with (RuIr)O_2_/C and com. IrO_2_ at a constant current density of 1.0 A cm^−2^ (Fig. 5d) showed that the (RuIr)O_2_/C maintained a stable cell voltage over 250 h with a very low deterioration rate of 0.08 mV h^−1^. Additionally, SEM analysis was conducted to assess the surface morphology and cross-section of the MEAs before and after the single-cell durability test, specifically examining catalyst detachment and dissolution behavior during PEWWE operation. Notably, the commercial IrO_2_ (with a reduction rate of 40.37%) exhibited a significant decrease in catalyst layer thickness, whereas the (RuIr)O_2_/C showed only a modest reduction of about 28.66% during the durability test (Supplementary Figs. 36, 37). This demonstrates that the (RuIr)O_2_/C remained robust with minimal degradation, effectively preserving its OER performance over prolonged PEMWE operation.

### Origin of the improved OER activity and stability of (RuIr)O_2_/C

**Fig. 6:**
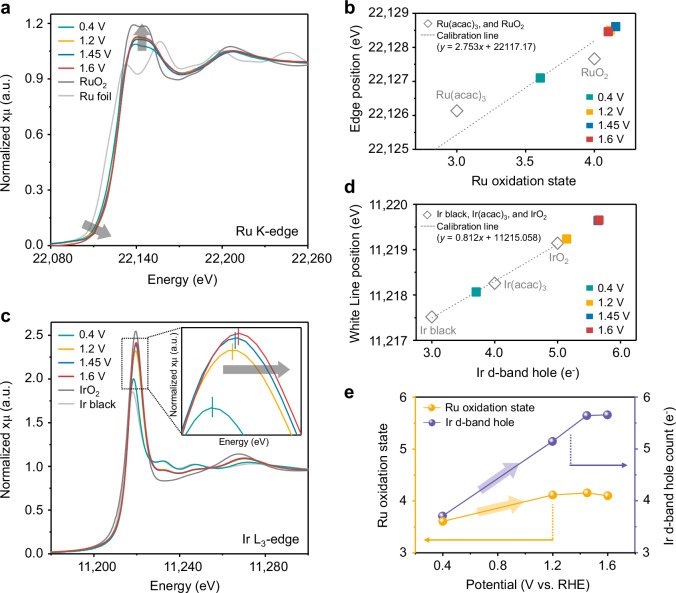
In-situ XANES analysis. **a** Ru K-edge XANES spectra of (RuIr)O_2_/C at different applied potentials in O_2_-saturated 0.1 M HClO_4_. **b** Change in the Ru K-edge position of (RuIr)O_2_/C as a function of the Ru valence state. **c** Ir L_3_-edge XANES spectra of (RuIr)O_2_/C recorded at different applied potentials in O_2_-saturated 0.1 M HClO_4_. **d** Change in the Ir L_3_-edge white line position of (RuIr)O_2_/C as a function of the formal d-band hole count. **e** Change in the Ru oxidation state (yellow, left) and Ir formal d-band hole count (purple, right) upon an increase in applied potential from 0.4 to 1.6 V_RHE_.

To elucidate the origin of the improved OER activity and stability of (RuIr)O_2_/C, we examined changes in the chemical states of Ru and Ir during the OER using in situ Ru K-edge and Ir L_3_-edge X-ray absorption fine structure (XAFS) spectroscopy at applied potentials of 0.4–1.6 V_RHE_ (Supplementary Fig. 38). The Ru K-edge X-ray absorption near-edge structure (XANES) spectra (Fig. 6a) showed a minimal positive shift in the pre-edge position with increasing potential. The Ru K-edge position, determined from the first derivatives of the XANES spectra (Supplementary Fig. 39), was plotted as a function of the Ru oxidation state (Fig. 6b). Ru foil (Ru^0^), Ru(acac)_3_ (Ru^3+^; acac = acetylacetonate), and RuO_2_ (Ru^4+^) powders were used as reference materials, and a slope of 2.753 eV per oxidation-state unit was obtained. The Ru oxidation state increased from 3.69 at +0.4 V_RHE_ to 4.11 at +1.2 V_RHE_, while the change in the valence state at higher potentials was negligible. Therefore, the Ru^4+^ states in (RuIr)O_2_/C remained securely intact at a high overpotential (+1.6 V_RHE_), effectively preventing the overoxidation of Ru species during the OER.

The changes in the Ir oxidation state were very different from those in the Ru oxidation state. The Ir L_3_-edge XANES spectrum of (RuIr)O_2_/C (Fig. 6c) showed broad white lines (WLs) corresponding to the 2p-to-5d transition^48,49^. In addition, Ir L_3_-edge XANES analysis revealed a pronounced positive shift in the WL position and intensity upon a potential increase from 0.4 to 1.6 V_RHE_. The quantitative change in the Ir d-band states was analyzed using d-band hole counts derived from the WL positions, which were determined from the second derivatives of the XANES spectra (Supplementary Fig. 40)^49–51^. The Ir L_3_-edge WL position as a function of the formal d-band hole count was depicted in Fig. 6d. A slope of 0.812 eV per d-band hole was obtained for Ir black (5d^7^6s^2^), Ir(acac)_3_ (5d^6^6s^0^), and IrO_2_ (5d^5^6s^0^)^50–52^. A notable depopulation of Ir d-band states with increasing potential was observed, with Ir d-band hole counts of 3.18, 5.14, 5.64, and 5.66 *e*^–^ observed at +0.4, +1.2, +1.45, and +1.6 V_RHE_, respectively. In summary, (RuIr)O_2_/C showed a considerable depopulation of Ir d-band states with increasing potential (Δ*d* = 2.48 for 0.4–1.6 V_RHE_), whereas the Ru oxidation state was maintained at approximately +4 in the 1.2–1.6 V_RHE_ range (Fig. 6e). Therefore, Ir with a substantial number of d-band holes was concluded to boost OER activity while suppressing the detrimental overoxidation of Ru within the mixed rutile-type phase.

**Fig. 7:**
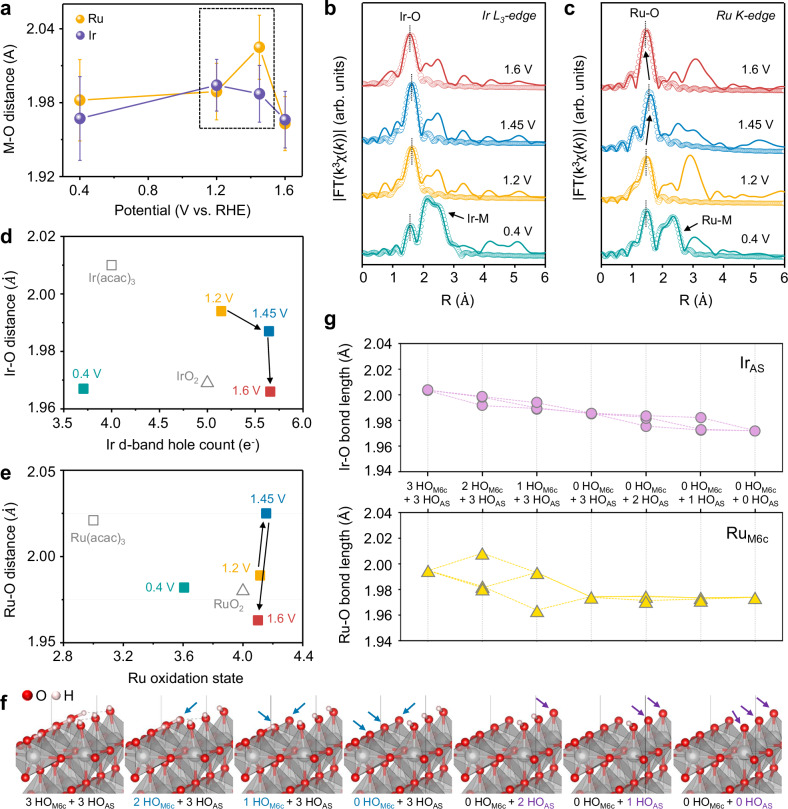
In-situ EXAFS analysis and DFT calculations for M–O bonding. **a** Ru–O (red) and Ir–O (blue) bond lengths in (RuIr)O_2_/C obtained by the analysis of in situ FT-EXAFS spectra. In situ **b** Ir L_3_-edge and **c** Ru-K edge FT-EXAFS spectra of (RuIr)O_2_/C obtained at different applied potentials in O_2_-saturated 0.1 M HClO_4_. Experimental operando **d** Ir–O and **e** Ru–O distances of (RuIr)O_2_/C as functions of Ir d-band hole count and Ru oxidation state, respectively, under applied potentials of +0.4, +1.2, +1.45, and +1.6 V_RHE_. **f** Atomic configurations with different coverages calculated for H-covered rutile (110) surface structures (HO_M6c_ and HO_AS_ are H atoms bonded to O atoms on six-coordinated metal sites (M_6c_) and active sites (AS), respectively.) **g** DFT-calculated M–O bond lengths (Boltzmann-averaged) for all (RuIr)O_2_ surface structures according to surface coverage. Ir_AS_ and Ru_M6c_ correspond to Ir at AS and Ru at M_6c_, respectively.

Alterations in the local coordination structures around the Ru and Ir atoms in (RuIr)O_2_/C were further investigated using in situ extended X-ray absorption fine structure (EXAFS) spectroscopy. The Ru K-edge and Ir L_3_-edge Fourier-transformed (FT) EXAFS spectra were subjected to least-squares fitting, with the thus derived structural parameters summarized in Supplementary Tables 8, 9. Fig. 7a presents Ru–O and Ir–O bond lengths as functions of the applied potential. The Ir–O bond length of (RuIr)O_2_/C decreased from 1.994 to 1.966 Å upon a potential increase from +1.2 to +1.6 V_RHE_ (Fig. 7b), which indicated the considerable oxidation of Ir during the OER not attributable to hole-doped states induced by surrounding vacancy formation (Fig. 7d)^51^. Conversely, Ru–O bond length exhibited a different behavior, being considerably larger at +1.45 V_RHE_ (2.025 Å) than at +1.2 V_RHE_ (1.989 Å) and +1.6 V_RHE_ (1.963 Å) (Fig. 7c). This Ru–O bond elongation during the on-site catalytic stage (from +1.2 V_RHE_ to +1.45 V_RHE_) could weaken the electron-withdrawing effect of the neighboring coordinated oxygen atoms and thus decrease the Ru oxidation state to prevent catalyst dissolution^53^. Furthermore, by examining the M–O distance as a function of the Ir d-band hole count (Fig. 7d) and Ru oxidation state (Fig. 7e), we confirmed that the (RuIr)O_2_/C-catalyzed OER proceeded via the AEM pathway, i.e., did not involve lattice oxygen, which resulted in high catalyst stability.

To validate the experimentally determined alteration in the M–O bond length with changes in the OER potential, we analyzed the variation in the Ru–O and Ir–O bond lengths on the catalyst surface during deprotonation using DFT calculations. Previous studies have highlighted the surface transition from OH* termination to O* termination in rutile-type structures under an applied oxidation potential^54^. In this context, we constructed surface structures with different numbers of adsorbed H* species and examined the lengths of surface metal–oxygen bonds (Fig. 7f, g). Interestingly, the Ir–O bond length decreased upon surface deprotonation, whereas certain Ru–O bond lengths concomitantly increased. This behavior suggested that the Ru–O bond elongation in (RuIr)O_2_ at +1.45 V_RHE_, as observed by in situ EXAFS spectroscopy, was due to the elongation of the Ru–O bond adjacent to the deprotonated O_2c_ (Supplementary Fig. 41). Additionally, as deprotonation progressed up to +1.6 V_RHE_, contraction of the Ru–O bond was observed. Theoretical analysis suggested that this is due to the deprotonation of HO_2c_ bonded to Ru at oxidative potentials, resulting in the formation of O_2c_. To further explore the effect of metal type on M–O bond length changes, we analyzed the M–O bond lengths of pure rutile-type oxides (IrO_2_, RuO_2_) and (Ru_AS_Ir_M6c_)O_2_, where Ir and Ru were located at M_6c_ and AS, respectively (Supplementary Fig. 42). Upon surface deprotonation, the X_AS_–O (X = Ir, Ru) bond length decreased in all cases, whereas some X_M6c_–O bond lengths increased. The above findings suggest that the positioning of Ru at M_6c_ within the rutile-type (RuIr)O_2_ increased the Ru–O bond length, which was consistent with in situ EXAFS results.

### Enhanced OER mechanism of (RuIr)O_2_/C via AEM pathway

**Fig. 8:**
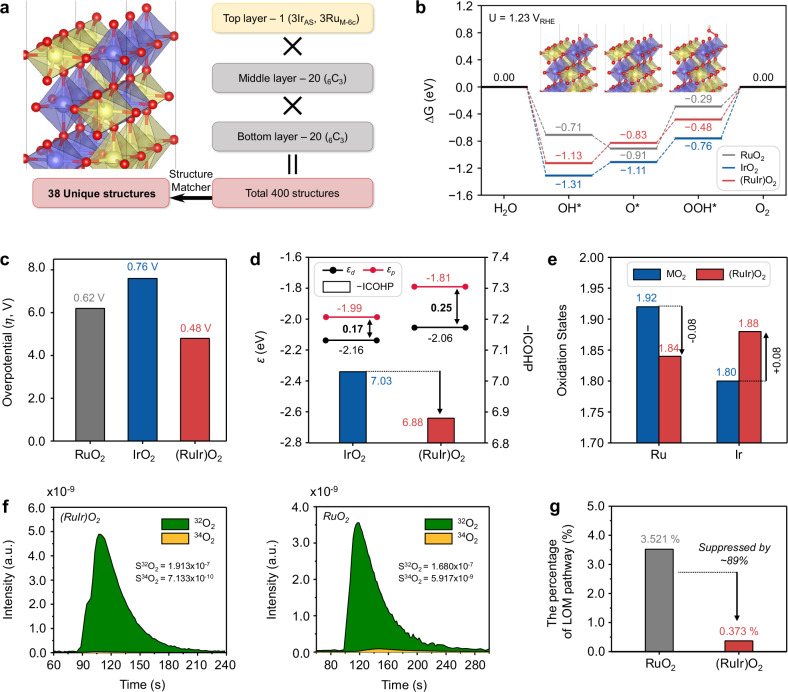
DFT calculations and DEMS analysis for OER mechanism. **a** Schematic illustration of (RuIr)O_2_ structure generation. Red, yellow, and blue spheres correspond to O, Ru, and Ir atoms, respectively. **b** Gibbs free energy diagram for the adsorption of OER intermediates on RuO_2_, IrO_2_, and (RuIr)O_2_ at 1.23 V_RHE_. **c** Theoretical OER overpotentials of RuO_2_, IrO_2_, and (RuIr)O_2_. **d** *d*-band center (*ε*_*d*_) and *p-*band center (*ε*_*p*_) positions for surface Ir and adsorbed O*, respectively, and Boltzmann-averaged −ICOHP values of Ir–O* bonding for IrO_2_ and (RuIr)O_2_. Smaller −ICOHP values indicate weaker M–O bonding. **e** Average oxidation states of surface atoms for MO_2_ (M = Ru, Ir) and (RuIr)O_2_. **f** DEMS signals of ^34^O_2_ (^16^O^18^O) and ^32^O_2_ (^16^O^16^O) from the evolved O_2_ for the ^18^O-labeled (RuIr)O_2_/C (left) and homemade-RuO_2_/C (right) in 0.1 M HClO_4_ solution in H_2_^16^O. **g** Percentage contribution of lattice oxygen (LOM%) in the OER.

To theoretically rationalize the notable OER performance of the (RuIr)O_2_ catalyst compared with those of RuO_2_ and IrO_2_, we generated (110) surfaces for RuO_2_, IrO_2_, and (RuIr)O_2_ using their bulk lattice parameters (Supplementary Note 5). Given the considerable thickness of the outer shell of RuO_2_@IrO_2_, its computational modeling was similar to that of IrO_2_. The surface structure of (RuIr)O_2_ was constructed by considering the oxidation state of Ir and its position within the surface structure (Supplementary Figs. 43–46 and Supplementary Note 6). Various Ir positions, including active sites (AS), six-coordinated metal sites (M_6c_), subpositions of active sites (sub-AS), and subpositions of six-coordinated metal sites (sub-M_6c_), were examined. Based on the results of in situ XANES analysis (Fig. 6), all Ir atoms were placed at the AS, where they exhibited the highest oxidation states. The cations were then arranged to achieve a Ru/Ir atomic ratio of 1:1 in each layer. In this manner, we created all possible (RuIr)O_2_ surface structures (total: _6_C_3_ × _6_C_3_ = 400), from which 38 unique surface structures were extracted (Fig. 8a and Supplementary Figs. 47, 48). Utilizing these surface structures, we calculated the Gibbs free energy changes of OER intermediates for all sites across 38 structures and 3 sites (114 calculations in total), following the AEM. The corresponding weighted averages were then determined using the Boltzmann probability, with more stable surface structures contributing more significantly to material properties (Supplementary Fig. 49). During the AEM pathway (Fig. 8b, c), the Ir active sites in (RuIr)O_2_ exhibited weaker affinities for all oxygen intermediates (OH*, O*, OOH*) compared to those in IrO_2_. Consequently, (RuIr)O_2_ demonstrated a higher OER activity (overpotential (*η)* = 0.48 V) than pure rutile-type oxides (*η* = 0.76 V for IrO_2_, *η* = 0.62 V for RuO_2_). Although (RuIr)O_2_ exhibited better OER activity than RuO_2_ and IrO_2_ through the LOM pathway (Supplementary Fig. 50 and Supplementary Note 7), the calculated overpotentials were significantly higher compared to those of the AEM pathway. Therefore, (RuIr)O_2_ exhibits notable OER activity compared to RuO_2_ and IrO_2_, particularly highlighting the efficiency of the OER process via the AEM pathway.

The difference in energy levels ($|{\varepsilon }_{d}-{\varepsilon }_{p}|$) between the *d*-band center (*ε*_*d*_) of the active-site metal and *p*-band center (*ε*_*p*_) of adsorbed O* reflects the M–O interaction strength^55^. Specifically, chemical binding properties are determined by electron transfer between the metal d-orbital and adsorbed O* p-orbital, with smaller differences between ${\varepsilon }_{d}$ and ${\varepsilon }_{p}$ indicating stronger M–O bonding^56^. In this regard, we observed an increase in $|{\varepsilon }_{d}-{\varepsilon }_{p}|$ from 0.17 (IrO_2_) to 0.25 ((RuIr)O_2_), which suggested weakened M–O bonding between the O* adsorbate and Ir active sites in (RuIr)O_2_ (Fig. 8d and Supplementary Fig. 51). Additionally, the – integrated crystal orbital Hamilton population (–ICOHP) for M–O*, which indicates bond strength, was lower for (RuIr)O_2_ than for IrO_2_ (Fig. 8d and Supplementary Fig. 52). These results imply that the presence of both Ru and Ir at the cation sites of the rutile-type oxide weakened metal–adsorbate bonding, thereby increasing the OER activity of (RuIr)O_2_. Furthermore, we examined the oxidation states of the surface cations in the rutile-type oxides (Fig. 8e). Bader charge analysis revealed a decrease (increase) of 0.08 in the oxidation state of Ru (Ir) in the mixed rutile-type oxide, indicating electron transfer from Ir to Ru. This finding suggests that overoxidation inhibited Ru dissolution and thus increased catalyst stability under harsh OER conditions^9,23,57^. Overall, (RuIr)O_2_/C demonstrated enhanced OER performance attributed to the synergistic effect of increased Ir activity and Ru stability.

We further carried out in-situ differential electrochemical mass spectrometry (DEMS) analyses using the isotope ^18^O to investigate to verify the suppressed lattice oxygen participation on (RuIr)O_2_ catalysts during the OER^58,59^. Before DEMS measurement, the ^18^O-labeled (RuIr)O_2_ and home-made RuO_2_ catalysts were prepared by CV cycling in a 0.1 M HClO_4_ in heavy-oxygen water (H_2_^18^O). Then, the evolved O_2_ was measured by DEMS in a 0.1 M HClO_4_ electrolyte of H_2_^16^O (Supplementary Fig. 53). The signals of the ^34^O_2_ indicate the direct ^16^O-^18^O coupling, where the ^16^O originates from water and ^18^O originates from the lattice oxygen^60^. The participation ratio of lattice oxygen (LOM%) was evaluated by the ratio of ^34^O_2_ to (^32^O_2_ + ^34^O_2_). As shown in Fig. 8f, g, the LOM% of the (RuIr)O_2_ was only 0.373%, whereas the LOM% of the homemade-RuO_2_ (3.521%) was about ~9.4-fold higher than that of (RuIr)O_2_. Therefore, the lattice oxygen participation during the OER was significantly hindered in the (RuIr)O_2_, which corroborates with its high OER stability over RuO_2_ under acidic conditions.

## Discussion

In summary, efficient rutile-type (RuIr)O_2_/C electrocatalysts with notable intrinsic activity and stability at 100 mA cm^−2^ of current density were developed through the changeable growth behavior of Ru/Ir atoms on lattice-parameter-modulated templates. The results of in situ XAS analysis and DFT calculations demonstrated that the improved catalytic performance arises from the maximized synergy between the Ru and Ir atoms mixed at the atomic level. The Ir atoms stabilized the local coordination environment around Ru, promoting the AEM and adjusting the valence electronic structure of the Ru sites to prevent Ru overoxidation and achieve a durable OER during prolonged operation. Moreover, the atomic-scale coexistence of Ru and Ir resulted in optimized oxygen-intermediate adsorption energy and outstanding catalytic activity. We expect that the modulation of the surface parameters of template can be leveraged to form various alloy materials and develop new electrocatalytic materials.

## Methods

### Chemicals

NiCl_2_·H_2_O (99.95% trace metal basis), Ir(acac)_3_ (97%), Ru(acac)_3_ (97%), 1,2-hexadecanediol (technical grade, 90%), 1-dodecanethiol (${{{\boldsymbol{\ge }}}}$98%), oleic acid (technical grade, 90%), oleylamine (OAm, technical grade, 70%), and 1-octadecene (technical grade, 90%) were purchased from Sigma-Aldrich. All chemicals were used as received without further purification.

### Synthesis of pristine Ni_3_S_4_ and e-Ni_3_S_4_ templates

The pristine Ni_3_S_4_ template was synthesized using a previously reported method with minor modifications^61^. A slurry of NiCl_2_·H_2_O (0.2 mmol), 1,2-hexadecanediol (0.4 mmol), oleic acid (0.2 mL), 1-octadecene (5 mL), and OAm (1.2 mL) was prepared in a 100 mL Schlenk tube equipped with a magnetic stirrer. The tube was placed in an oil bath held at 90 °C, evacuated for 60 min, and charged with Ar (1 atm). After the injection of 1-dodecanethiol (0.5 mL), the tube was placed in a preheated oil bath held at 120 °C and maintained at this temperature for 40 min. Finally, the oil bath was heated to 225 °C for more than 15 min. The reaction mixture was cooled to room temperature, and the dark precipitate was washed with isopropanol/acetone (15 mL/15 mL) and collected via centrifugation. The washing process was repeated twice. The precipitate was dispersed in toluene for the further growth of Ru and Ir. To synthesize the e- Ni_3_S_4_ template, the sample was further heated from 225 to 240 °C. When the oil bath temperature reached 240 °C, a stock solution of Ir(acac)_3_ (0.025 mmol) in OAm (3 mL) was injected into the Schlenk tube, and the reaction mixture was maintained at 240 °C for 5 h. The washing process was identical to that used for the pristine Ni_3_S_4_.

### Synthesis of Ni_3_S_4_@RuIr and e-Ni_3_S_4_@RuIr

For e-Ni_3_S_4_@RuIr synthesis, a 100 mL Schlenk tube with a stirring bar was charged with the e-Ni_3_S_4_ template (15 mg), Ru(acac)_3_ (0.630 mmol), Ir(acac)_3_ (0.245 mmol), and OAm (25 mL), vacuumed in an oil bath at 80 °C for 20 min, and charged with Ar (1 atm). Thereafter, the tube was transferred to a preheated oil bath and held at 240 °C for 2 h. The dark precipitate was sequentially washed with toluene (10 mL) and methanol (10 mL) and collected by centrifugation. This process was repeated twice. Ni_3_S_4_@RuIr with a Ru/Ir gradient core@shell atomic configuration was prepared using the pristine Ni_3_S_4_ template instead of the e-Ni_3_S_4_ template.

### Synthesis of (RuIr)O_2_/C and RuO_2_@IrO_2_/C

The (RuIr)O_2_/C and RuO_2_@IrO_2_/C were prepared using the heat treatment and oxidative conditions described for e-Ni_3_S_4_@RuIr and Ni_3_S_4_@RuIr, respectively. The as-prepared e-Ni_3_S_4_@RuIr and Ni_3_S_4_@RuIr were mixed with Vulcan carbon (XC-72R) in chloroform to achieve a catalyst loading of 20 wt%. The composites were collected by centrifugation and stored in a vacuum chamber. The dried powders were annealed at 400 °C for 2 h in a flow of N_2_-balanced 40% O_2_ (0.5 mL min^−1^) inside a tube furnace.

### Synthesis of home-made RuO_2_ NPs/C

The home-made RuO_2_ nanoparticles (NPs) were synthesized using a previously reported method with minor modifications^14,16^. To prepare home-made RuO_2_ NPs, Ru NPs were synthesized first. A slurry of Ru(acac)_3_ (0.06 mmol), CTAC (0.01 mmol), 1,2-hexadecanediol (0.4 mmol), and OAm (10 mmol) was prepared in a 100 mL Schlenk tube equipped with a magnetic stirrer. The slurry was vacuumed in an oil bath at 30 ^o^C for 10 min and charged with Ar (1 atm). Thereafter, the tube was transferred to a preheated oil bath and held at 280 ^o^C for 2 h. The product was sequentially washed with ethanol (15 mL) and toluene (10 mL) and collected by centrifugation. The resulting Ru NPs were supported on carbon black and thermally annealed at 400 ^o^C for 2 h in a flow of N_2_-balanced 40% O_2_ (0.5 mL min^−1^) inside a tube furnace, which was identical to the experimental method described above to prepare (RuIr)O_2_/C and RuO_2_@IrO_2_/C.

### Preparation of the working electrode

To prepare the working electrode, 5 mg of the carbon-supported catalyst was mixed with 125 µL of isopropanol, 25 µL of Nafion (5 wt%, Alfa-Aesar), and 350 µL of deionized water. The mixture was sonicated in an ice bath for 30 min to obtain a well-dispersed catalyst ink.

Prior to loading the catalyst ink, the rotating disk electrode (RDE) was polished on a micro-cloth pad using 1 μm of alumnia suspension (AS-100, Alpha alumina, MOHS 9) and 1 μm of monocrystalline diamond suspension (MetaDi) solutions. Subsequently, 7 µL of the catalyst ink was drop-cast onto a glassy carbon electrode (5.0 mm diameter; disk geometric area: 0.1963 cm^2^) to serve as the working electrode. The resulting mass loading of the catalyst on the electrode was 356.59 µg cm^−2^. Considering the nanoparticles were supported on the carbon support at 20 wt%, the total metal mass loading on the electrode area was calculated to be 71 µg cm^−2^, including a noble metal loading of 50 µg_Ru+Ir_ cm^−2^. The electrode was then vaccum-dried at room temperature before use.

For stability evluations using the choronopotentiometric method, 28 µL of the catalyst ink was drop-cast onto the electrode, resulting in a noble metal loading of 200 µg_Ru+Ir_ cm^−2^.

### Electrochemical characterization

Electrochemical measurements were performed at room temperature in a typical three-electrode cell using a CHI750E electrochemical analyzer (Bi-Potentiostat, CH Instruments) with N_2_-saturated 0.1 M HClO_4_ electrolyte. An SVC-3 voltammetry cell with teflon cap (BAS Inc.) was used, and freshly prepared 0.1 M HClO_4_ electrolyte was utilized for each measurement. A graphite carbon rod (6 mm, Qrins) served as the counter electrode, and an Ag/AgCl electrode (saturated 3 M NaCl, ALS Co., Ltd) was used as the reference electrode.

All of the potentials in this work are reported relative to the reversible hydrogen electrode (RHE) scale. The potentials were converted using the equation:

$${{{{\rm{E}}}}}_{{{{\rm{RHE}}}}}={{{{\rm{E}}}}}_{{{{\rm{mea}}}}}+{{{{\rm{E}}}}}_{{{{\rm{Ag}}}}/{{{\rm{AgCl}}}}}+0.0591\times {{{\rm{pH}}}},$$

where E_RHE_ is the potential on the RHE scale, E_mea_ is the meausred experimental potential, and E_Ag/AgCl_ is the reference electrode potential (0.1976 V_RHE_). The pH of the 0.1 M HClO4 electrolyte was measured to be 1.02 on average, based on eight measurements using a PHS-3D-02 pH meter (Shanghai San-Xin Instrumentation, Inc.). Addtionally, all polarization curves were corrected for 100% iR-compensation.

Before evaluating OER performance in the half-cell system, the electrolyte was purged with high-purity N_2_ gas (99.999%) for 30 min. Electrochemical pretretment was conducted by performing 20 cyclic voltammetry (CV) cycles in the potential range of 0.05–1.1 V_RHE_ at a scan rate of 0.2 V s^−1^ to clean and stabilize the catalyst surface. OER activity was assessed using linear sweep voltammetry (LSV) in the potential range of 1.1–1.8 V_RHE_ at a scan rate of 5 mV s^−1^ and an electrode rotation speed of 1600 rpm.

Electrochemical impedance spectroscopy (EIS) was performed in the frequency range of 100 kHz to 0.1 Hz using a small AC perturbation with a 5 mV amplitude at a fixed potential of 1.45 V_RHE_. The Nyquist plot was used to determine the solution resistance (R_S_), identified as x-axis intercept in the high-frequency region. At high frequencies, contributions from charge transfer resistance and double-layer capacitance are negligible, leaving only the solution resistance. The measured R_S_ was used for iR-compensation to correct for voltage lossess due to solution resistance. In our experiments, R_S_ was determined to be 12 ${{{\boldsymbol{\pm }}}}$ 0.3 Ω.

Stability was evalutated using chronopotentiometry at a constant current density of 100 mA cm^−2^ for (RuIr)O_2_/C and 10 mA cm^−2^ for RuO_2_@IrO_2_/C and home-made RuO_2_/C, respectively, in N_2_-saturated 0.1 M HClO_4_.

The electrochemical surface area (ECSA) was determined from the double-layer capacitance (*C*_dl_), which was calculated by recording CV curves at different scan rates (20, 40, 80, 100, 150, and 200 mV s^−1^) in the non-faradaic potential region (0.30–0.48 V_RHE_). The charging current (*i*_*c*_) at the center potential was evaluated as the product of the scan rate (*v*) and *C*_dl_:

$${i}_{c}=v{C}_{{{{\rm{dl}}}}}.$$

The ECSA was derived using the equation:

$${{{\rm{ECSA}}}}={C}_{{{{\rm{dl}}}}}/{C}_{{{{\rm{s}}}}},$$

where *C*_s_ = 0.035 mF cm^−2^.

The turnover frequency (TOF) was calculated using the equation:

$${{{\rm{TOF}}}}=(J\times A\times {{{\boldsymbol{\zeta }}}})/(4\times F\times {n}_{{{{\rm{mass}}}}}),$$

where *J* is the current density at 250 mV, *A* is the geometric area of the electrode, ${{{\boldsymbol{\zeta }}}}$ is the faradaic efficiency (assumed to equal 100%), *F* is the Faraday constant, and *n*_mass_ is the number of moles of active sites (Ru and Ir), determined from the ECSA. The total mass of Ru and Ir was calculated using:

$${n}_{{{{\rm{mass}}}}}=({n}_{{{{\rm{Ru}}}}+{{{\rm{Ir}}}}}{{\times }}{N}_{{{{\rm{A}}}}})/({M}_{{{{\rm{W}}}}})$$

where *n*_Ru+Ir_ is the total mass of Ru and Ir loaded onto the electrode, *N*_A_ is Avogadro’s constant, and *M*_W_ is the molecular weight.

### PEMWE single-cell test

(RuIr)O_2_/C or comm. IrO_2_ (99.99%, Alfa Aesar, USA) and Pt/C (46.9% Pt, Tanaka, Japan) were used as the anode and cathode catalysts, respectively. All tests were performed with an MEA prepared using the catalyst spray-coated membrane method. The catalyst slurry was prepared by mixing the catalyst with deionized water, the Nafion ionomer, and isopropanol, followed by sonication for 30 min at 40 °C. A Nafion 212 membrane, composed of entirely of Nafion polymer and with a nominal thcikness of approximately 50 μm, was used. For single-cell testing, the catalysts were uniformly spray-coated onto a 4 cm^2^ active area of a 25 cm^2^ membrane using a spray machine. The loadings were (RuIr)O_2_/C at 0.25–1 mg_Ru+Ir_ cm^−^², commercial IrO_2_ at 0.25 mg_Ir_ cm^−^², and Pt/C at 1 mg_Pt_ cm^−^². The MEA assembly included end plates, bipolar plate [Pt-coated Ti (anode) and graphite (cathode)], a porous transport layer (PTL; Ti felt (2GDL9N-025, Beakart, Belgium) for the anode and carbon paper (39BB, SGL Carbon, Germany) for the cathode), and gaskets. The Ti felt was pretreated with 5% oxalic acid at 60 °C for 30 min prior to use. The MEA was pressed with the PTLs using a force of 1 metric ton for 1 min at 120 °C. Single-cell tests were performed under ambient pressure with a continuous supply of clean distilled water. The active area and cell temperature were 4 cm^2^ and 80 °C, respectively. Electrochemical analysis was performed using an HCP-803 instrument (Bio-Logic, France). Tests were conducted using activation, LSV, and EIS. Activation was performed using chronoamperometry for 10 min at 1.5 V. LSV measurements were performed at a scan rate of 10 mV s^−1^ in the range of 1.2–2.0 V. EIS measurements were conducted in the frequency range of 10 kHz to 10 mHz at 2.0 V and an amplitude of 10 mV. Stability tests were conducted at a constant current density of 1.0 A cm^−2^.

### Material characterization

TEM and HRTEM analyses were carried out using a TECNAI G2 20 S-twin instrument operating at 200 kV and a TECNAI G2 F30ST instrument at 300 kV, respectively. Aberration-corrected imaging and high-resolution EDS measurements were performed using a Titan Probe Cs TEM (300 kV) with chemi-STEM capabilities at the FEI Nanoport in Eindhoven. EDS elemental mapping utilized a high-efficiency Super-X detection system with an XFEG electron source, incorporating four silicon drift detectors for enhanced detection near the analyzed region. Compared to standard EDS detectors with Schottky FEG sources, the chemi-STEM setup with X-FEG and the Super-X detector improved X-ray generation by up to fivefold and X-ray collection by up to tenfold. All STEM images and compositional maps were obtained using HAADF-STEM. PXRD patterns were measured on a Rigaku Ultima III diffractometer with Cu *K*_*α*_ radiation (graphite monochromatized) at 48 kV and 40 mA. XPS measurements were performed using an ULVAC-PHI X-tool instrument with a monochromatic AI *K*_*α*_ radiation source (1486.6 eV) operating at 24.1 W, referencing the C 1 s peak at 284.5 eV.

### In situ XAFS measurements

In situ XANES and EXAFS measurements for the Ru K-edge and Ir L3-edge were performed at the 7D beamline of PLS-II, located at the Pohang Accelerator Laboratory in the Republic of Korea. Monochromatic X-rays were generated using a double-crystal monochromator equipped with Si (111) crystals for the energy scans. The XAS experiments were conducted in a fluorescence-transmission geometry, where fluorescence mode was used to record sample spectra, and the reference material’s spectrum was simultaneously measured in transmission mode at room temperature. Calibration of the Ru K-edge and Ir L_3_-edge XANES spectra was achieved using Ru foil and Ir black powder at 22,117 eV and 11,215 eV, respectively. The raw XAS data were analyzed using the ATHENA software, with the Ru K-edge position determined through the first-derivative method and the Ir L3-edge white-line position identified using the second-derivative method.

### EXAFS measurements

EXAFS data were analyzed following established protocols using the ATHENA module within the IFEFFIT software suite^62^. The *k*^*3*^- weighted EXAFS spectra were obtained by removing the post-edge background from the total adsorption and normalizing it relative to the edge-jump step. The *k*^*3*^-weighted *χ*(*k*) data for the Ru K-edge and Ir L_3_-edge were then Fourier-transformed into real space using a Hanning window with a *dk* value of 1.0 Å^−1^, allowing separation of the contributions from distinct coordination shells. To evaluate the structural environment around the central atoms, least-squares fitting was carried out using the ARTEMIS module in conjunction with the FEFF6 ab initio code. Theoretical models for EXAFS fitting were developed using reference materials, with RuO_2_ and IrO_2_ serving as the basis for the Ru–O and Ir–O scattering paths, respectively.

### Differential electrochemical mass spectroscopy (DEMS)

In-situ DEMS using H_2_^18^O was conducted with an HPR-40 quadrupole mass spectrometer system (HIDEN Analytical Limited, England) and Type A cell to investigate the extent of the lattice oxygen mechanism during the OER^58,63^. Catalysts were deposited on polished glassy carbon (GC) electrodes (5 mm in diameter) at a loading of 40 µg cm^−2^. The setup included a GC electrode as the working electrode, an Ag/AgCl electrode as the reference, and a Pt wire were as the counter electrode. The ^18^O isotope labeling of the catalysts was achieved through 5 cyclic voltammetry (CV) cycles at a scan rate of 5 mV s^−1^ in 0.1 M HClO_4_ containing H_2_^18^O. During the labeling process, the electrolyte was circulated through the cell at a flow rate of 0.9 mL s^−1^. For RuIrO_2_, the CV potential range was 1.25–1.65 V_RHE_, and for RuO_2_, it was 1.25–1.95 V_RHE_, ensuring comparable current densities. The electrodes were subsequently rinsed several times with H_2_^16^O to remove any remaining H_2_^18^O. Finally, the labeled electrodes were placed in 0.1 M HClO_4_ with H_2_^16^O, and CV was performed within the same potential ranges. During the OER, ^32^O_2_ (^16^O^16^O) and ^34^O_2_ (^16^O^18^O) generated were monitored using mass spectrometry, with baseline correction applied to the signals.

## Supplementary information

Supplementary Information

Description of Additional Supplementary Files

Supplementary Data 1

Transparent Peer Review file

## Source data

Source Data

## Data Availability

The data that support the findings and conclusions generated in this study are provided in the main article and the Supplementary Information. Source data are provided with this paper.

## References

[1] Seh, Z. W. et al. Combining theory and experiment in electrocatalysis: insights into materials design. *Science* **355**, eaad4998 (2017). 28082532 10.1126/science.aad4998

[2] Seitz, L. C. et al. A highly active and stable IrO_x_/SrIrO_3_ catalyst for the oxygen evolution reaction. *Science* **353**, 1011–1014 (2016). 27701108 10.1126/science.aaf5050

[3] Jin, H. et al. Nanocatalyst Design for Long-Term Operation of Proton/Anion Exchange Membrane Water Electrolysis. *Adv. Energy Mater.* **11**, 2003188 (2021).

[4] Strickler, A. L. et al. Systematic investigation of iridium-based bimetallic thin film catalysts for the oxygen evolution reaction in acidic media. *ACS Appl. Mater. Interfaces* **11**, 34059–34066 (2019). 31442022 10.1021/acsami.9b13697

[5] Spori, C. et al. Experimental activity descriptors for iridium-based catalysts for the electrochemical oxygen evolution reaction (OER). *ACS Catal.* **9**, 6653–6663 (2019).

[6] Geiger, S. et al. The stability number as a metric for electrocatalyst stability benchmarking. *Nat. Catal.* **1**, 508–515 (2018).

[7] Laha, S. et al. Ruthenium oxide nanosheets for enhanced oxygen evolution catalysis in acidic medium. *Adv. Energy Mater.* **9**, 1803795 (2019).

[8] Zhao, Z. L. et al. Boosting the oxygen evolution reaction using defect-rich ultra-thin ruthenium oxide nanosheets in acidic media. *Energy Environ. Sci.* **13**, 5143–5151 (2020).

[9] Kim, J. et al. High-performance pyrochlore-type yttrium ruthenate electrocatalyst for oxygen evolution reaction in acidic media. *J. Am. Chem. Soc.* **139**, 12076–12083 (2017). 28749136 10.1021/jacs.7b06808

[10] Zagalskaya, A. & Alexandrov, V. Role of defects in the interplay between adsorbate evolving and lattice oxygen mechanism of the oxygen evolution reaction in RuO_2_ and IrO_2_. *ACS Catal.* **10**, 3650–3657 (2020).

[11] Hao, S. et al. Dopants fixation of ruthenium for boosting acidic oxygen evolution stability and activity. *Nat. Commun.* **11**, 5368 (2020). 33097730 10.1038/s41467-020-19212-y PMC7584605

[12] Dickens, C. F. & Norskov, J. K. A theoretical investigation into the role of surface defects for oxygen evolution on RuO_2_. *J. Phys. Chem. C.* **121**, 18516–18524 (2017).

[13] Klyukin, K., Zagalskaya, A. & Alexandrov, V. Role of dissolution intermediates in promoting oxygen evolution reaction at RuO_2_(110) surface. *J. Phys. Chem. C.* **123**, 22151–22157 (2019).

[14] Jin, H. et al. Safeguarding the RuO_2_ phase against lattice oxygen oxidation during acidic water electrooxidation. *Energy Environ. Sci.* **15**, 1119–1130 (2022).

[15] Kwon, T. et al. Interfacing RuO_2_ with Pt to induce efficient charge transfer from Pt to RuO_2_ for highly efficient and stable oxygen evolution in acidic media. *J. Mater. Chem. A* **9**, 14352–14362 (2021).

[16] Oh, A. et al. Topotactic transformations in an lcosahedral nanocrystal to form efficient water-splitting catalysts. *Adv. Mater.* **31**, 1805546 (2019). 10.1002/adma.201805546 30362625

[17] Jun, M., Kwon, T., Son, Y., Kim, B. & Lee, K. Chemical Fields: Directing Atom Migration in the Multiphasic Nanocrystal. *Acc. Chem. Res.* **55**, 1015–1024 (2022). 35263076 10.1021/acs.accounts.1c00745

[18] Luo, M. & Guo, S. Strain-controlled electrocatalysis on multimetallic nanomaterials. *Nat. Rev. Mater.* **2**, 17059 (2017).

[19] Kim, J., Jun, M., Choi, S., Jo, J. & Lee, K. Reactive nanotemplates for synthesis of highly efficient electrocatalysts: beyond simple morphology transfer. *Nanoscale* **11**, 20392–20410 (2019). 31651011 10.1039/c9nr05750a

[20] Chen, C. et al. Highly crystalline multimetallic nanoframes with three-dimensional electrocatalytic surfaces. *Science* **343**, 1339–1343 (2014). 24578531 10.1126/science.1249061

[21] Oh, H.-S., Nong, H. N., Reier, T., Gliech, M. & Strasser, P. Oxide-supported Ir nanodendrites with high activity and durability for the oxygen evolution reaction in acid PEM water electrolyzers. *Chem. Sci.* **6**, 3321–3328 (2015). 28706696 10.1039/c5sc00518c PMC5490338

[22] Audichon, T. et al. IrO_2_ coated on RuO_2_ as efficient and stable electroactive nanocatalysts for electrochemical water splitting. *J. Phys. Chem. C.* **120**, 2562–2573 (2016).

[23] Lin, Y. et al. Chromium-ruthenium oxide solid solution electrocatalyst for highly efficient oxygen evolution reaction in acidic media. *Nat. Commun.* **10**, 162 (2019). 30635581 10.1038/s41467-018-08144-3 PMC6329788

[24] Kotz, R. & Carlen, M. Principles and applications of electrochemical capacitors. *Electrochim. Acta* **45**, 2483–2498 (2000).

[25] Chen, H., Zhang, X., Geng, S., Song, S. & Wang, Y. Modulating the electronic structure of RuO_2_ through Cr solubilizing for improved oxygen evolution reaction. *Small Methods* **6**, 2200636 (2022). 10.1002/smtd.202200636 35879051

[26] Zhang, Y. et al. Mo-doped mesoporous RuO_2_ spheres as high-performance acidic oxygen evolution reaction electrocatalyst. *Small* **20**, 2305889 (2023). 10.1002/smll.202305889 37939307

[27] Chen, S. et al. Mn-doped RuO_2_ nanocrystals as highly active electrocatalysts for enhanced oxygen evolution in acidic media. *ACS Catal.* **10**, 1152–1160 (2020).

[28] Wang, K. et al. Highly active ruthenium sites stabilized by modulating electron-feeding for sustainable acidic oxygen-evolution electrocatalysis. *Energy Environ. Sci.* **15**, 2356–2365 (2022).

[29] Jin, H. et al. Dynamic rhenium dopant boosts ruthenium oxide for durable oxygen evolution. *Nat. Commun.* **14**, 354 (2023). 36681684 10.1038/s41467-023-35913-6 PMC9867741

[30] Zhu, W. et al. Direct dioxygen radical coupling driven by octahedral ruthenium-oxygen-cobalt collaborative coordination for acidic oxygen evolution reaction. *J. Am. Chem. Soc.* **145**, 17995–18006 (2023). 37550082 10.1021/jacs.3c05556

[31] Wu, Z.-Y. et al. Non-iridium-based electrocatalyst for durable acidic oxygen evolution reaction in proton exchange membrane water electrolysis. *Nat. Mater.* **22**, 100–108 (2023). 36266572 10.1038/s41563-022-01380-5

[32] Harzandi, A. M. et al. Ruthenium core-shell engineering with nickel single atoms for selective oxygen evolution via nondestructive mechanism. *Adv. Energy Mater.* **1**, 2003448 (2021).

[33] Xue, Y. et al. Sulfate-functionalized RuFeO_x_ as highly efficient oxygen evolution reaction electrocatalyst in acid. *Adv. Funct. Mater.* **31**, 2101405 (2021).

[34] Wang, J. et al. Single-site Pt-doped RuO_2_ hollow nanospheres with interstitial C for high-performance acidic overall water splitting. *Sci. Adv.* **8**, eabl9271 (2022). 35235348 10.1126/sciadv.abl9271 PMC8890715

[35] Qin, Y. et al. RuO_2_ electronic structure and lattice strain dual engineering for enhanced acidic oxygen evolution reaction performance. *Nat. Commun.* **13**, 3784 (2022). 35778401 10.1038/s41467-022-31468-0 PMC9249734

[36] Zhang, L. et al. Sodium-decorated amorphous/crystalline RuO_2_ with rich oxygen vacancies: a robust pH-universal oxygen evolution electrocatalyst. *Angew. Chem.* **133**, 18969–18977 (2021). 10.1002/anie.202106631 34121280

[37] Huang, K. et al. Ru/Se-RuO_2_ composites via controlled selenization strategy for enhanced acidic oxygen evolution. *Adv. Funct. Mater.* **33**, 2211102 (2023).

[38] Lee, K. et al. Modulating the valence electronic structure using earth-abundant aluminum for high-performance acidic oxygen evolution reaction. *Chem* **9**, 3600–3612 (2023).

[39] Shan, J. et al. Charge-redistribution-enhanced nanocrystalline Ru@IrO_x_ electrocatalysts for oxygen evolution in acidic media. *Chem* **5**, 445–459 (2019).

[40] Shan, J., Ling, T., Davey, K., Zheng, Y. & Qiao, S.-Z. Transition-metal-doped RuIr bifunctional nanocrystals for overall water splitting in acidic environments. *Adv. Mater.* **2019**, 1900510 (2019). 10.1002/adma.201900510 30811671

[41] Zhang, J. et al. Iridium nanoparticles anchored on 3D graphite foam as a bifunctional electrocatalyst for excellent overall water splitting in acidic solution. *Nano Energy* **40**, 27–33 (2017).

[42] Joo, J. et al. Mn-dopant differentiating the Ru and Ir oxidation states in catalytic oxides toward durable oxygen evolution reaction in acidic electrolyte. *Small Methods* **6**, 2101236 (2022). 10.1002/smtd.202101236 35041273

[43] Li, G., Li, S., Ge, J., Liu, C. & Xing, W. Discontinuously covered IrO_2_-RuO_2_@Ru electrocatalysts for the oxygen evolution reaction: how high activity and long-term durability can be simultaneously realized in the synergistic and hybrid nano-structure. *J. Mater. Chem. A* **5**, 17221–17229 (2017).

[44] Lin, C. et al. In-situ reconstructed Ru atom array on -MnO_2_ with enhanced performance for acidic water oxidation. *Nat. Catal.* **4**, 1012–1023 (2021).

[45] Siracusano, S., Bagli, V., Dijk, N. V., Merlo, L. & Arico, A. S. Enhanced performance and durability of low catalyst loading PEM water electrolyser based on a short-side chain perfluorosulfonic ionomer. *Appl. Energy* **192**, 477–489 (2017).

[46] Hegge, F. et al. Efficient and stable low iridium loaded anodes for PEM water electrolysis made possible by nanofiber interlayers. *ACS Appl. Energy Mater.* **3**, 8276–8284 (2020).

[47] Liu, H., Tao, H. B. & Liu, B. Kinetic insights of proton exchange membrane water electrolyzer obtained by operando characterization methods. *J. Phys. Chem. Lett.* **13**, 6520–6531 (2022). 35822838 10.1021/acs.jpclett.2c01341

[48] Clancy, J. P. et al. Spin-orbit coupling in iridium-based 5d compounds probed by x-ray absorption spectroscopy. *Phys. Rev. B* **86**, 195131 (2012).

[49] Choy, J.-H., Kim, D.-K., Demazeau, G. & Yung, D.-Y. L_III_-edge XANES study on unusually high valent iridium in a perovskite lattice. *J. Phys. Chem.* **98**, 6258–6262 (1994).

[50] Choy, J.-H., Kim, D.-K., Hwang, S.-H., Demazeau, G. & Jung, D.-Y. XANES and EXAFS studies on the Ir-O bond covalency in ionic iridium perovskites. *J. Am. Chem. Soc.* **117**, 8557–8566 (1995).

[51] Nong, H. N. et al. A unique oxygen ligand environment facilitates water oxidation in hole-doped IrNiO_x_ core-shell electrocatalysts. *Nat. Cat.* **1**, 841–851 (2018).

[52] Mo, Y. et al. In situ iridium L_III_-edge x-ray absorption and surface enhanced raman spectroscopy of electrodeposited iridium oxide films in aqueous electrolytes. *J. Phys. Chem. B* **106**, 3681–3686 (2002).

[53] Liu, H. et al. Iridium doped pyrochlore ruthenates for efficient and durable electrocatalytic oxygen evolution in acidic media. *Small* **18**, 2202513 (2022). 10.1002/smll.202202513 35780475

[54] Rao, R. R. et al. Towards identifying the active sites on RuO_2_(110) in catalyzing oxygen evolution. *Energy Environ. Sci.* **10**, 2626–2637 (2017).

[55] Hong, W. T. et al. Charge-transfer-energy-dependent oxygen evolution reaction mechanisms for perovskite oxides. *Energy Environ. Sci.* **10**, 2190–2200 (2017).

[56] Zhang, W. et al. Engineering d-p orbital hybridization through regulation of interband energy separation for durable aqueous Zn//VO_2_(B) batteries. *J. Chem. Eng.* **464**, 142711 (2023).

[57] Ge, R. et al. Ultrafine defective RuO_2_ electrocatalyst integrated on carbon cloth for robust water oxidation in acidic media. *Adv. Energy Mater.* **9**, 1901313 (2019).

[58] Ping, X. et al. Locking the lattice oxygen in RuO_2_ to stabilize highly active Ru sites in acidic water oxidation. *Nat. Commun.* **115**, 2501 (2024). 10.1038/s41467-024-46815-6 PMC10954744 38509091

[59] Wen, Y. et al. Stabilizing highly active Ru sites by suppressing lattice oxygen participation in acidic water oxidation. *J. Am. Chem. Soc.* **143**, 6482–6490 (2021). (65). 33891414 10.1021/jacs.1c00384

[60] Grimaud, A. et al. Activating lattice oxygen redox reactions in metal oxides to catalyse oxygen evolution. *Nat. Mater.* **9**, 457 (2017). 10.1038/nchem.2695 28430191

[61] Liu, Q., Diaz, A., Prosvirin, A., Luo, Z. & Batteas, J. D. Shape-controlled synthesis of nanopyramids and nanoprisms of nickel sulflide (Ni_3_S_4_). *Nanoscale* **6**, 8935–8942 (2014). 24965378 10.1039/c4nr01196a

[62] Song, S. et al. Operando X-ray spectroscopic tracking of self-reconstruction for anchored nanoparticles as high-performance electrocatalysts towards oxygen evolution. *Energy Environ. Sci.* **11**, 2945–2953 (2018).

[63] McCrory, C. C., Jung, S., Peters, J. C. & Jaramillo, T. F. Benchmarking heterogeneous electrocatalysts for the oxygen evolution reaction. *J. Am. Chem. Soc.* **135**, 16977–16987 (2013). 24171402 10.1021/ja407115p

