# Supplementary material for: Atomic-level Ru-Ir mixing in rutile-type (RuIr)O2 for efficient and durable oxygen evolution catalysis
Source: Nat Commun. 2025 Jan 10;16:579. doi: 10.1038/s41467-025-55910-1 (PMC11723980; doi:10.1038/s41467-025-55910-1)
Supplement: Supplementary file 1 — Supplementary Information [file 41467_2025_55910_MOESM1_ESM.pdf]

Supplementary Information

Atomic-Level Ru-Ir Mixing in Rutile-Type $(RuIr)O_2$ for Efficient and Durable Oxygen Evolution Catalysis

Yeji Park,[†1,2] Ho Yeon Jang,[†3] Tae Kyung Lee,[†2,4] Taekyung Kim,[†5] Doyeop Kim,[1] Dongjin Kim,[1] Hionsuck Baik,[5] Jinwon Choi,[6,7] Taehyun Kwon,*[6,7] Sung Jong Yoo,*[2,8] Seoin Back,*[3] and Kwangyeol Lee*[1]

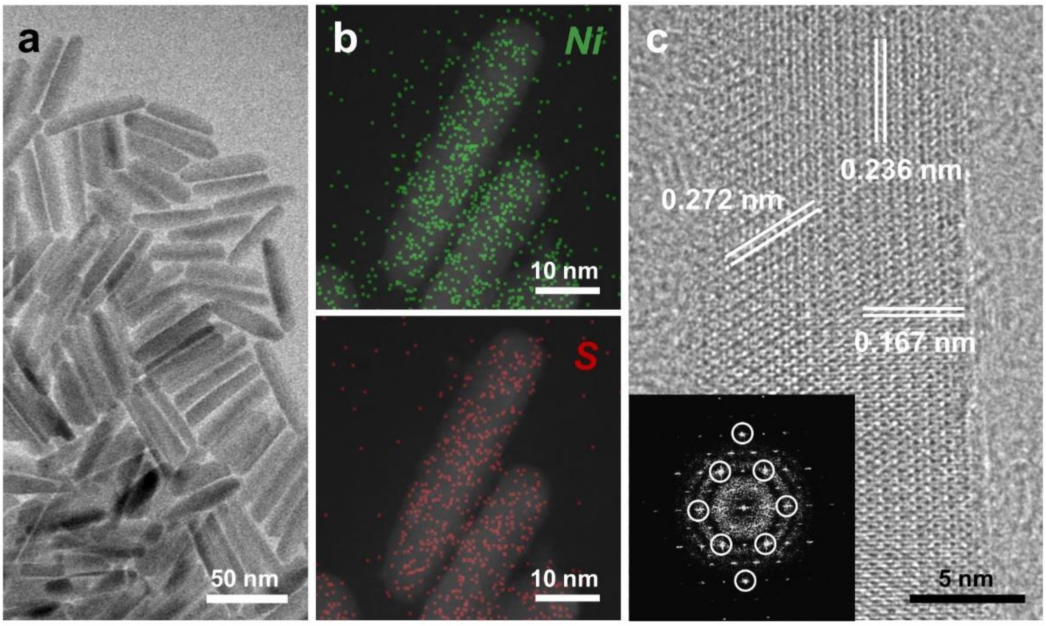

Supplementary Fig. 1 | Characterization of the pristine Ni_3S_4 template. a TEM image of the Ni_3S_4 from top view. **b** Combined HAADF and elemental mapping images for Ni (top, green) and S (bottom, red) contents in Ni_3S_4. **c** HRTEM image (inset: measured FFT patterns of Ni_3S_4, zone axis: [011]).

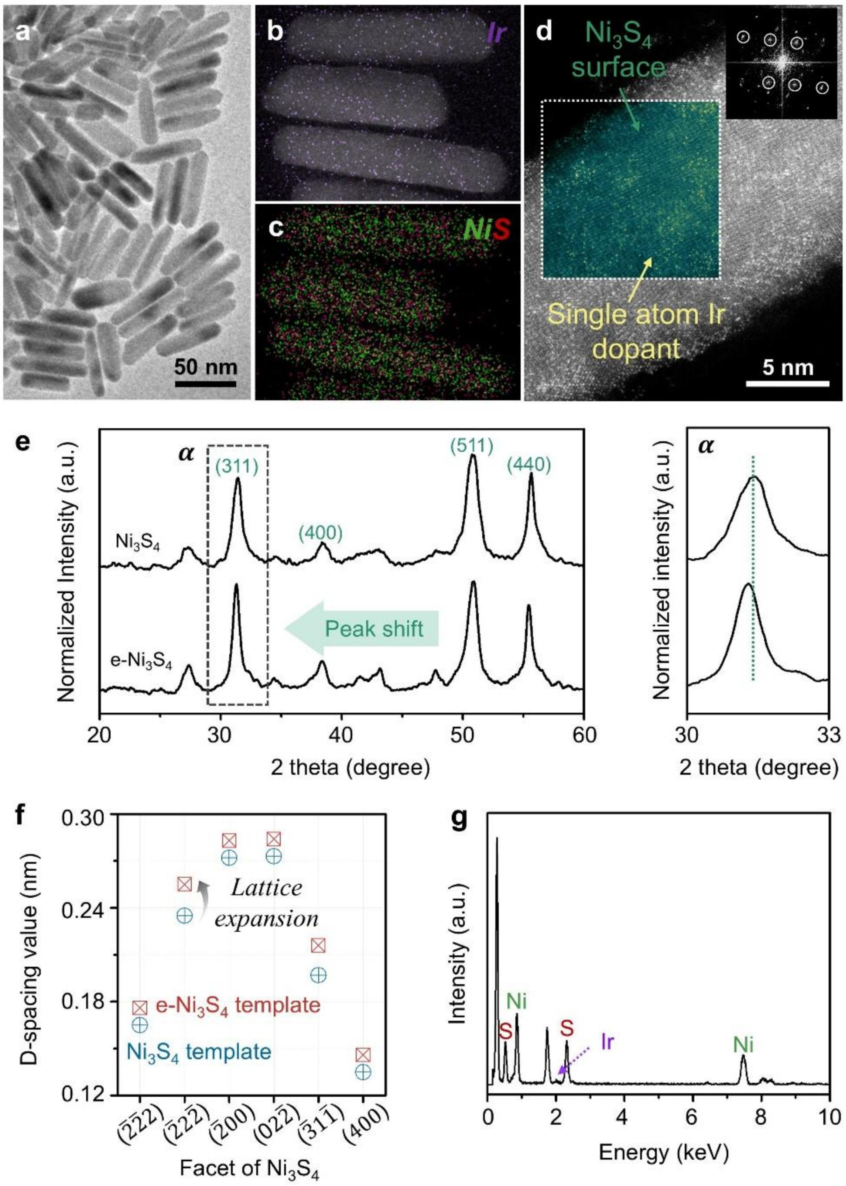

Supplementary Fig. 2 | Characterization of the e-Ni_3S_4 template. **a** TEM image of e-Ni_3S_4 from the top view. **b** Combined HAADF and elemental mapping images for Ir (purple). **c** Combined elemental mapping image for Ni (green) and S (red) contents in e-Ni_3S_4. **d** HAADF image showing single atom Ir dopant (yellow) on the Ni_3S_4 surface (green) (inset: measured FFT pattern of e-Ni_3S_4, zone axis: [011]). **e** PXRD patterns of Ni_3S_4 (top) and e-Ni_3S_4 (bottom). **f** Comparison of the d-spacing values of each facet of Ni_3S_4 phase in Ni_3S_4 (blue circle) and e-Ni_3S_4 (red square). **g** EDS spectrum of the e-Ni_3S_4. The sample for EDS measurement was prepared on a Mo TEM grid.

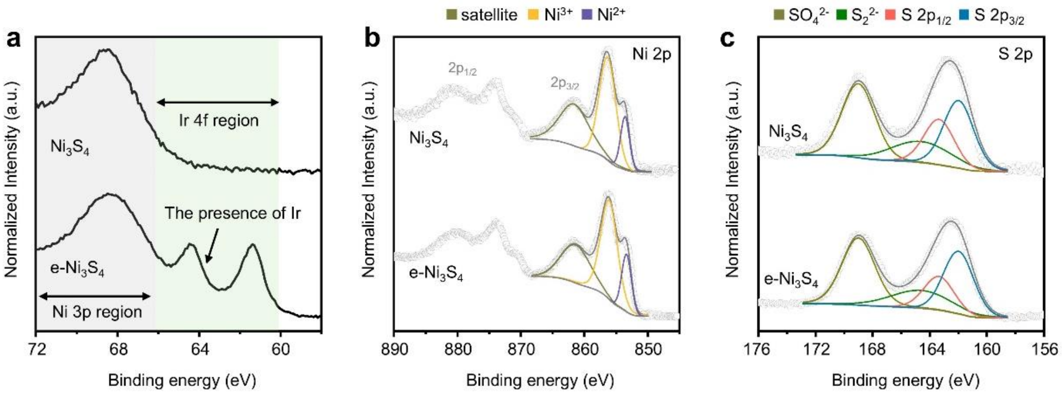

Supplementary Fig. 3 | Chemical state of the pristine Ni_3S_4 and e-Ni_3S_4 templates. a Ir 4f, **b** Ni 2p, and **c** S 2p XPS spectra. There was no difference in the chemical states of Ni and S in the two templates due to the Ir dopant.

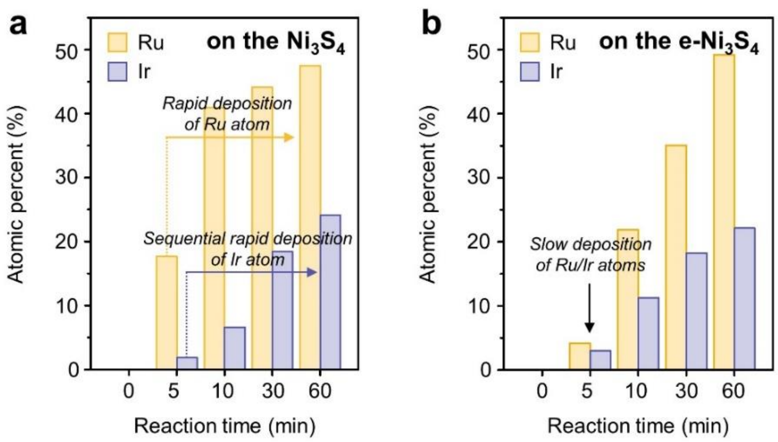

Supplementary Fig. 4 | Atomic composition analysis of the pristine Ni_3S_4 and e-Ni_3S_4 templates. Comparison of atomic composition of Ru and Ir atoms on the **a** Ni_3S_4 and **b** e-Ni_3S_4 templates depending on different reaction times from 0 min to 60 min, obtained by EDS measurement.

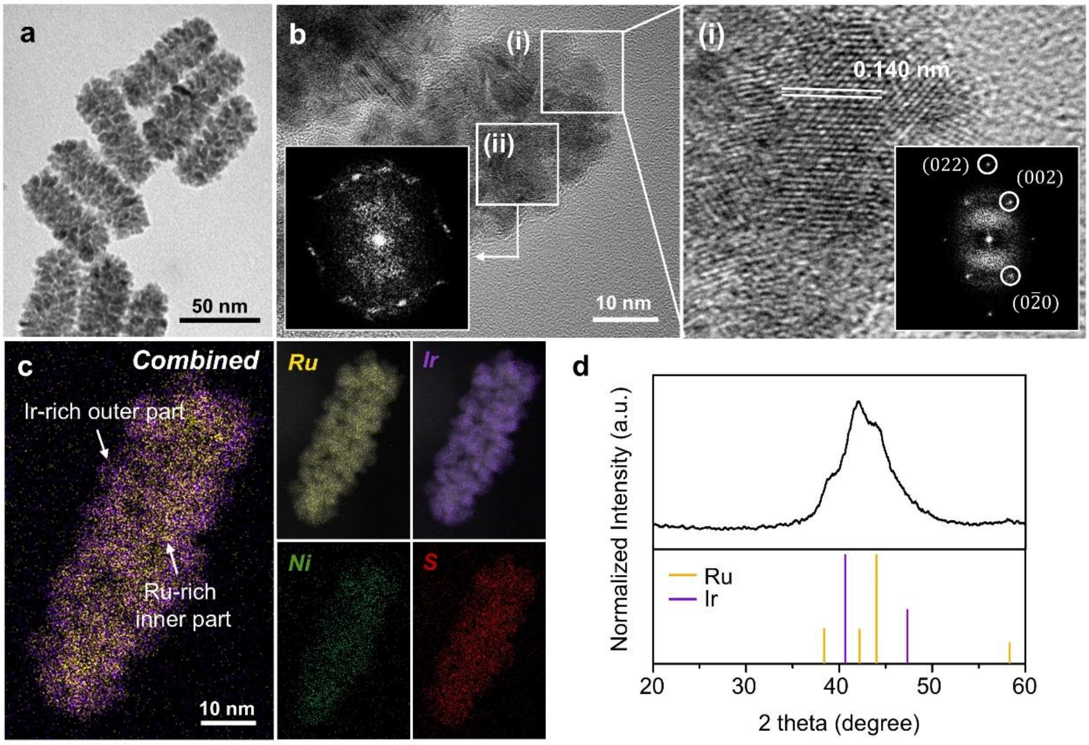

Supplementary Fig. 5 | Characterization of the Ni_3S_4@RuIr with the Ru inner shell and Ir outer shell configuration. a TEM image of Ni_3S_4@RuIr from the top view. **b** HRTEM and enlarged HRTEM images with corresponding FFT patterns. **c** Combined and corresponding elemental mapping images of Ru (yellow), Ir (purple), Ni (green), and S (red) contents in Ni_3S_4@RuIr. Elemental mapping images of Ru and Ir combined with HAADF image showing the atomic distribution within the nanoparticle. **d** PXRD patterns of Ni_3S_4@RuIr. The color bars indicate the PXRD lines for the reference: *hcp* Ru (yellow, #01-088-2333) and *fcc* Ir (purple, #06-0598).

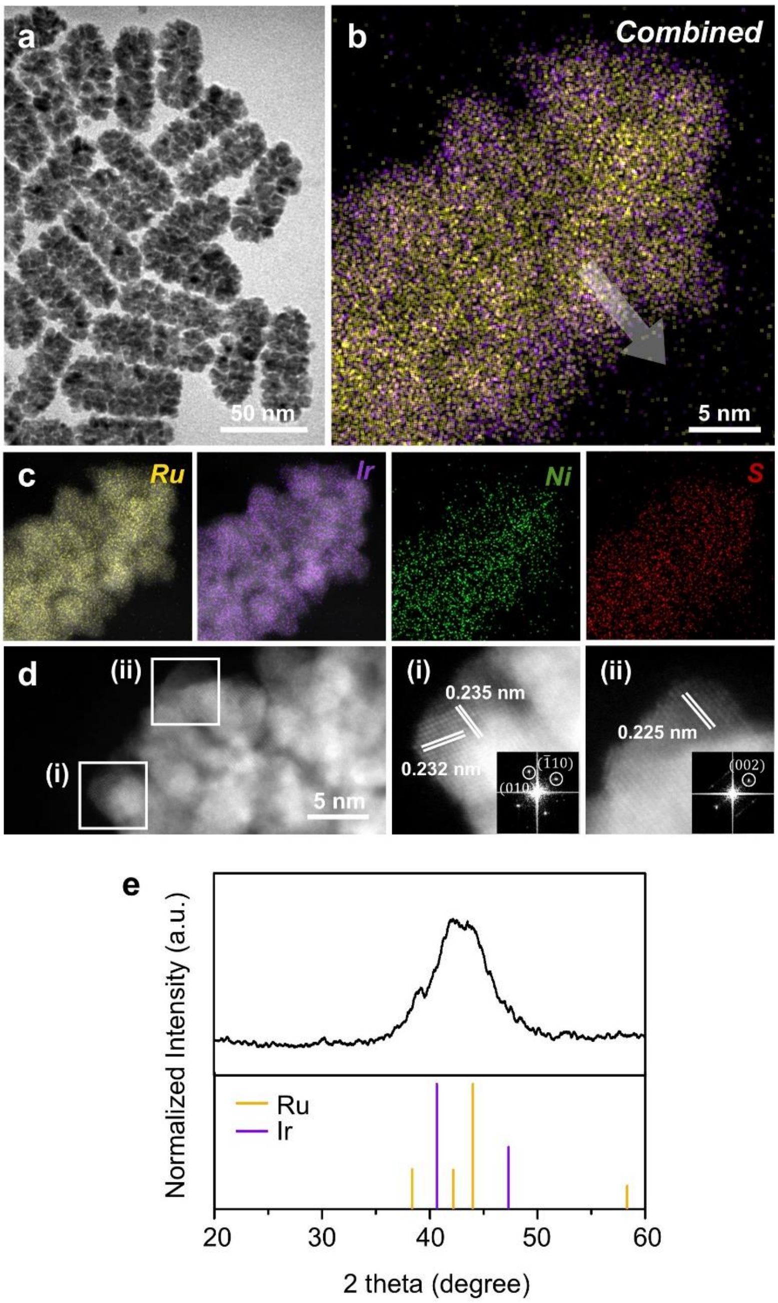

Supplementary Fig. 6 | Characterization of the e-Ni_3S_4@RuIr with RuIr alloy shell configuration. **a** TEM image of e-Ni_3S_4@RuIr from the top view. **b** Combined and **c** corresponding elemental mapping images of Ru (yellow), Ir (purple), Ni (green), and S (red) contents in e-Ni_3S_4@RuIr. Elemental mapping images of Ru and Ir combined with HAADF image showing the atomic distribution within the nanoparticle. **d** HAADF-STEM and enlarged HAADF-STEM images with corresponding FFT patterns. **e** PXRD patterns of Ni_3S_4@RuIr. The color bars indicate the PXRD lines for the reference: *hcp* Ru (yellow, #01-088-2333) and *fcc* Ir (purple, #06-0598).

Supplementary Note 1 | Characterization for the Ni_3S_4@RuIr and e-Ni_3S_4@RuIr.

TEM images of both Ni_3S_4@RuIr (Supplementary Fig. 5a) and e-Ni_3S_4@RuIr (Supplementary Fig. 6a) revealed that the Ru/Ir nanoparticles grew into a dendritic structure on the Ni_3S_4 and e-Ni_3S_4 templates, respectively, revealing an overall rod-like morphology. The HRTEM (Supplementary Fig. 5b) and HAADF-STEM (Supplementary Fig. 6d) images, along with the corresponding FFT patterns, confirmed the polycrystalline nature of dendritic RuIr nanoparticles in both Ni_3S_4@RuIr and e-Ni_3S_4@RuIr. Notably, the EDS elemental mapping images and line profile analysis showcased distinct Ru/Ir atomic distribution in the shells of the Ni_3S_4@RuIr and e-Ni_3S_4@RuIr. Specifically, Ni_3S_4@RuIr exhibited a dual-part structure with an Ir-rich outer region and a Ru-rich inner region on the Ni_3S_4 core (Supplementary Fig. 5c), whereas e-Ni_3S_4@RuIr displayed a homogeneous distribution of Ru and Ir atoms throughout the entire e-Ni_3S_4 core (Supplementary Fig. 6b-c).

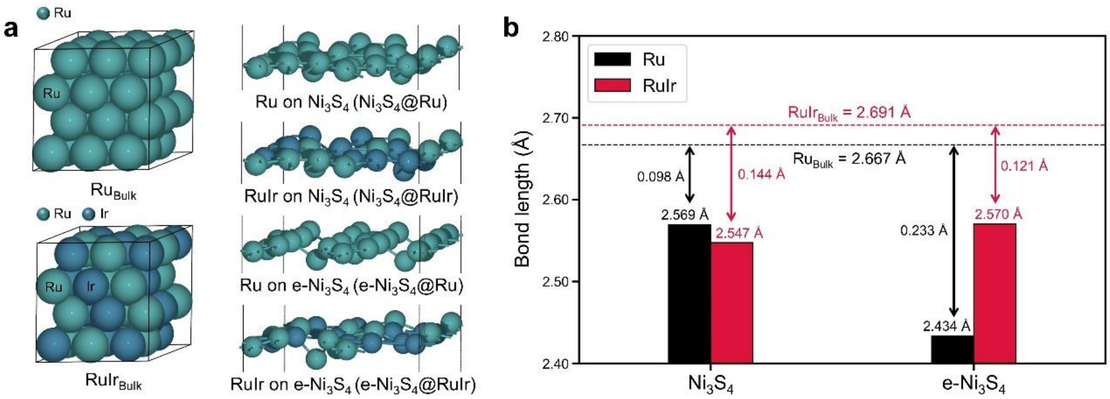

Supplementary Fig. 7 | DFT Calculations for interatomic bond length (d_{ave}). a The optimized bulk structures (Ru_{Bulk}, $RuIr_{Bulk}$) and shell structures (Ru on Ni_3S_4, RuIr on Ni_3S_4, Ru on e-Ni_3S_4, RuIr on e-Ni_3S_4). The shell structures were extracted from the optimized M (M = Ru, RuIr) on the Ni_3S_4 and e-Ni_3S_4 structures. Green and blue spheres represented Ru and Ir atoms, respectively. **b** The averaged interatomic bond lengths of metal and alloy shells on Ni_3S_4 and e-Ni_3S_4 cores.

Supplementary Note 2 | DFT calculations for interatomic bond length (d_{ave}).

We hypothesized that if the d_{ave} value at the interface between the template (core: Ni_3S_4, and e-Ni_3S_4) and the growing metal (shell: Ru only, and RuIr alloy) matches the d_{ave} value of the standard bulk (Ru_{Bulk} and $RuIr_{Bulk}$), then the growth of Ru or RuIr shell on each template would be preferred. In the case of Ru atoms, when they were grown on the Ni_3S_4 surface (2.569 Å), they exhibited a d_{ave} of 2.569 Å, similar to the standard Ru_{Bulk} (2.667 Å), suggesting instability in the growth of Ru on e-Ni_3S_4, indicating a lattice mismatch. In contrast, when the RuIr alloy shell was grown on the e-Ni_3S_4 surface, it displayed a 2.570 Å, which is similar to the standard $RuIr_{Bulk}$ (2.691 Å). Thus, these results suggest that the growth of the RuIr alloy shell on the e-Ni_3S_4 template is more thermodynamically favorable than the Ru shell.

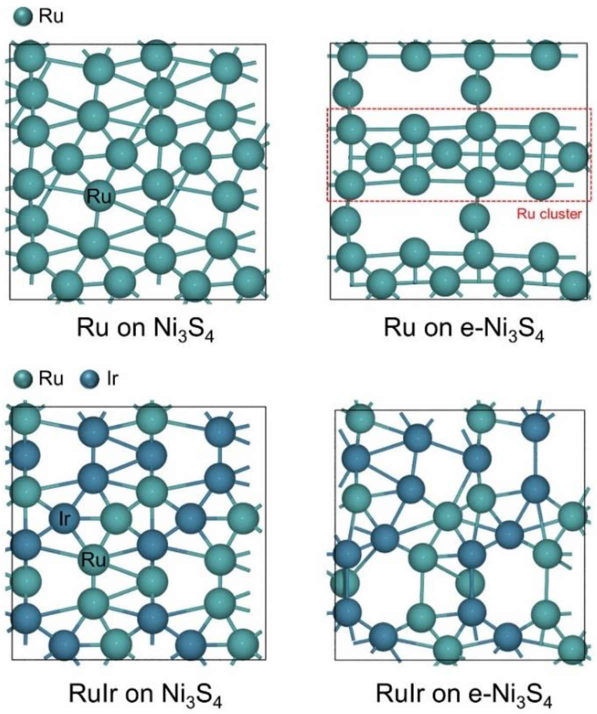

Supplementary Fig. 8 | Model structures for DFT calculations. The top view of optimized shell structures: Ru on Ni_3S_4, Ru on e-Ni_3S_4, RuIr on Ni_3S_4, and RuIr on e-Ni_3S_4. Green and blue spheres represented Ru and Ir atoms, respectively

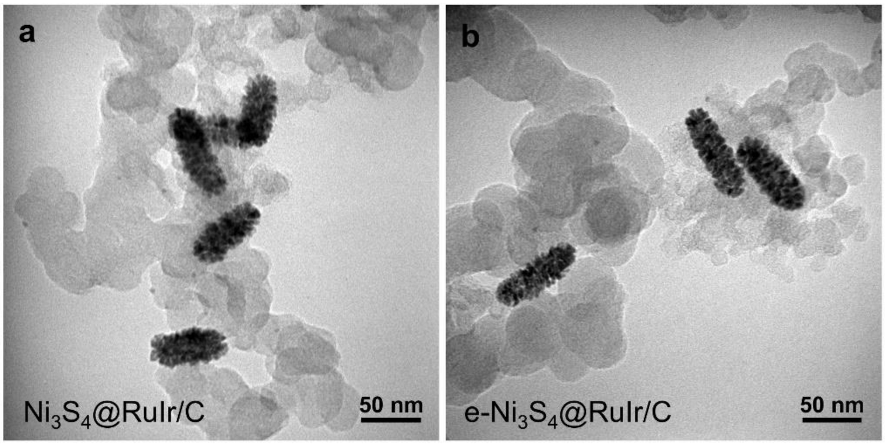

Supplementary Fig. 9 | TEM images. (a) Ni_3S_4@RuIr/C and (b) e-Ni_3S_4@RuIr/C.

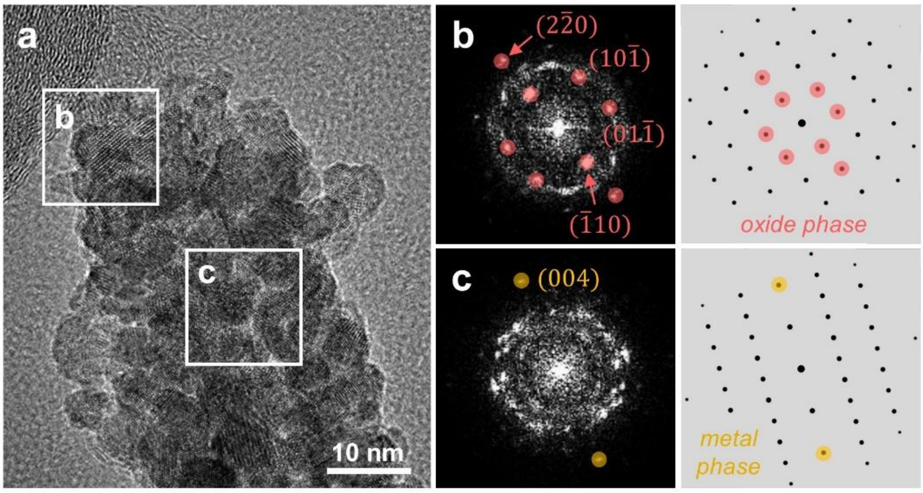

Supplementary Fig. 10 | Characterization of (RuIr)O_2/C. a Enlarged HRTEM image of (RuIr)O_2/C with corresponding FFT patterns for **b** oxide phase and **c** metal phase.

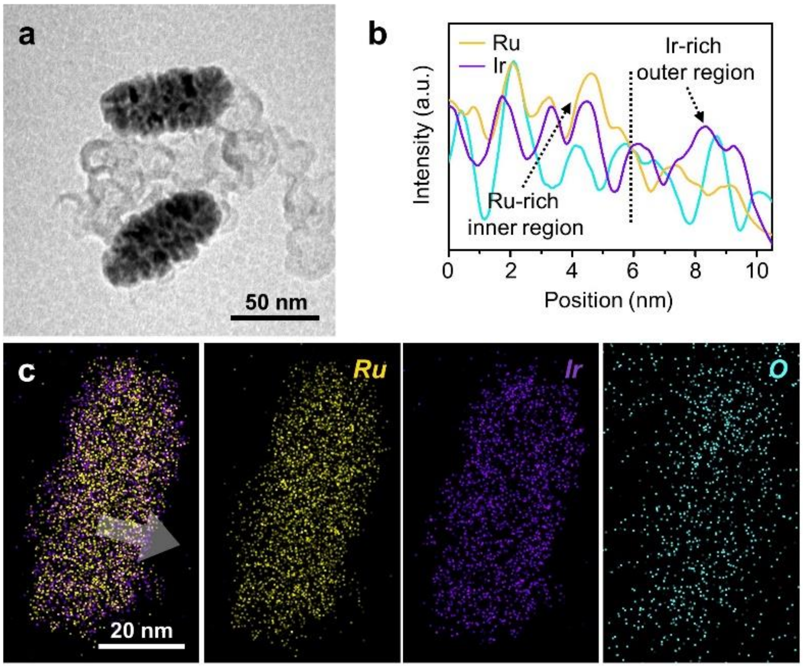

Supplementary Fig. 11 | Characterization of RuO_2@IrO_2/C. a TEM image of RuO_2@IrO_2/C from the top view. **b** Line profile analysis of RuO_2@IrO_2/C corresponding to the marked area indicated by the white arrow in Supplementary Fig. 11c. **c** Combined and corresponding EDS elemental mapping images of Ru (yellow), Ir (purple), and O (cyan) contents in RuO_2@IrO_2/C.

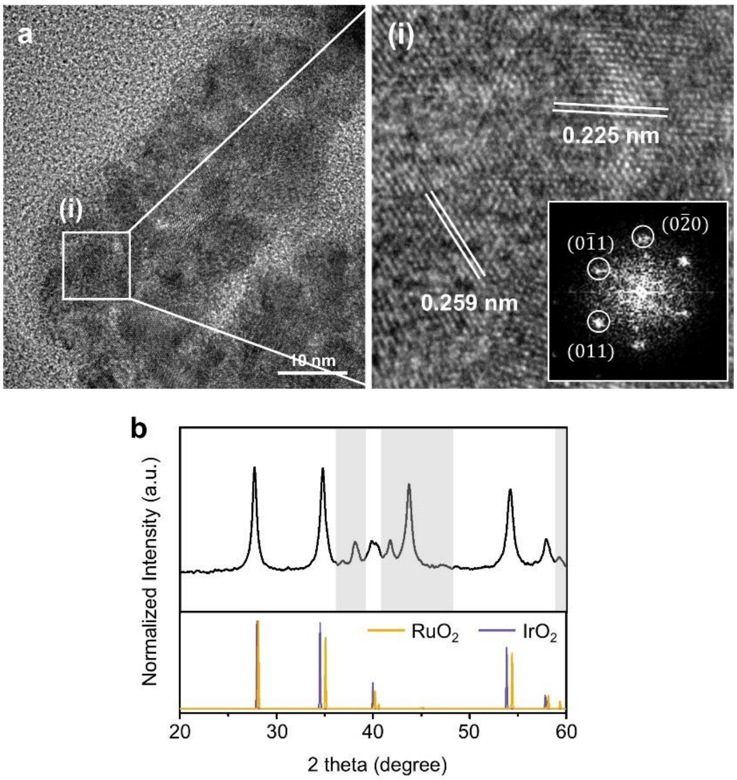

Supplementary Fig. 12 | Characterization of RuO_2@IrO_2/C. a HRTEM and enlarged HRTEM images with corresponding FFT patterns of RuO_2@IrO_2/C. **b** PXRD pattern of RuO_2@IrO_2/C. The gray box indicates the remaining metallic species in RuO_2@IrO_2/C.

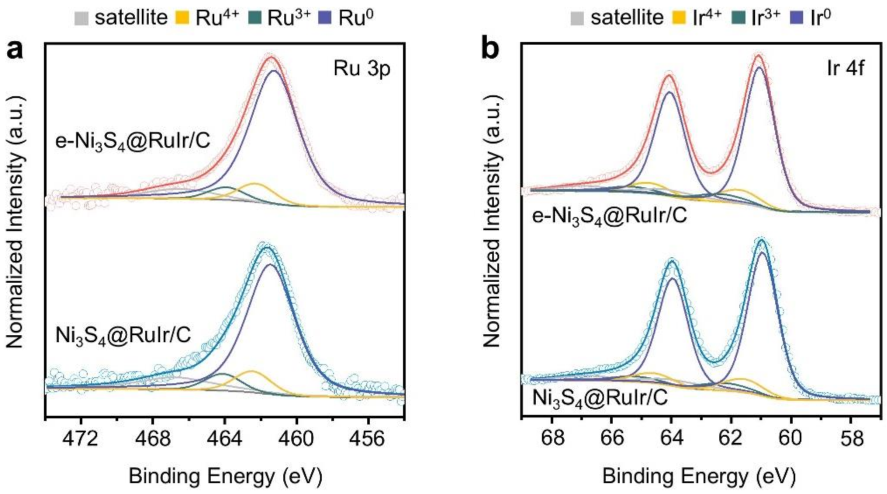

Supplementary Fig. 13 | XPS analysis. a Ru $3p_{3/2}$ and **b** Ir 4f XPS spectra of e-Ni_3S_4@RuIr/C (top, red) and Ni_3S_4@RuIr/C (bottom, blue).

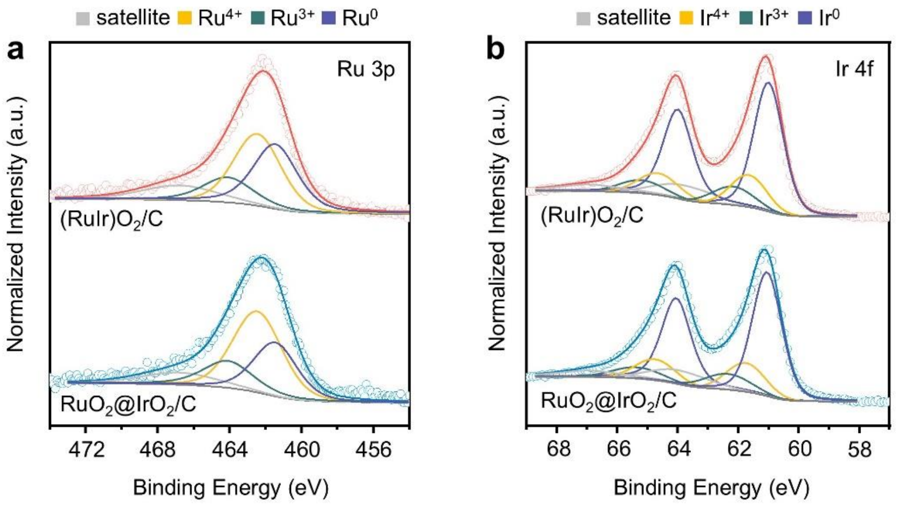

Supplementary Fig. 14 | XPS analysis. a Ru $3p_{3/2}$ and **b** Ir 4f XPS spectra of $(RuIr)O_2/C$ (top, red) and $RuO_2@IrO_2/C$ (bottom, blue).

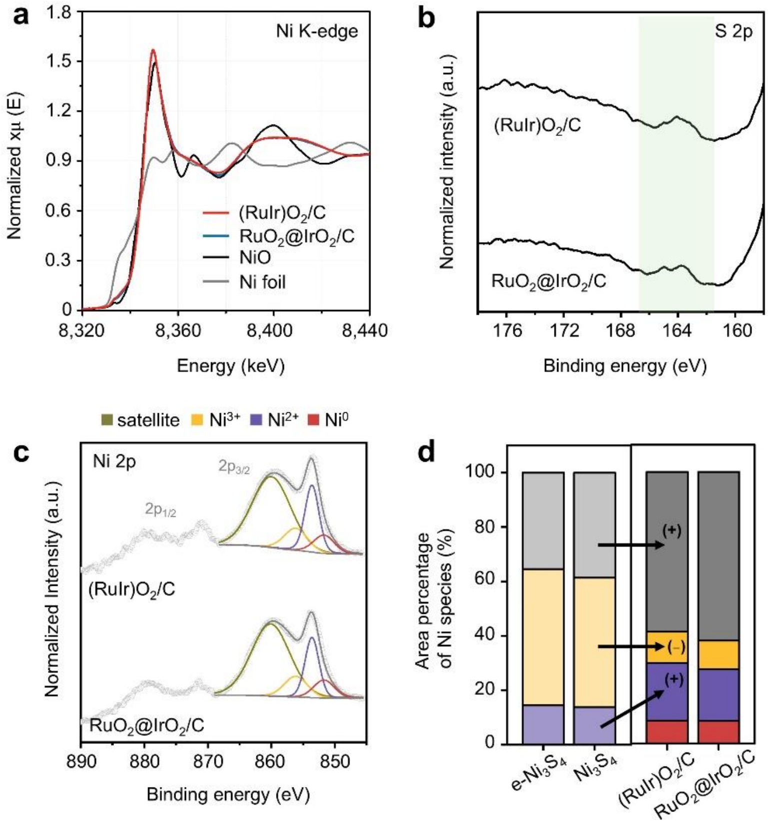

Supplementary Fig. 15 | Chemical state of Ni_3S_4-based templates within $(RuIr)O_2/C$ and $RuO_2@IrO_2/C$ after thermal oxidation. a Ni K-edge XANES spectra of $(RuIr)O_2/C$ and $RuO_2@IrO_2/C$ with NiO and Ni foil references. **b** S 2p and **c** Ni 2p XPS spectra of $(RuIr)O_2/C$ and $RuO_2@IrO_2/C$. **d** Area percentage of Ni species in Ni_3S_4-based templates (obtained from Supplementary Fig. 3) and $(RuIr)O_2/C$, $RuO_2@IrO_2/C$ (obtained from Supplementary Fig. 15). The satellite peaks indicate the presence of nickel-oxygen species[1-4].

Supplementary Note 3 | Chemical state of Ni_3S_4 and e-Ni_3S_4 templates during thermal oxidation.

Compared to the Ni 2p XPS results of the Ni_3S_4-based template (Supplementary Fig. 3), $(RuIr)O_2/C$ and $RuO_2@IrO_2/C$ exhibited a significant increase in the proportion of Ni^{2+} and satellite peaks, indicating the presence of nickel-oxygen species[1-4], while some Ni^{3+} species were still maintained. These results imply that the Ni_3S_4 phase in the core of $(RuIr)O_2/C$ and $RuO_2@IrO_2/C$ is partially oxidized to NiO and partially retained as Ni_3S_4 during the thermal oxidation process. Moreover, the S 2p XPS spectra also showed the presence of a small amount of S atoms even after thermal oxidation.

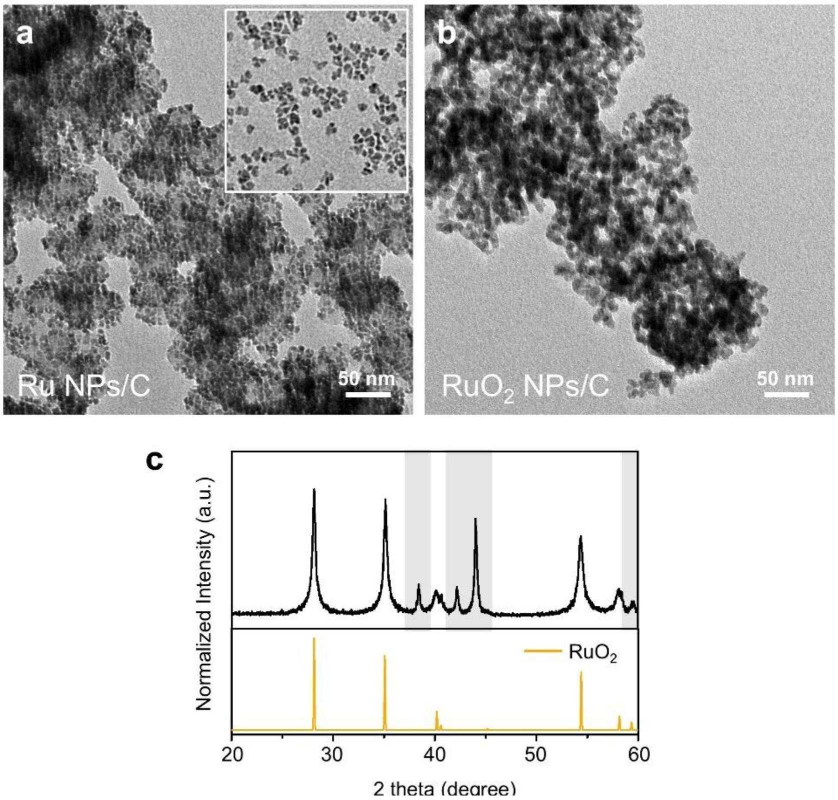

Supplementary Fig. 16 | Characterization of home-made RuO_2 NPs/C. TEM images of home-made **a** Ru nanoparticles (NPs)/C (inset: Ru NPs before carbon loading) and **b** RuO_2 NPs/C. **c** PXRD patterns of RuO_2 NPs/C. The gray box indicates the remaining metallic species in RuO_2 NPs/C.

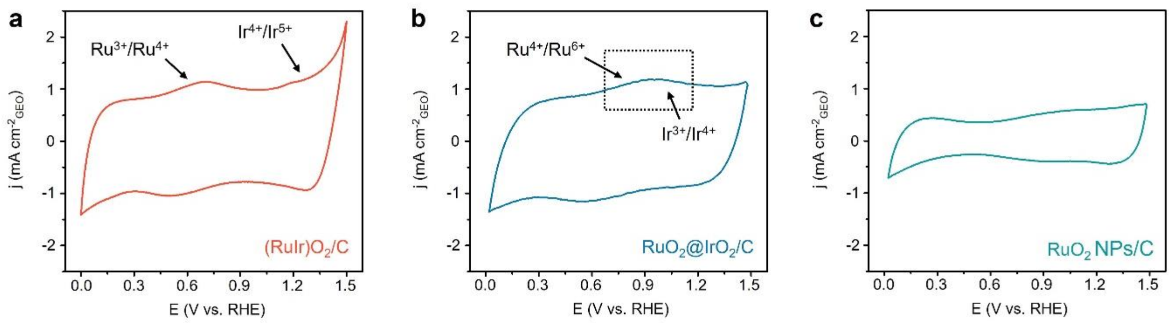

Supplementary Fig. 17 | Cyclic voltammetry (CV) curves. a $(RuIr)O_2/C$ **b** $RuO_2@IrO_2/C$, and **c** RuO_2 NPs/C in 0.1 M $HClO_4$ (pH = 1.02) at a scan rate of 20 mV s^{-1} within a potential range of 0.0−1.5 V_{RHE}.

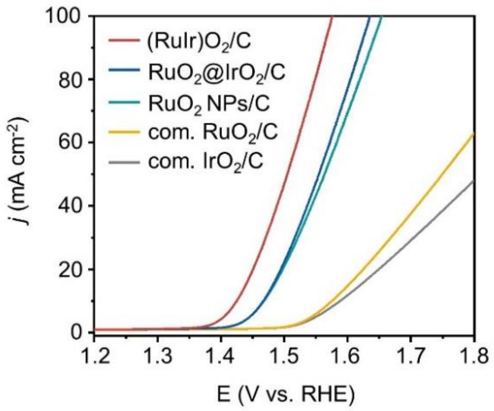

Supplementary Fig. 18 | OER activity without iR-compensation. LSV curves of $(RuIr)O_2$/C, RuO_2@IrO_2/C, RuO_2 NPs/C, com. RuO_2/C, and com. IrO_2/C without iR-compensation. The curves were measured in 0.1 M $HClO_4$ (pH = 1.02) at a scan rate of 5 mV s^{-1} and 1600 rpm. The noble metal loading was 50 g_{Ru+Ir} cm^{-2} for each electrocatalysts.

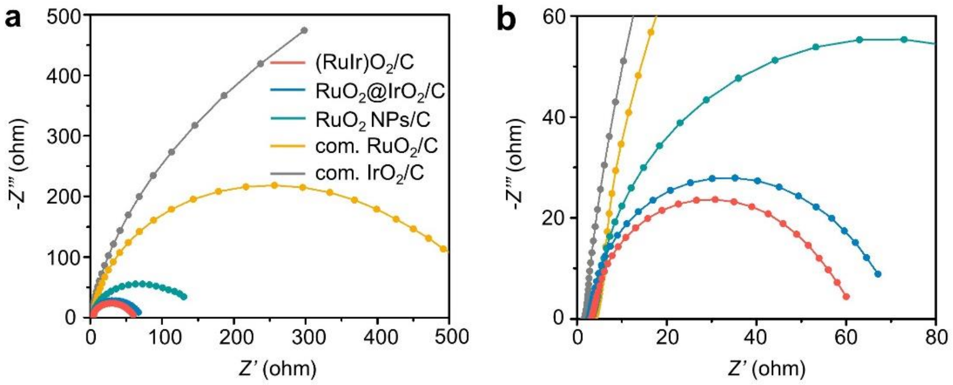

Supplementary Fig. 19 | Electrochemical impedance spectroscopy (EIS) analysis. a EIS and **b** enlarged EIS of different catalysts at 1.45 V_{RHE} in N_2-saturated 0.1 M $HClO_4$.

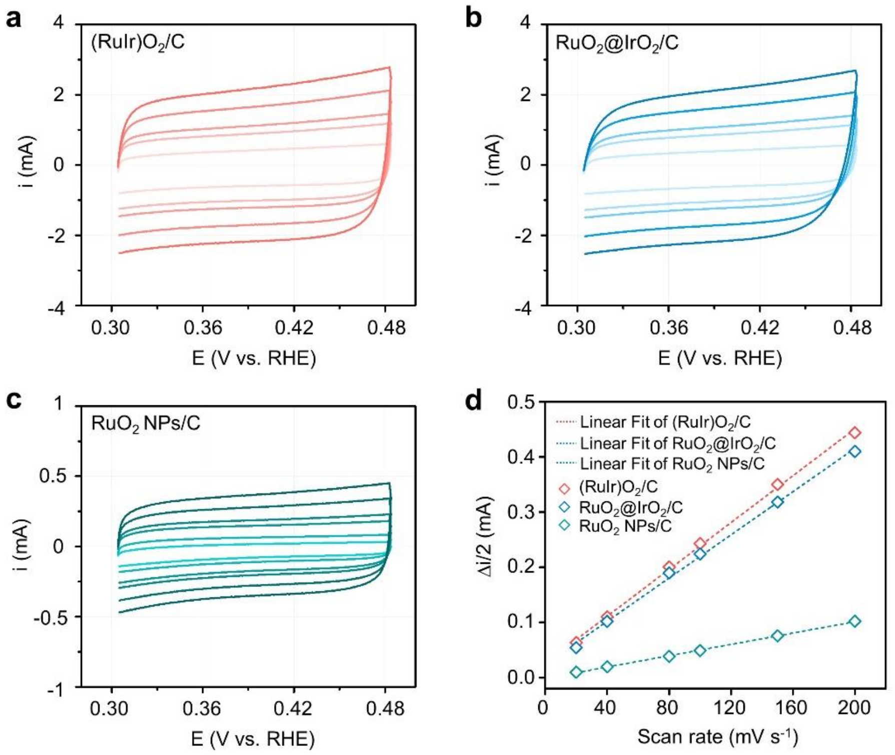

Supplementary Fig. 20 | Double layer capacitance (C_{dl}) at various scan rates. CV scans at different scan rates (20, 40, 80, 100, 150 and 200 mV s^{-1}) for **a** $(RuIr)O_2/C$, **b** $RuO_2@IrO_2/C$ and **c** RuO_2 NPs/C, respectively. **d** Linear plots of the half of the difference between anodic and cathodic charging currents measured at 0.39 V_{RHE} against scan rate. The ECSA values of $(RuIr)O_2/C$, $RuO_2@IrO_2/C$, and RuO_2 NPs/C obtained from C_{dl} were 60.6, 56.0, and 15.3 cm^2, respectively.

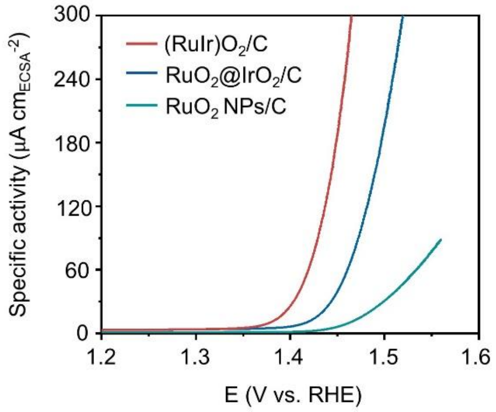

Supplementary Fig. 21 | OER specific activity curves of the catalysts. The specific activities normalized by the LSV curve (Fig. 4a) with the ECSA value.

Supplementary Note 4 | Structural characterization of unsupported Ru/Ir oxide-based electrocatalysts.

To eliminate the potential impact of carbon corrosion during the OER operation and to assess the effects of Ru/Ir atom configuration on OER performance, we synthesized unsupported RuO_2@IrO_2 and $(RuIr)O_2$ using SiO_2 as sacrificial substrate instead of carbon. In these experiments, nanoparticles were first supported on SiO_2, subjected to thermal oxidation, and subsequently, the SiO_2 was removed using hydrofluoric acid (Supplementary Fig. 22a). The RuO_2@IrO_2 and $(RuIr)O_2$ electrocatalysts were thus synthesized without a supporting substrate. During the removal of SiO_2, some particle aggregation was observed, indicating the role of the carbon supports in preventing such particle aggregation (Supplementary Fig. 22b). The PXRD analysis confirmed that both electrocatalysts successfully converted to their oxide phases during the thermal oxidation process, exhibiting the same behavior as when synthesized on carbon support (Supplementary Fig. 22c). Furthermore, the Ru and Ir atomic configurations in RuO_2@IrO_2 (Supplementary Fig. 23a, c-e) and $(RuIr)O_2$ (Supplementary Fig. 23b, f-h) retained their alloy and inner shell@outer shell structures, respectectively. This confirms that we successfully synthesized electrocatalysts with the same atomic configurations as those obtained using carbon supports.

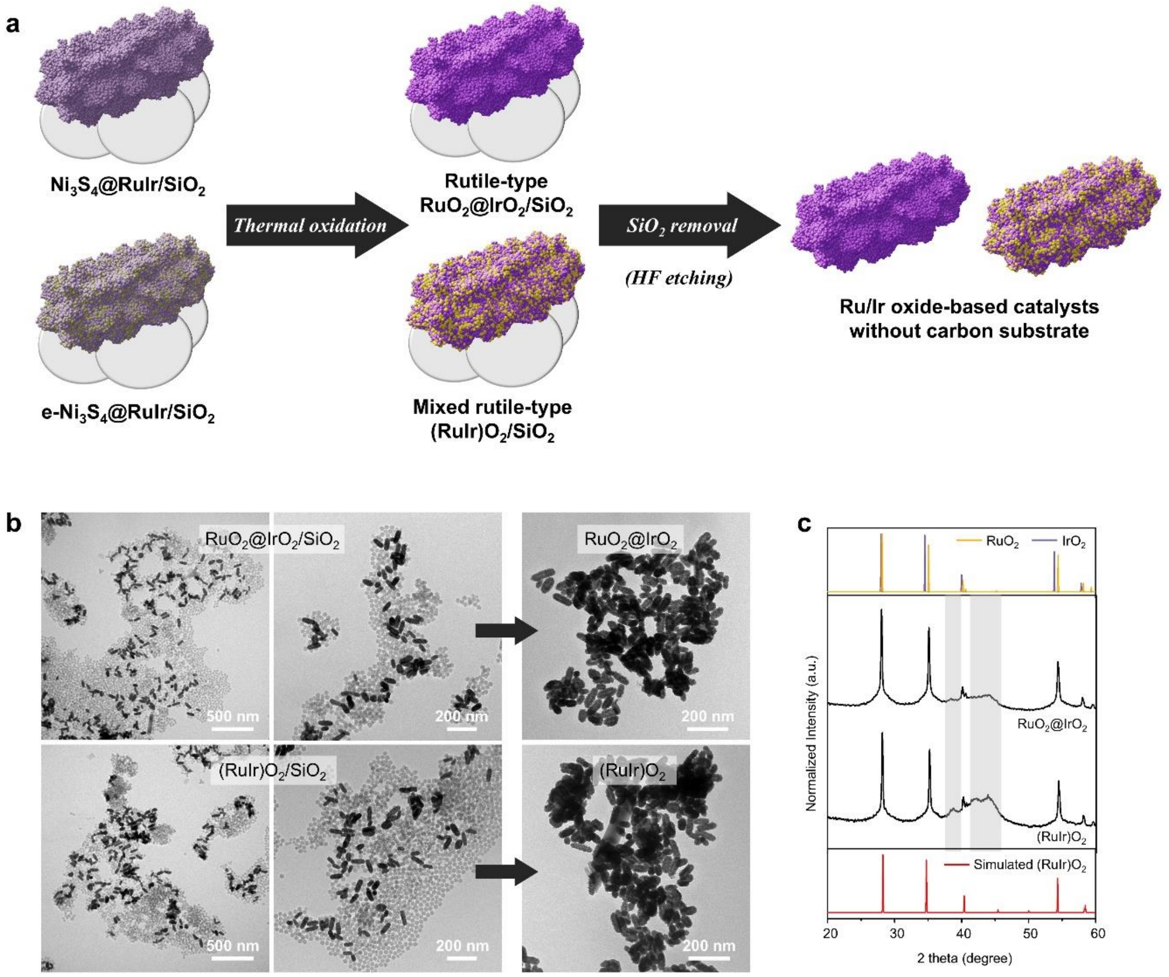

Supplementary Fig. 22 | Structural characterization of unsupported RuO_2@IrO_2 and $(RuIr)O_2$. a Schematic illustration and **b** TEM images for the formation of unsupported RuO_2@IrO_2 and $(RuIr)O_2$. **c** PXRD patterns for unsupported RuO_2@IrO_2 and $(RuIr)O_2$. Gray boxes denote the remaining metallic species in unsupported RuO_2@IrO_2 and $(RuIr)O_2$, which show the same behavior as with carbon support.

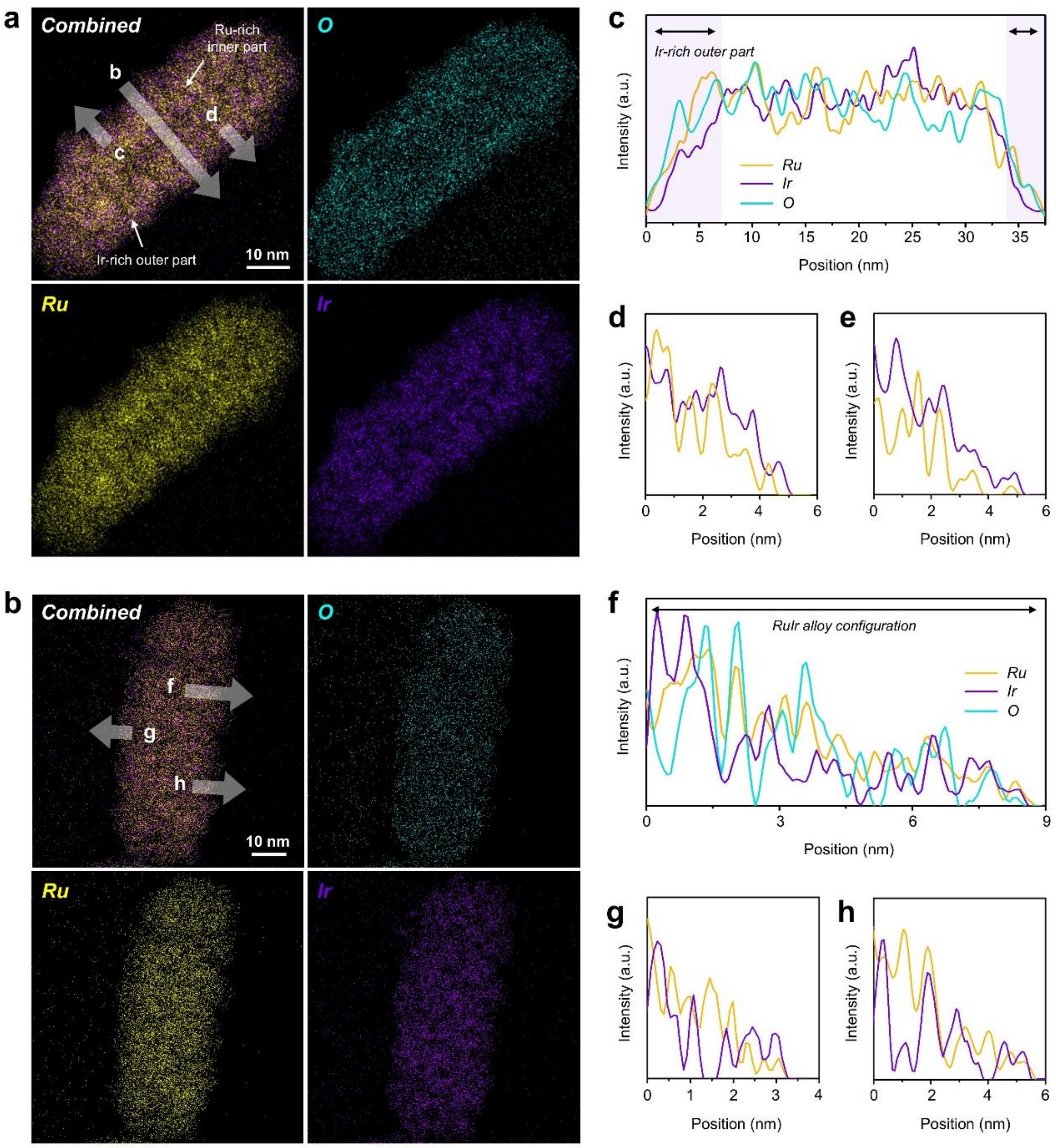

Supplementary Fig. 23 | Atomic composition analysis of unsupported RuO_2@IrO_2 and $(RuIr)O_2$. a Combined and individual EDS elemental mapping images of O (cyan), Ru (yellow), and Ir (purple) within unsupported **a** RuO_2@IrO_2 and **b** $(RuIr)O_2$. Line profile analysis for **c-e** RuO_2@IrO_2 and **f-h** $(RuIr)O_2$ corresponding to the marked area indicated by the white arrow in panel **a** and **b**, respectively.

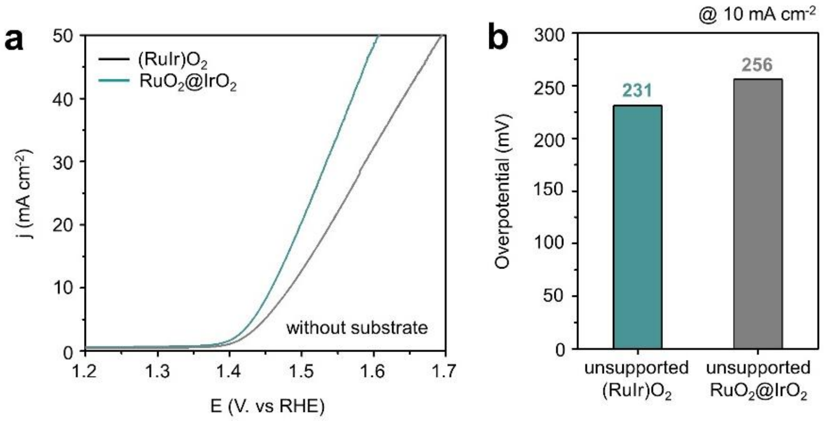

Supplementary Fig. 24 | OER activity of unsupported (RuIr)O_2 and RuO_2@IrO_2. a LSV curves of unsupported (RuIr)O_2 and RuO_2@IrO_2 in 0.1 M $HClO_4$ (pH = 1.02) at a scan rate of 5 mV s^{-1} and 1600 rpm. The noble metal loading was 50 μg_{Ru+Ir} cm^{-2} for each electrocatalysts. The measured potentials were 100% iR-comensated using the determined R_S value of 12 ± 0.3 Ω. **b** Overpotential of unsupported (RuIr)O_2 and RuO_2@IrO_2 to drive 10 mA cm^{-2} of current density. The overpotential values shown were measured once, and error bars were not included as no repeated measurements were performed.

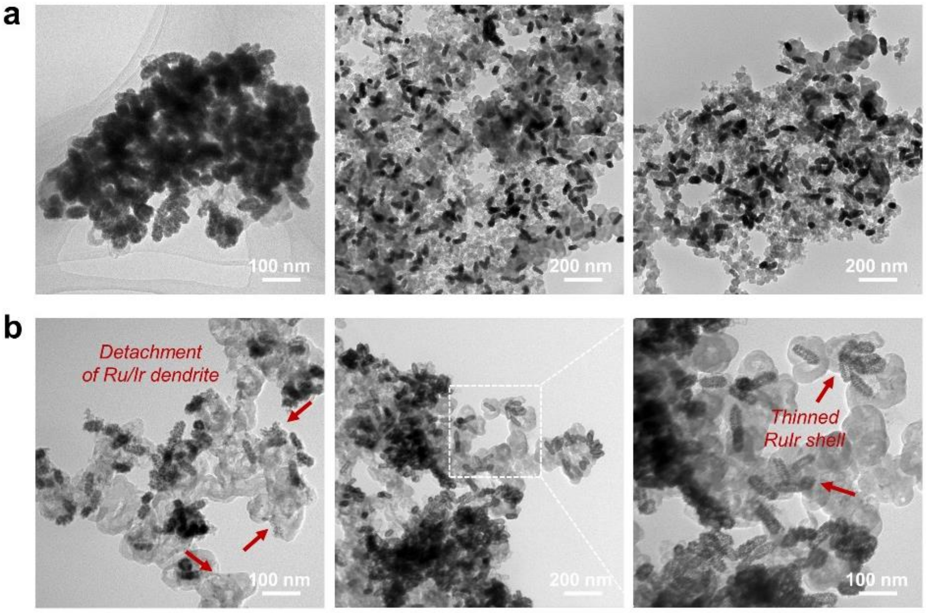

Supplementary Fig. 25 | TEM images after 24 h OER operation. a $(RuIr)O_2/C$ and **b** $RuO_2@IrO_2/C$.

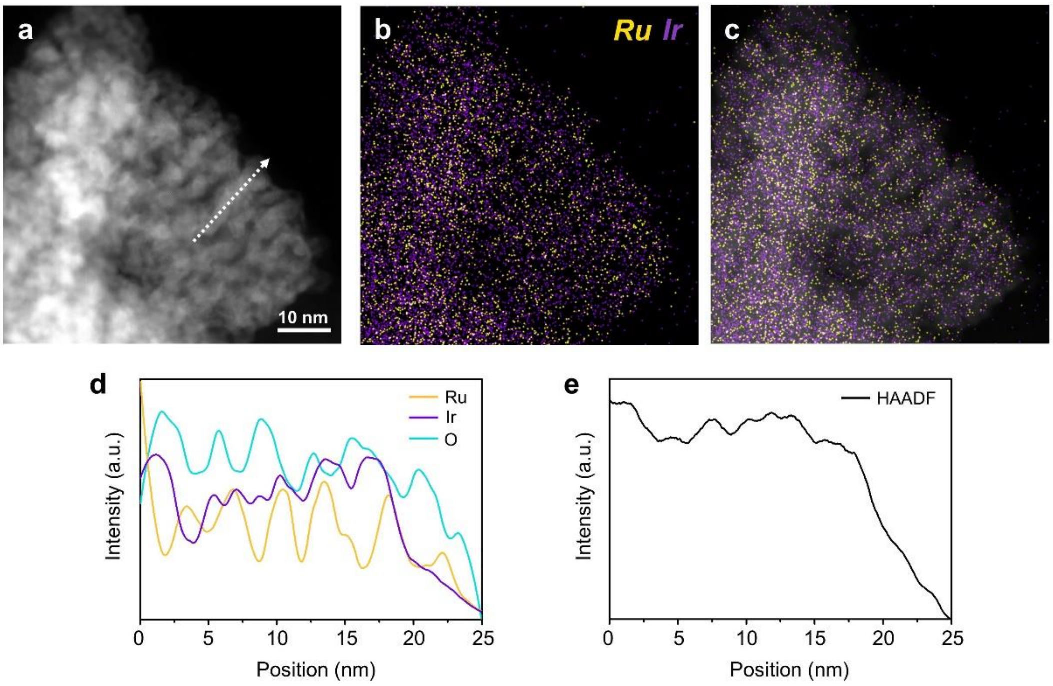

Supplementary Fig. 26 | Atomic composition analysis of (RuIr)O_2/C after 24 h OER operation. a HAADF-STEM and **b** elemental mapping images of Ru (yellow) and Ir (purple) contents in (RuIr)O_2/C after 24 h OER operation. **c** Combined images with HAADF-STEM and elemental mapping images. **d** Line profile analysis for Ru, Ir, and O contents and **e** HAADF profile corresponding to the marked area indicated by the white arrow in Supplementary Fig. 26a.

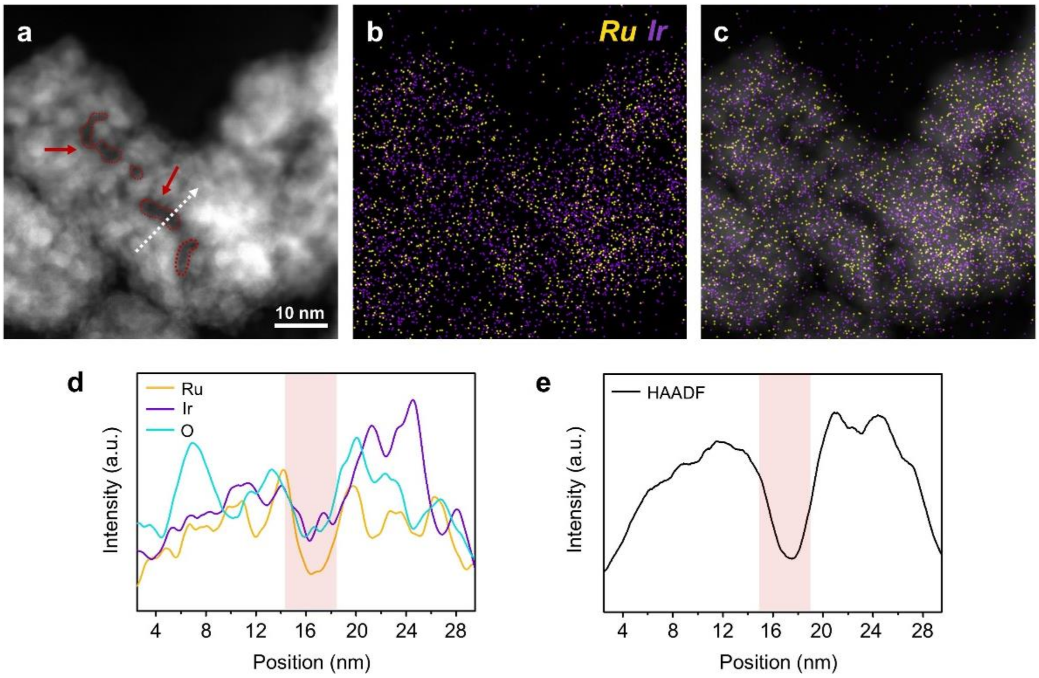

Supplementary Fig. 27 | Atomic composition analysis of RuO_2@IrO_2/C after 24 h OER operation. **a** HAADF-STEM and **b** elemental mapping images of Ru (yellow) and Ir (purple) contents in RuO_2@IrO_2/C after 24 h OER operation. **c** Combined images with HAADF-STEM and elemental mapping images. **d** Line profile analysis for Ru, Ir, and O contents and e HAADF profile corresponding to the marked area indicated by the white arrow in Supplementary Fig. 27a. The areas marked with red dashed lines in **a** and red box in **d**, **e** indicate the dissolution site of Ru and Ir atoms during the OER operation.

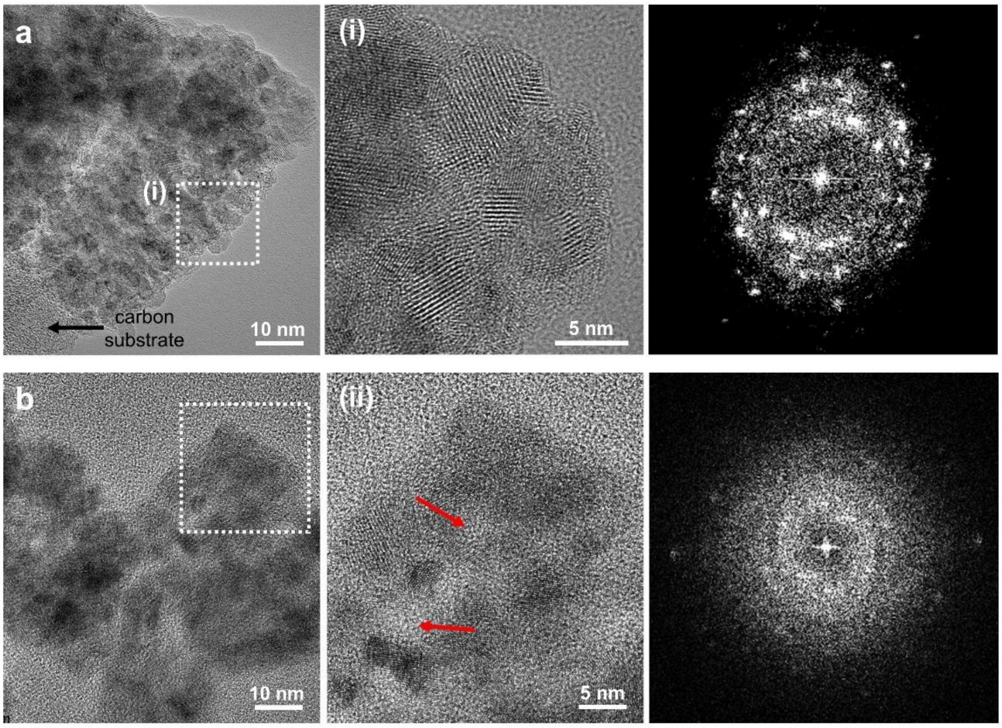

Supplementary Fig. 28 | Structural characterization after 24 h OER operation. HRTEM and enlarged HRTEM images with corresponding FFT pattern for **a** $(RuIr)O_2/C$ and **b** $RuO_2@IrO_2/C$ after 24 h OER operation. The red arrow indicates the dissolution site of Ru and Ir atoms during OER.

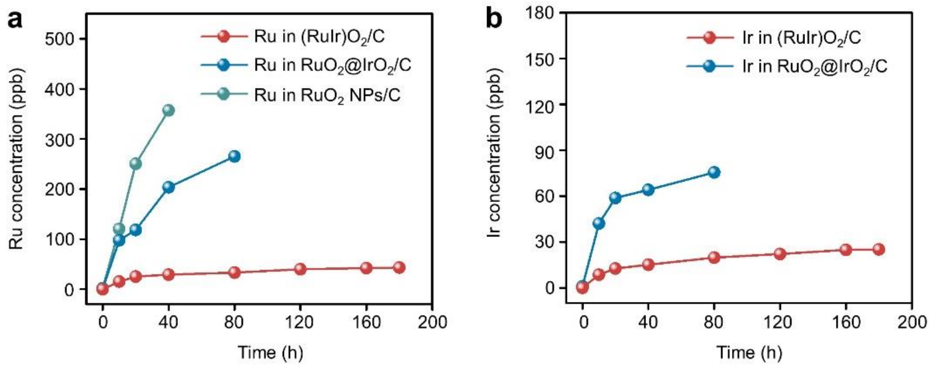

Supplementary Fig. 29 | ICP-MS analysis during OER operation. Leached out **a** Ru and **b** Ir concentrations in the electrolyte after CP test, determined through ICP-MS analysis.

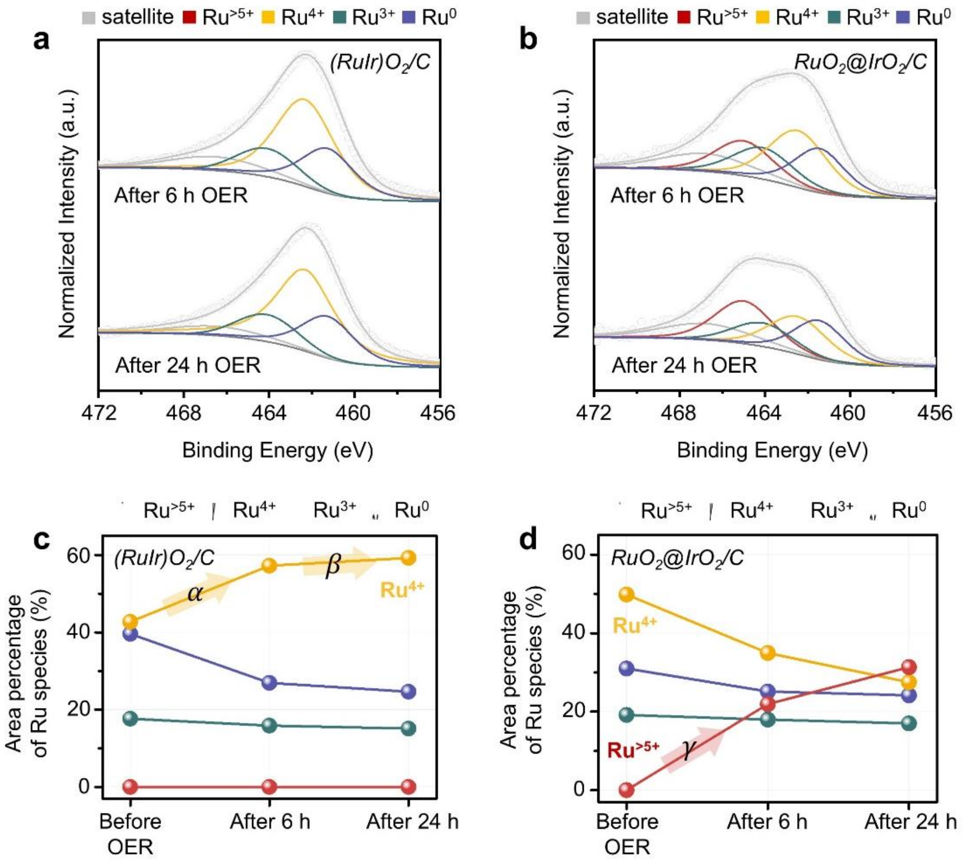

Supplementary Fig. 30 | Ru 3p XPS analysis after OER operation. Ru 3p$_{3/2}$ XPS spectra of **a** (RuIr)O_2/C and **b** RuO_2@IrO_2/C after 6 h and 24 h OER operation. Area percentage of Ru species in **c** (RuIr)O_2/C and **d** RuO_2@IrO_2/C based on the Ru 3p$_{3/2}$ XPS spectra of before (obtained from Supplementary Fig. 14a) and after 6 h, 24 h (obtained from Supplementary Fig. 30) OER. [α] Increasing robust Ru^{4+} and [β] maintaining Ru^{4+} species in (RuIr)O_2/C during OER operation. [γ] Increasing unstable $Ru^{>5+}$ species in RuO_2@IrO_2/C during OER operation.

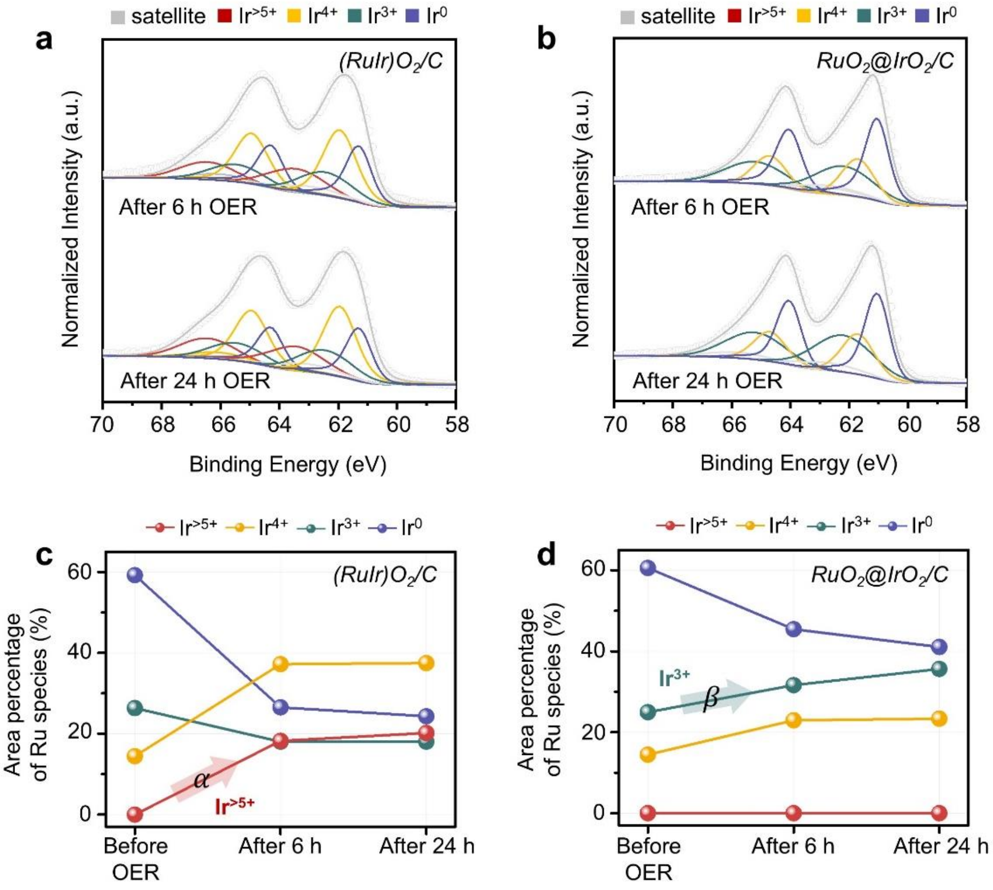

Supplementary Fig. 31 | Ir 4f XPS analysis after OER operation. Ir 4f XPS spectra of **a** $(RuIr)O_2/C$ and **b** $RuO_2@IrO_2/C$ after 6 h and 24 h OER operation. Area percentage of Ir species in **c** $(RuIr)O_2/C$ and **d** $RuO_2@IrO_2/C$ based on the Ir $3p_{3/2}$ XPS spectra of before (obtained from Supplementary Fig. 14b) and after 6 h, 24 h (obtained from Supplementary Fig. 31) OER. [α] Increasing OER-active $Ir^{>5+}$ species in $(RuIr)O_2/C$ during OER operation. [β] Increasing amorphous Ir^{3+} species aggregates in $RuO_2@IrO_2/C$ during OER operation.

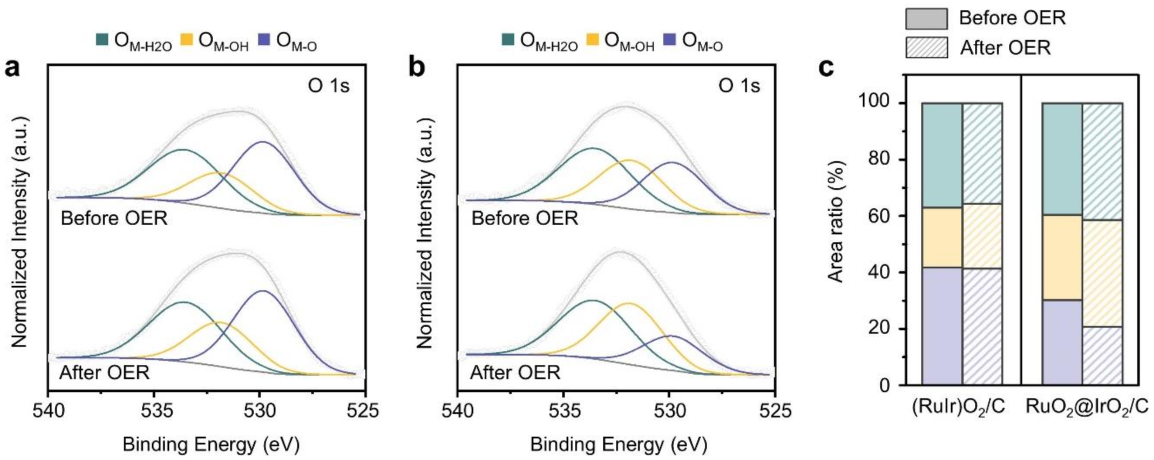

Supplementary Fig. 32 | XPS analysis after 24 h OER operation. O 1s deconvoluted XPS spectra of **a** (RuIr)O_2/C and **b** RuO_2@IrO_2/C before and after 24 h OER operation. **c** Area percentage of O species based on the O 1s XPS spectra of before and after 24 h OER operation.

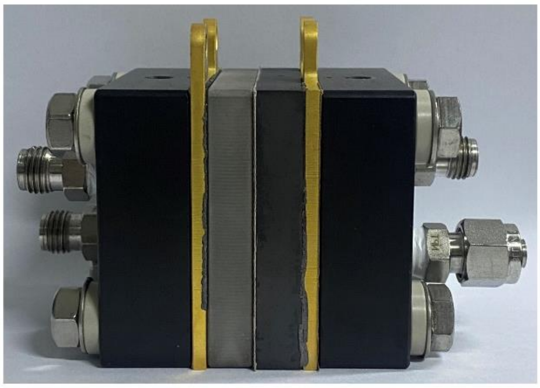

Supplementary Fig. 33 | Digital photograph of the MEA for PEMWE.

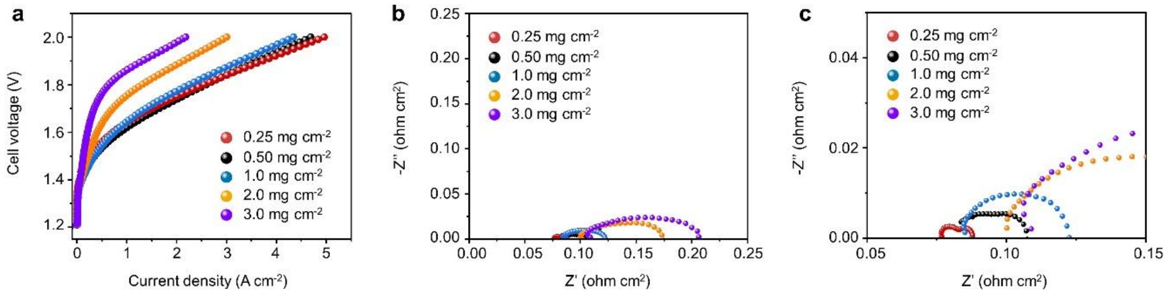

Supplementary Fig. 34 | Single-cell performances and EIS analysis in PEMWE. a Electrochemical single-cell performance for $(RuIr)O_2/C$ catalysts of different anode catalyst loading masses. **b** Nyquist plot and **c** enlarged Nyquist plot for $(RuIr)O_2/C$ catalysts of different anode catalyst loading mass at 2.0 V.

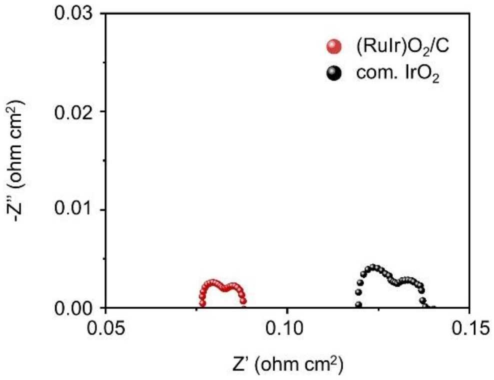

Supplementary Fig. 35 | EIS analysis in PEMWE. Nyquist plot of $(RuIr)O_2$/C and com. IrO_2 catalysts at 2.0 V.

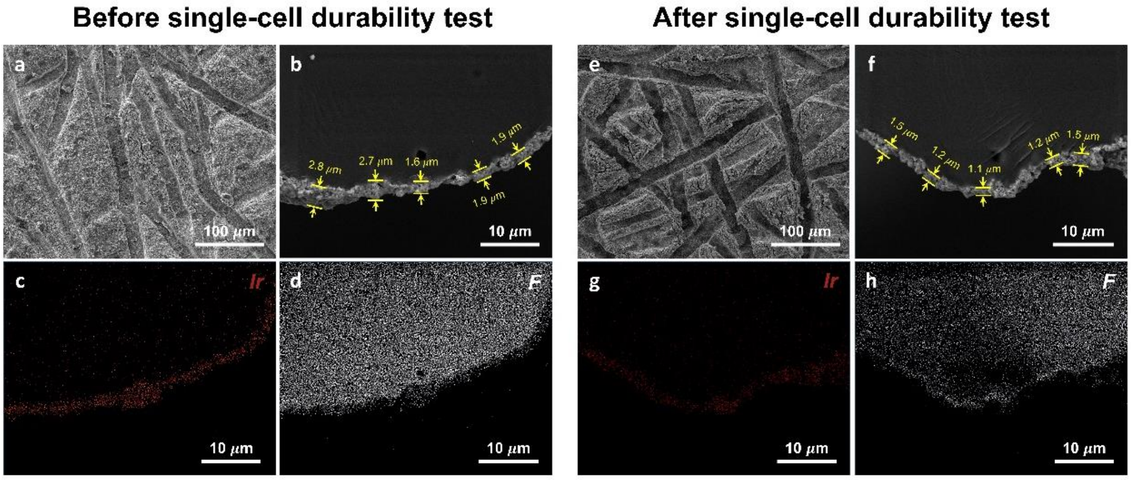

Supplementary Fig. 36 | Structural characterization of commercial IrO_2 electrocatalysts during single-cell test. SEM images for surface morphology **a** before and **e** after single-cell durability test. **b,f** Cross section images and corresponding elemental mapping images for **c,g** iridium (red), and **d,h** fluorine (gray) before and after single-cell durability test.

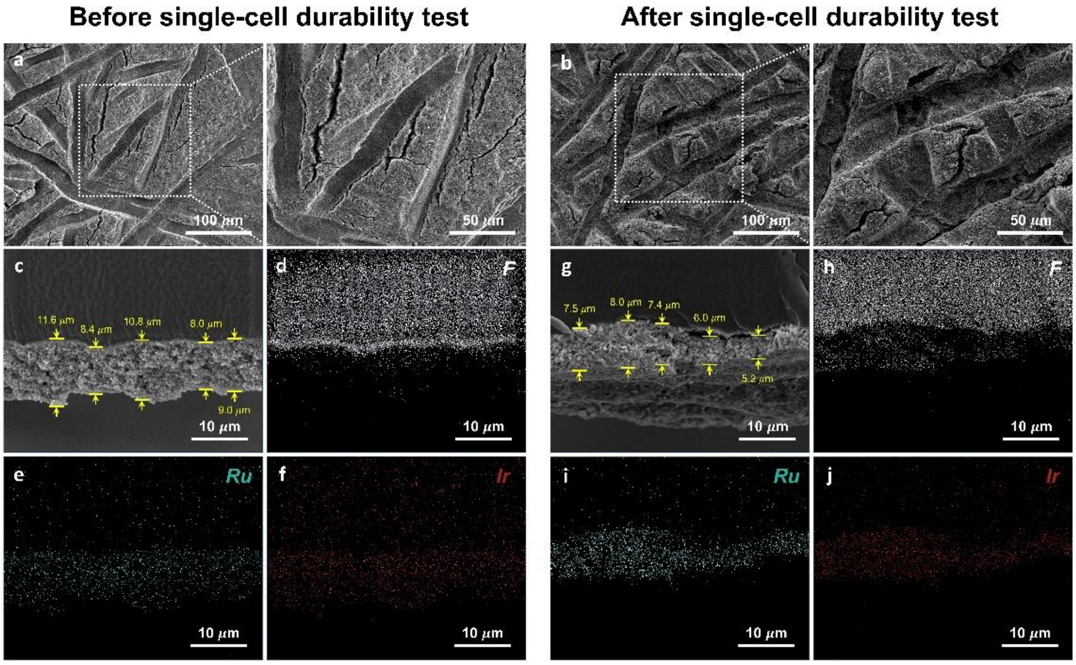

Supplementary Fig. 37 | Structural characterization of $(RuIr)O_2/C$ electrocatalysts during single-cell test. SEM images for surface morphology **a** before and **b** after single-cell durability test. **c,g** Cross section images and corresponding elemental mapping images for **d,h** fluorine (gray), **e,i** ruthenium (cyan), and **f,j** iridium (red) before and after single-cell durability test.

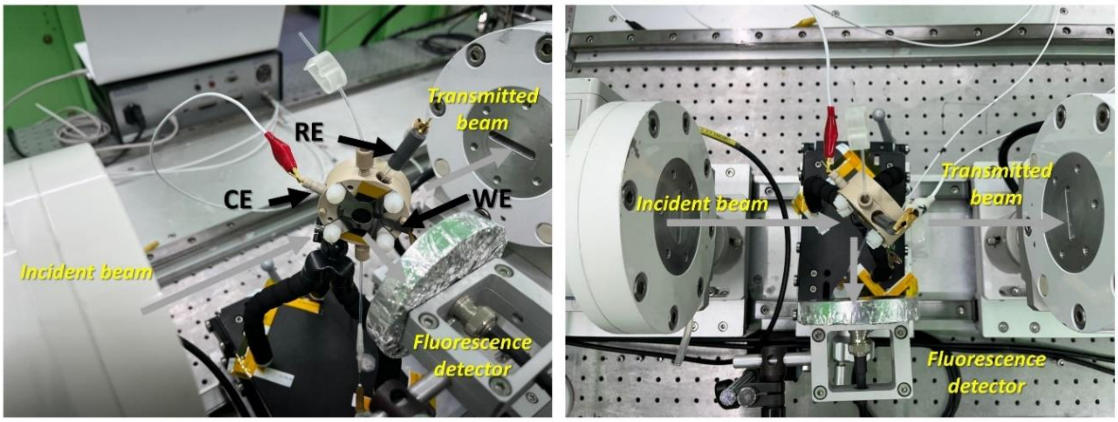

Supplementary Fig. 38 | Photo of an electrochemical cell for *in-situ* XAFS experiment setup. For the *in-situ* XAFS measurements, SPECTRO-electrochemical flow cell (C-A-MM_SPEC_EFC-10×10 mm^2, water-based electrolyte type cell, redoxme) was used.

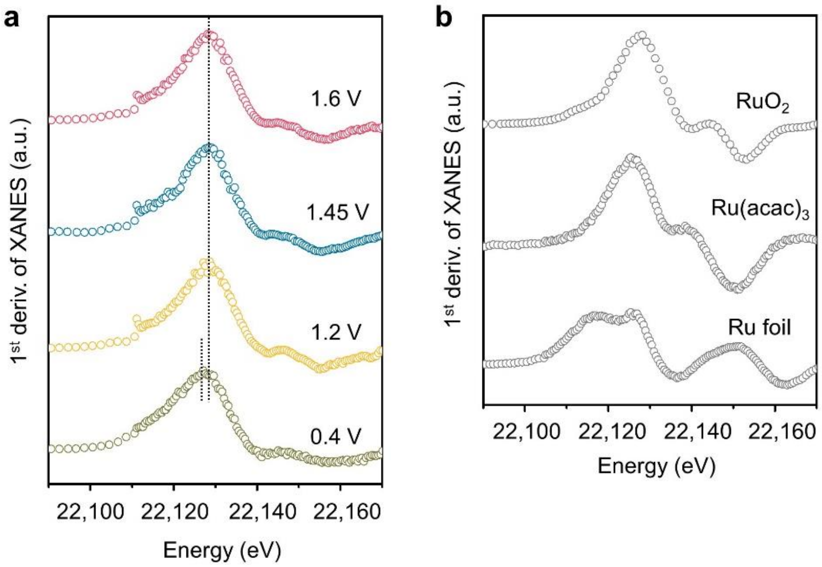

Supplementary Fig. 39 | First derivatives of Ru K-edge XANES regions. **a** (RuIr)O_2/C at different applied potentials during OER operation, and **b** control group samples: RuO_2, Ru(acac)$_3$, Ru foil.

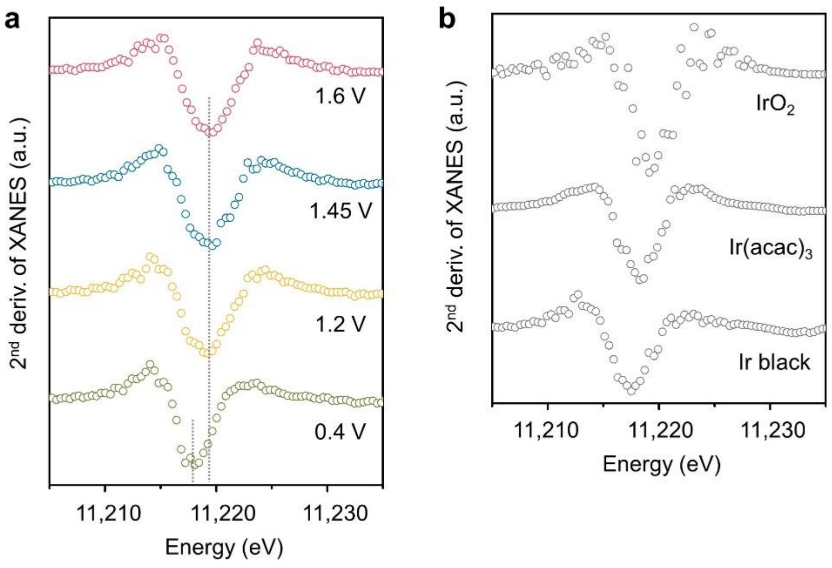

Supplementary Fig. 40 | Second derivatives of Ir L_3-edge XANES regions. a (RuIr)O_2/C at different applied potentials during OER operation, and **b** control group samples: IrO_2, Ir(acac)$_3$, Ir black.

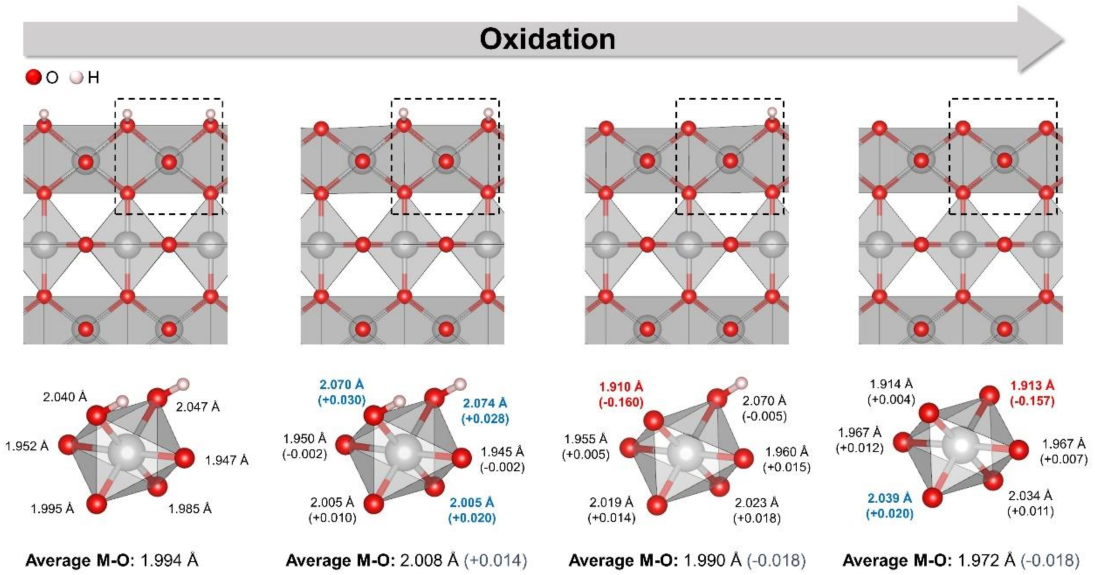

Supplementary Fig. 41 | Structural model for DFT calculations. The local structures of Ru in the M6c surface structure with the lowest energy shown as 32 in Supplementary Fig. 48. M−O bond length values are presented with the change in parenthesis, where blue and red bold fonts indicate an increase and decrease of 0.02 Å or more in M−O bond lengths, respectively.

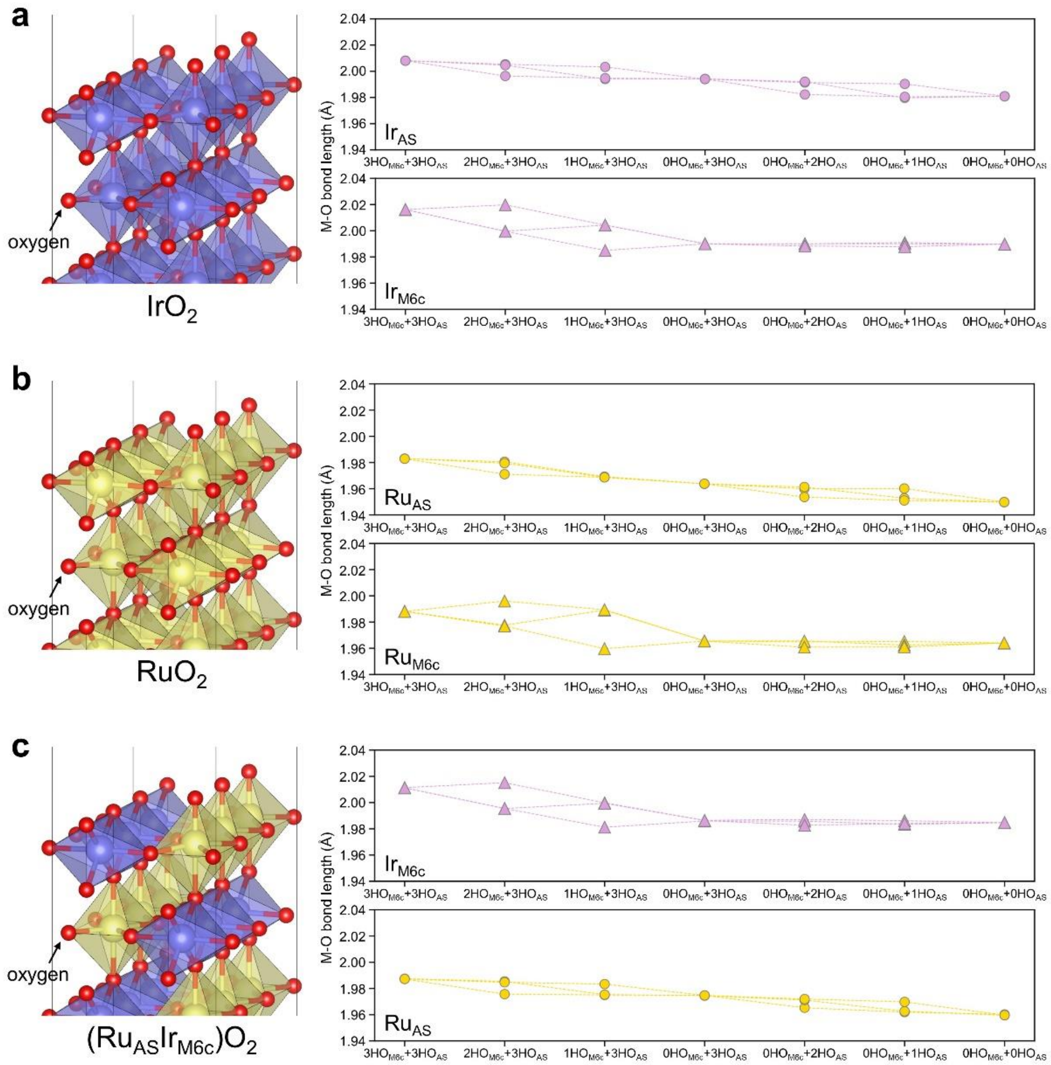

Supplementary Fig. 42 | DFT calculations for M-O bond lengths. a IrO_2, **b** RuO_2 and **c** $(Ru_{AS}Ir_{M6c})O_2$ according to the number of bonded H. X_{M6c} and X_{AS} correspond to X (X = Ru, Ir) in 6-coordinated metal sites and active sites, respectively.

Supplementary Note 5 | Computational Details

The spin-polarized density functional theory (DFT) calculations were performed using Vienna Ab Initio Simulation Package (VASP, version 5.4.4)[5,6] with the projector augmented wave (PAW) method [7,8]. The Perdew-Burke-Ernzerhof (PBE) exchange-correlation functional[9] with D3 correction scheme suggested by Grimme[10,11] was adopted. The kinetic cutoff energy and convergence tolerance of energy (force) were set to 500 eV and 10^{-4} eV (0.05 eV/Å), respectively. (7 × 7 × 10) and (4 × 5 × 1) Monkhorst-pack *k*-point meshes were sampled for bulk and surface structures, respectively[12]. The ideal gas (harmonic) approximation for gas molecules (adsorbates) as implemented in Atomic Simulation Environment (ASE) were used to convert electronic energies into Gibbs free energies, *i.e.*, $\Delta G = \Delta E + \Delta ZPE + \int C_p dT - T\Delta S$ $(T = 298\ K)$, where ΔE, ΔZPE, $\int C_p dT$ and $-T\Delta S$ correspond to changes in DFT energy, zero-point energy, enthalpic and entropic contributions, respectively[13]. The computational hydrogen electrode (CHE) method was used to construct the Gibbs free energy diagram, where the chemical potentials of proton and electron pair are set to be equivalent to half the chemical potential of H_2(g) *i.e.*, $\mu_{(H^+ + e^-)} = 0.5\mu_{H_2}$ at no applied potential[14]. The effect of the electrode potential (U_{RHE}) was included as $\mu_{(H^+ + e^-)} = 0.5\mu_{H_2} - eU_{RHE}$. We optimized the bulk rutile structures (space group: *P4₂/mmm*) of RuO_2 (a=4.514Å, c=3.109 Å) and IrO_2 (a=4.525 Å, c=3.180 Å), where the lattice parameters are in good agreement with the experimental results[15]. We then modeled three-layered (2 × 3) (110) surface structure. The bulk structure of $(RuIr)O_2$ was modeled by substituting Ru atoms with Ir atoms to achieve a Ru:Ir atomic ratio of 1:1. Subsequently, the mixed rutile structures were optimized, resulting in the lattice parameters of a = 4.513 Å and c = 3.151 Å. The unique surface structures of $(RuIr)O_2$ were identified by employing the pymatgen StructureMatcher module, which reduced the total number of configurations from 400 to 38[16]. For surface calculations, the bottommost layer of 3-layered surface models were constrained. The Bader charge analysis was performed to compare oxidation states of Ir and Ru atoms[17]. The Crystal Orbital Hamiltonian Population (COHP) and density of states (DOS) calculations using the tetrahedron method with Blöchl corrections were adopted to analyze the bonding properties[18,19]. The band center (ε) and ICOHP were calculated as follow:

$$\varepsilon = \frac{\int_{-\infty}^{+\infty} E \cdot \rho(E) \cdot dE}{\int_{-\infty}^{+\infty} \rho(E) \cdot dE}$$

$$ICOHP = \int_{-\infty}^{E_F} COHP \cdot dE$$

where E, ρ(E) and E_F correspond to energy, density of states and the Fermi level, respectively.

The reaction pathway of OER with four electron-proton transfer is assumed to be[20]

$H_2O + * \rightarrow OH* + (H^+ + e^-)$ (1)

$OH* + * \rightarrow O* + (H^+ + e^-)$ (2)

$O* + H_2O \rightarrow OOH* + (H^+ + e^-)$ (3)

$$OOH^* \rightarrow O_2 + (H^+ + e^-) \quad (4)$$

where * denotes an active site and *O, *OH, *OOH correspond to adsorbed species. The binding Gibbs free energies of each adsorbate were calculated as

$$\Delta G_{OH^*} = E(OH^*) + 0.5\mu(H_2) - E(^*) - \mu(H_2O) + G_{corr,\ OH^*}$$

$$\Delta G_{O^*} = E(O^*) + \mu(H_2) - E(^*) - \mu(H_2O) + G_{corr,\ O^*}$$

$$\Delta G_{OOH^*} = E(OOH^*) + 1.5\mu(H_2) - E(^*) - 2\mu(H_2O) + G_{corr,\ OOH^*}$$

where E, μ, and G_{corr} are DFT energy, the chemical potential of gaseous molecules and the Gibbs free energy correction values for adsorbates, respectively. The OER overpotential (η) was then calculated as $MAX(\Delta G_{OH^*}, \Delta G_{O^*} - \Delta G_{OH^*}, \Delta G_{OOH^*} - \Delta G_{O^*}, 4.92 - \Delta G_{OH^*})/e - 1.23$ V.

We note that all properties presented in this work (i.e., ΔG, oxidation states, band centers, -ICOHP, M-O bond lengths) of $(RuIr)O_2$ were calculated considering the Boltzmann probability based on the relative energetics of bare surfaces[21]:

$$\varphi = \frac{\sum_{i=1}^{N} \varphi_i \times \exp\{-E_{DFT,i}/k_B T\}}{\sum_{i=1}^{N} \exp\{-E_{DFT,i}/k_B T\}}$$

where φ_i and $E_{DFT,i}$ correspond to the properties and DFT energy of i-th $(RuIr)O_2$ surface structure. The k_B, T and N are the Boltzmann constant, temperature (298.15 K) and the total number of surface structures, respectively.

The interface between spinel Ni_3S_4 core and metal shells were modeled by replacing the top layer of three-layered Ni_3S_4 (001) surface with metal atoms. The unit cell parameters of the Ni_3S_4 (001) surface were obtained by optimizing the cubic spinel bulk structure (a=9.366 Å). Additionally, the unit cell parameters of the Ni_3S_4 (001) surface, expanded due to Ir doping, were adjusted by increasing the lattice parameters of the pristine Ni_3S_4 (001) surface by 6 % in both the x and y directions (Supplementary Fig. 2). Utilizing these pristine and expanded Ni_3S_4 (001) models with metal-substituted top layers, Ab Initio Molecular Dynamics (AIMD) simulations were conducted in an NVT ensemble using a Nosé–Hoover thermostat[22]. The optimized Ni_3S_4-metal interface structures were equilibrated for 10 ps with 1 fs time step, and trajectories spanning 5 ps were subsequently collected. The CIF files of initial and final structure of AIMD simulations were uploaded in Supplementary Data 1.

Supplementary Note 6 | Site-dependent oxidation states of Ir.

To model the most realistic surface structures of $(RuIr)O_2$, we performed the Bader charge analysis based on the locations of Ir atoms. We identified the Ir site with the highest oxidation state for up to three Ir atoms. For a single Ir atom in the system, we considered various sites, including active sites (AS), six-coordinated metal sites (M_{6c}), sub-positions of active sites (sub-AS), and sub-positions of six-coordinated metal sites (sub-M_{6c}). The Ir atom at the AS demonstrated the highest oxidation state. Using this structure as a reference, we compared the average oxidation state of Ir atoms when the second Ir atom was placed at all possible sites. The average oxidation state of Ir atoms was also highest when the second Ir atom was located in the AS. Finally, when two Ir atoms occupied the AS, the average oxidation state of Ir atoms was highest when the third Ir atom was also in the AS (Supplementary Fig. 43). This result indicates that the Ir position at the AS is capable of describing the highest oxidation state of Ir observed in Fig 6.

The origin of this result can be understood through the Bader charge values of the oxygen atoms in the top layer. There are five types of oxygen in the top layer, labeled O-1 through O-5 (Supplementary Fig. 44). The absolute Bader charge values for O-3 (-0.86), O-4 (-0.89), and O-5 (-0.88) are negative, indicating that these oxygen atoms withdraw a significant amount of electrons from their neighbors. To determine the charge withdrawn by each oxygen atom from a single metal atom, we normalized the absolute Bader charge of the oxygen by the number of its M-O bonds. We found that O-1, which is bonded to the AS, has the most negative normalized Bader charge value (-0.41), indicating a strong tendency to oxidize. This suggests that the metal atom located in the AS is the most oxidized. Therefore, we used the atomic structure with Ir occupying the AS throughout this work.

We observed that the oxidation state of Ir increases more significantly than that of Ru as the potential increases (Fig. 6). Previous studies have reported that the rutile surface undergoes a transition from OH* to O* termination upon the application of the oxidation potential[23]. Thus, we constructed all possible unique 6 structures satisfying Ru:Ir ratio of 1:1 in the top layer (Supplementary Fig. 45a) and calculated the Bader charges of metal atoms for both OH* and O* surface terminations. In the OH* termination, Ir and Ru in the top layer had similar Bader charge values (Suppleentary Fig. 45b). However, in the O* termination, Ru exhibited a higher a Bader charge except for the case of $3Ir_{AS}$. Notably, during the transition from OH* to O* termination, Ir showed a greater increase in Bader charge compared to Ru only in the 3 Ir_{AS} structure (Supplementary Fig. 46). This indicates that $3Ir_{AS}$ is the only case that matches the in situ XANES results, showing a greater increase in the oxidation state of Ir compared to Ru (Fig. 6). The structural files used in this analysis have been uploaded as Supplementary Data 1.

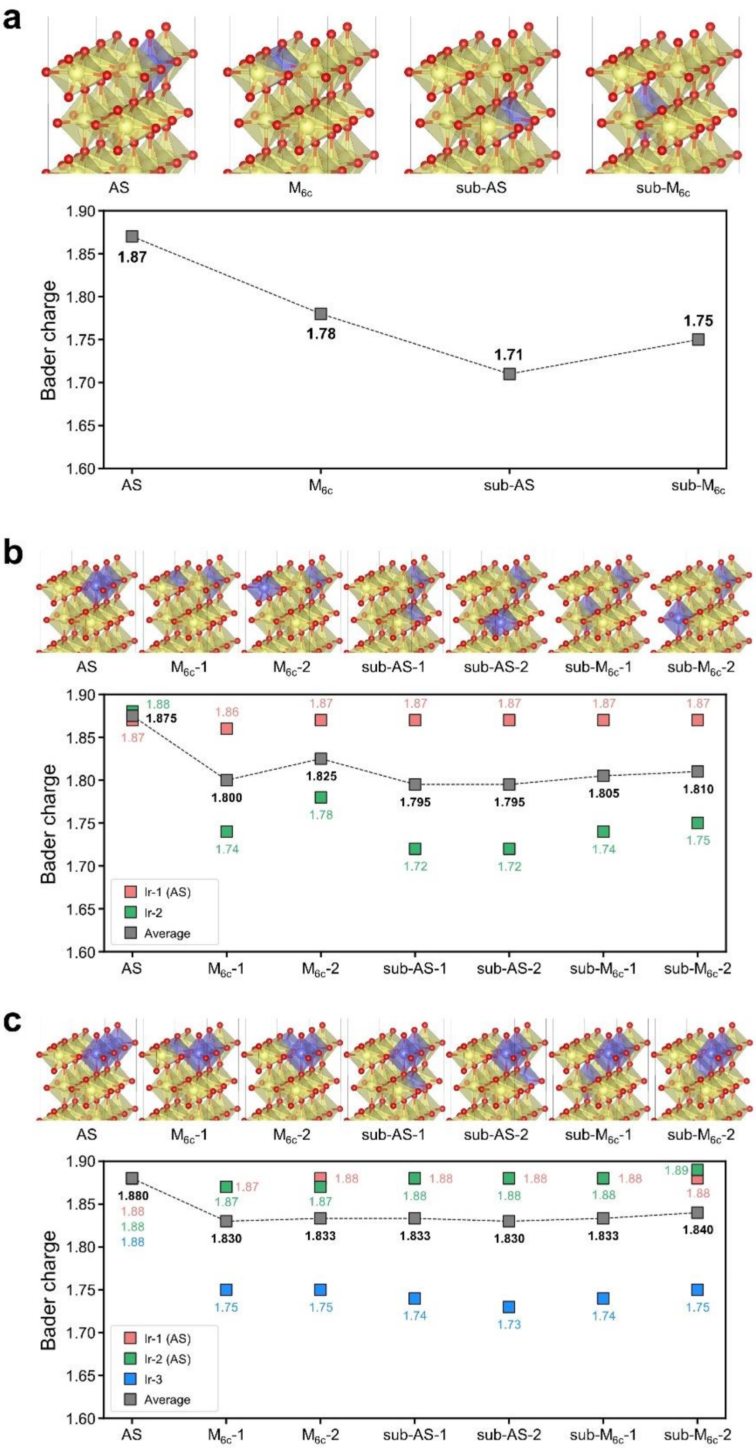

Supplementary Fig. 43 | DFT calculations for Bader charges. The Bader charge values of Ir atoms are shown according to their locations in the top layer when the number of Ir atoms is **a** one, **b** two, and **c** three. Ir atoms specified as '(AS)' refer to Ir fixed in the AS position. In the atomic structures, the red balls and yellow and purple polyhedrons represent O, Ru, and Ir atoms, respectively.

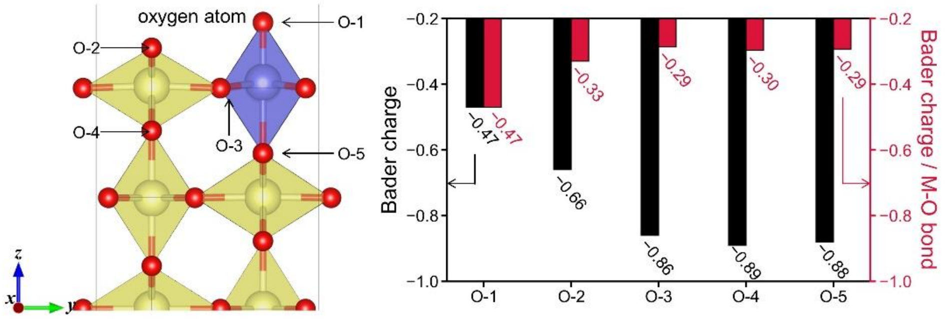

Supplementary Fig. 44 | Bader charge analysis of oxygen atoms in the top layer. The absolute and normalized Bader charge values of each oxygen atom in the top layer. The normalized values are based on the number of M-O bonds for each metal atom. O-1 and O-5 are bonded in the +z and -z directions relative to the AS position, respectively, while O-2 and O-4 are bonded in the +z and -z directions relative to the M_{6c} position, respectively. O-3 is located between AS and M_{6c} positions, bonded to both. O-1 has one M-O bond, O-2 has two M-O bonds, and O-3, O-4, and O-5 each have three M-O bonds. The red balls and yellow and purple polyhedrons in the atomic structures correspond to O, Ru, and Ir atoms, respectively.

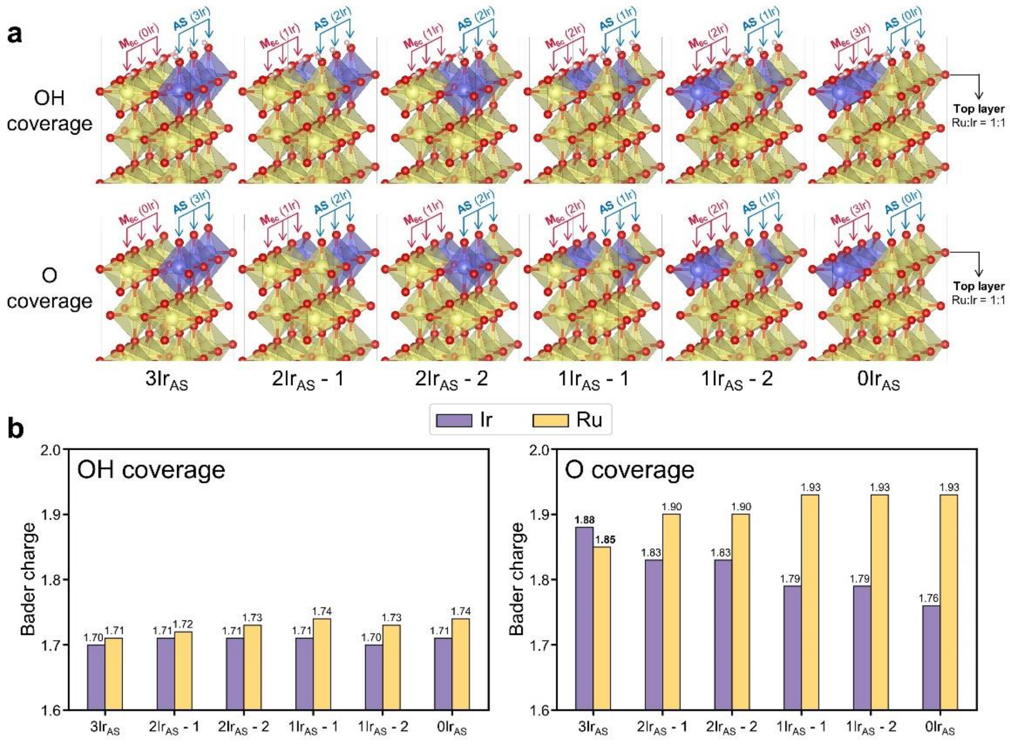

Supplementary Fig. 45 | Structural and charge analysis of Ru-Ir systems with OH* and O* terminations. a All possible unique structures satisfying Ru:Ir ratio of 1:1 in the top layer with OH* and O* termination. The names of the structures are designated based on the number of Ir atoms in the AS position **b** The Bader charge values of Ir and Ru for the OH* and O* terminations of the structures represented in **a**. The red and white balls and yellow and purple polyhedrons in the atomic structures correspond to O, H, Ru, and Ir atoms, respectively.

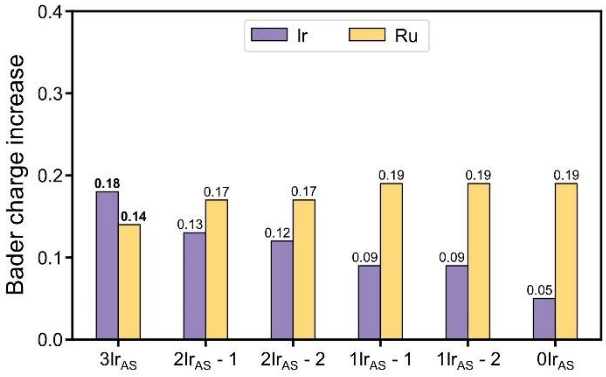

Supplementary Fig. 46 | Badder charge changes in Ir and Ru from OH* to O* termination.

The increase in Bader charge of Ir and Ru during the transition from OH* to O* termination.

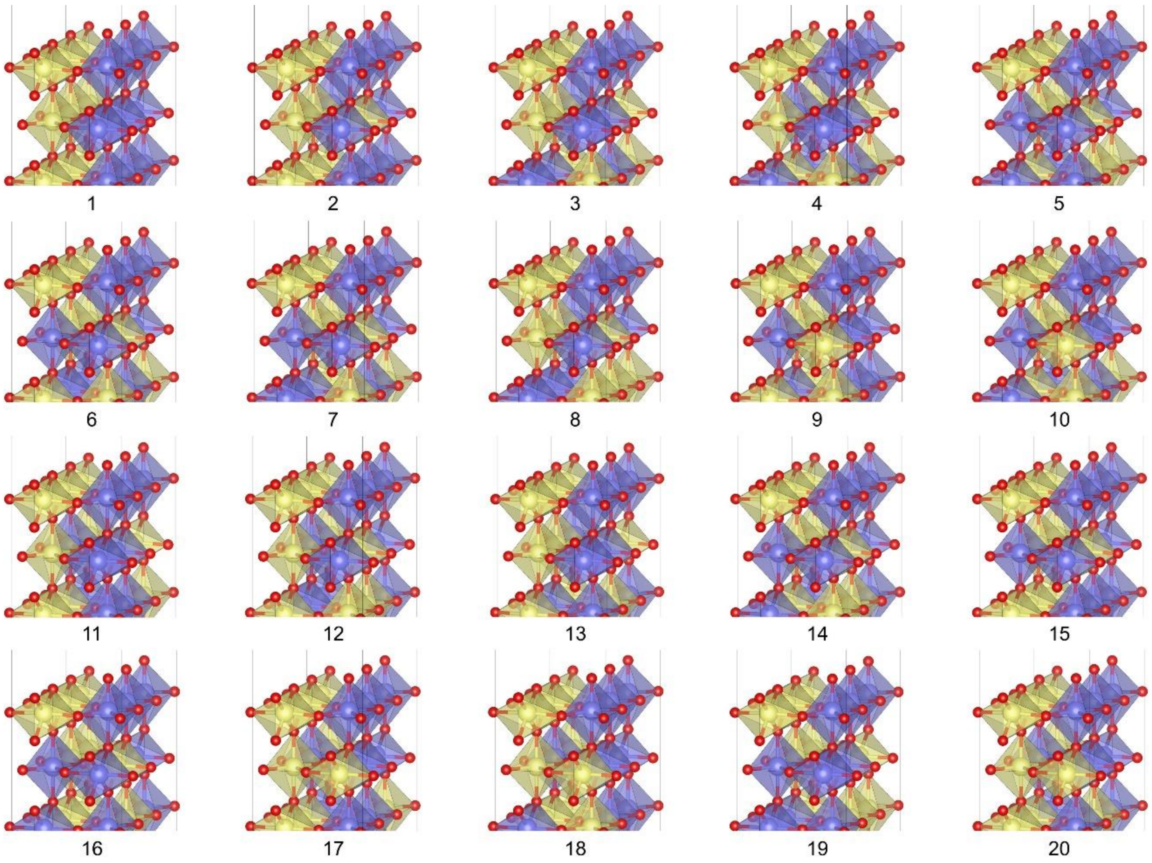

Supplementary Fig. 47 | All possible (RuIr)O_2 surface structure configurations (1-20). The red ball, yellow and purple polyhedrons in the atomic structures correspond to O, Ru, and Ir atoms, respectively.

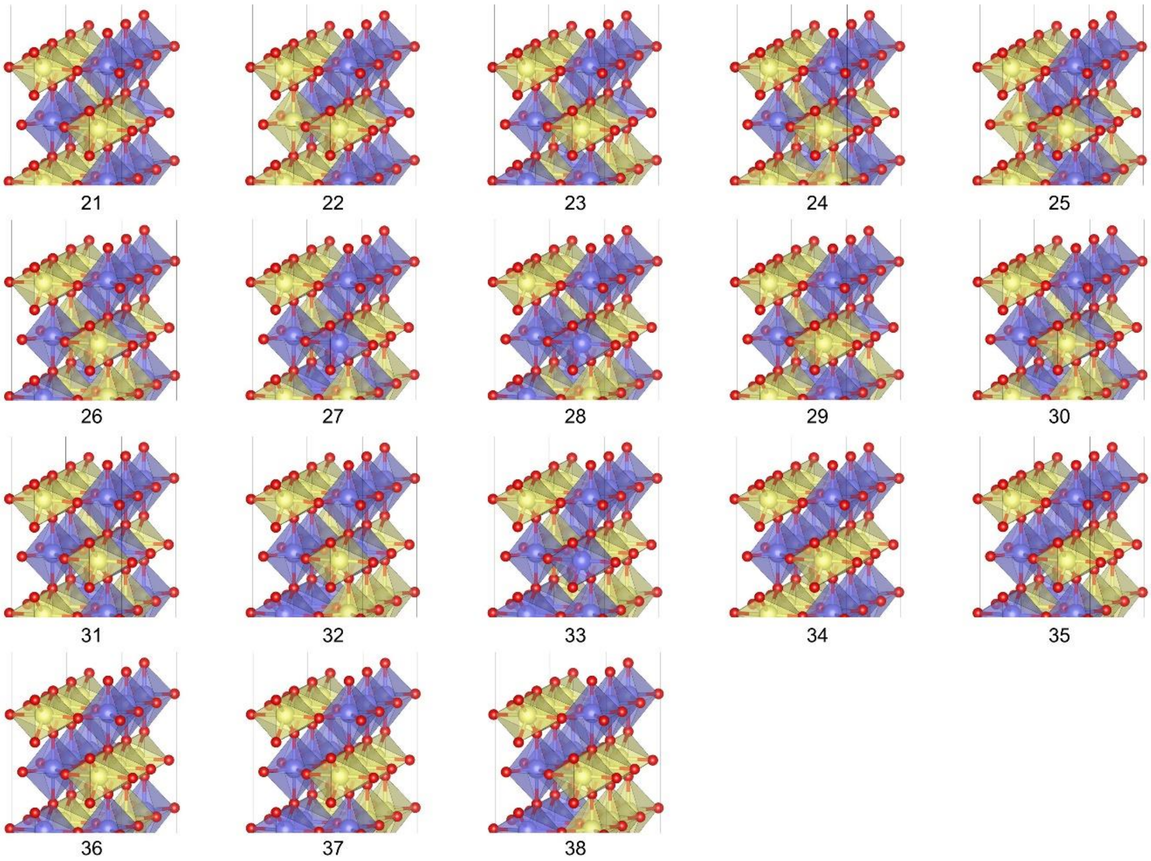

Supplementary Fig. 48 | All possible (RuIr)O_2 surface structure configurations (21-38). The red ball, yellow and purple polyhedrons in the atomic structures correspond to O, Ru, and Ir atoms, respectively.

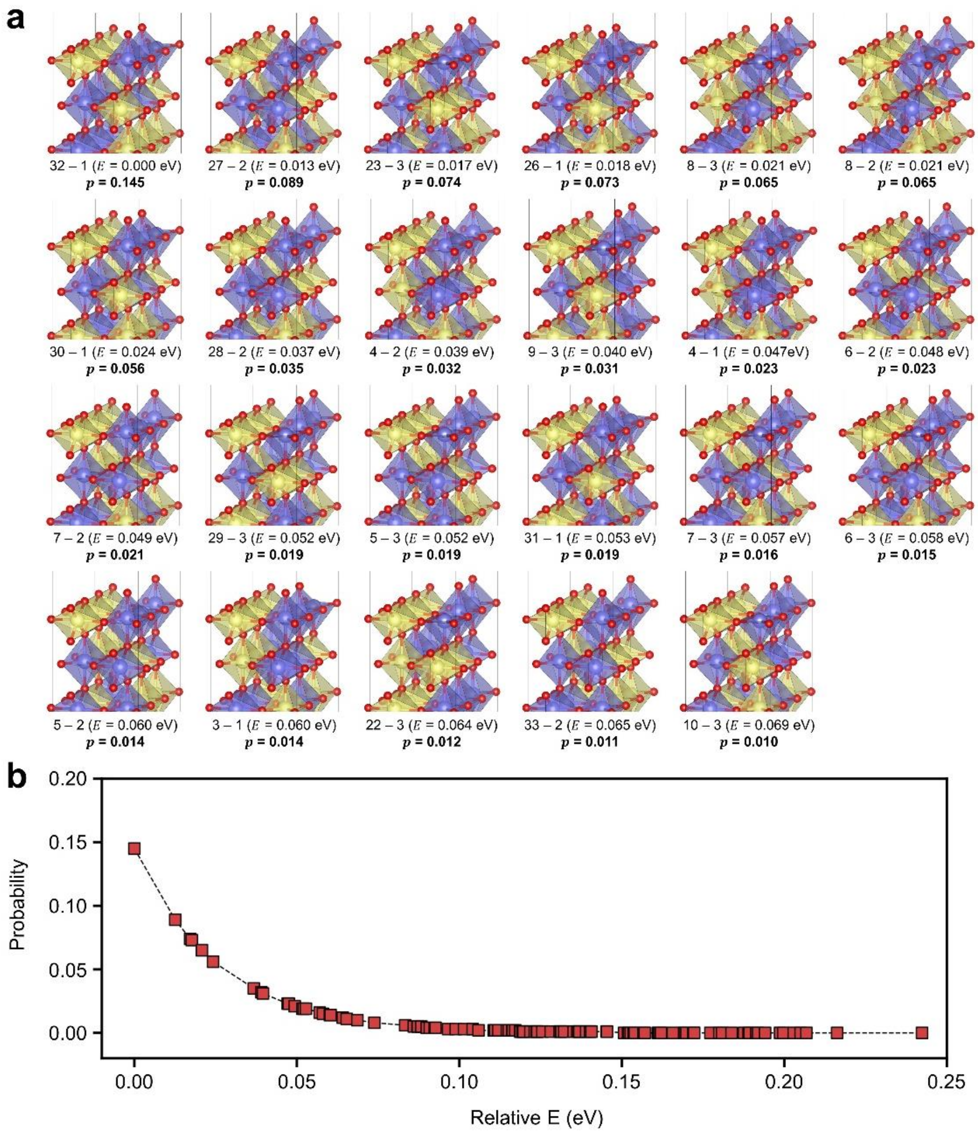

Supplementary Fig. 49 | DFT calculations. a The surface structures (structure name – active site index) with p_i > 0.01, relative energies with respect to the most stable structure, and probabilities calculated as ($\boldsymbol{p_i = \frac{\exp\{-E_{DFT,i}/k_B T\}}{\sum_{i=1}^{N} \exp\{-E_{DFT,i}/k_B T\}}}$). The red ball, yellow and purple polyhedrons in the atomic structures correspond to O, Ru, and Ir atoms, respectively. **b** The probabilities of surface structures according to the relative energies.

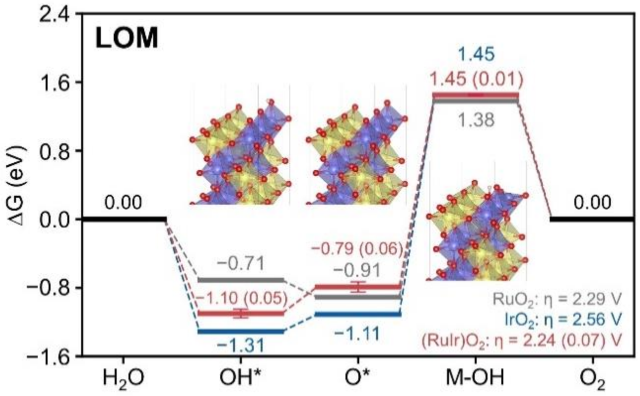

Supplementary Fig. 50 | Gibbs free energy diagram for the LOM pathway. For the values of $(RuIr)O_2$, the standard deviation is indicated in parentheses. The red and white balls and yellow and purple polyhedrons in the atomic structures correspond to O, H, Ru and Ir atoms, respectively.

Supplementary Note 7 | OER mechanism analysis through the LOM pathway.

The OER mechanism of RuO_2, IrO_2, and $(RuIr)O_2$ through LOM pathway were also analyzed (Supplementary Fig. 50). For the $(RuIr)O_2$ surface, we considered the surface structures with a probability great than 0.05 (Supplementary Fig. 48), and averaged their LOM overpotentials. The following step reactions were considered as LOM[24]:

$$H_2O + * \rightarrow OH* + (H^+ + e^-)$$

$$OH* \rightarrow O* + (H^+ + e^-)$$

$$O* + M\text{-}O \rightarrow O_2 + (H^+ + e^-)$$

$$M\text{-}(vac) + H_2O \rightarrow M\text{-}OH + (H^+ + e^-)$$

$$M\text{-}OH \rightarrow * + (H^+ + e^-)$$

where M-O and M-(vac) correspond to the lattice oxygen atom and lattice oxygen vacancy, respectively, which directly participates in the LOM. The adsorption Gibbs free energies were calculated as:

$$\Delta G_{OH*} = E(OH*) + 0.5*\mu(H_2) – E(*) – \mu(H_2O) + G_{corr,\ OH*}$$

$$\Delta G_{O*} = E(O*) + \mu(H_2) – E(*) – \mu(H_2O) + G_{corr,\ O*}$$

$$\Delta G_{M\text{-}OH} = E(M\text{-}OH) + 1.5*\mu(H_2) + \mu(O_2) – E(*) – 2*\mu(H_2O) + G_{corr,\ M\text{-}OH}$$

While we observed higher OER activity for $(RuIr)O_2$ compared to RuO_2 and IrO_2 for LOM, the calculated overpotentials are substantially higher than those of AEM (Fig. 8). This results is consistenet with previous theoretical[24,25] and experimental[26] findings, which suggest that the contribution of lattice oxygens to the overall OER activity is negligible for Ru-based and Ir-based catalysts.

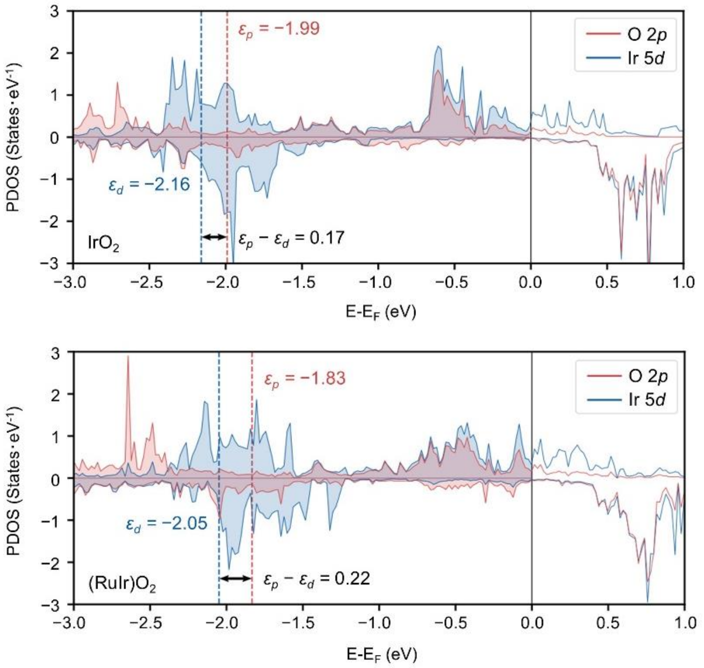

Supplementary Fig. 51 | PDOS and d-band center analysis for active Ir site and *p*-band center for adsorbate (O*). For $(RuIr)O_2$, the surface structure with the lowest energy shown as 32 in Supplementary Fig. 48 and the most stable active site shown as 32-1 in Supplementary Fig. 49a were considered.

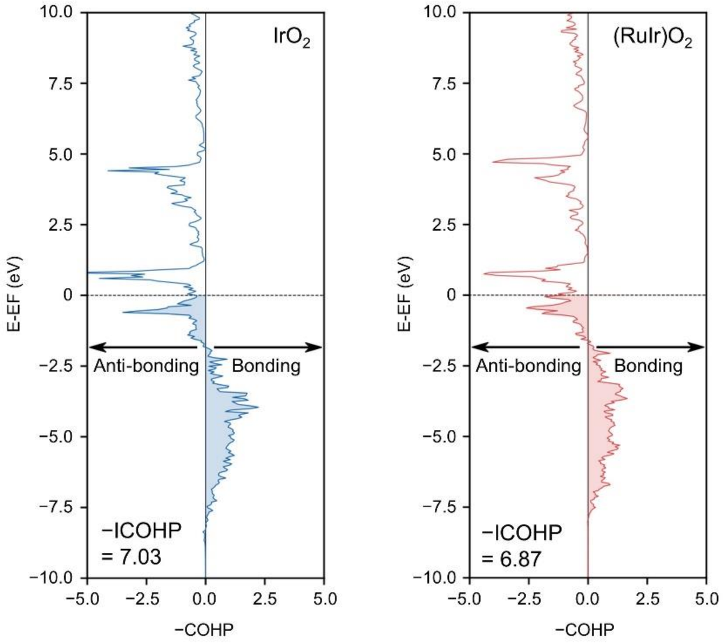

Supplementary Fig. 52 | The COHP analysis for O* adsorption on IrO_2 and the most stable $(RuIr)O_2$ surface.

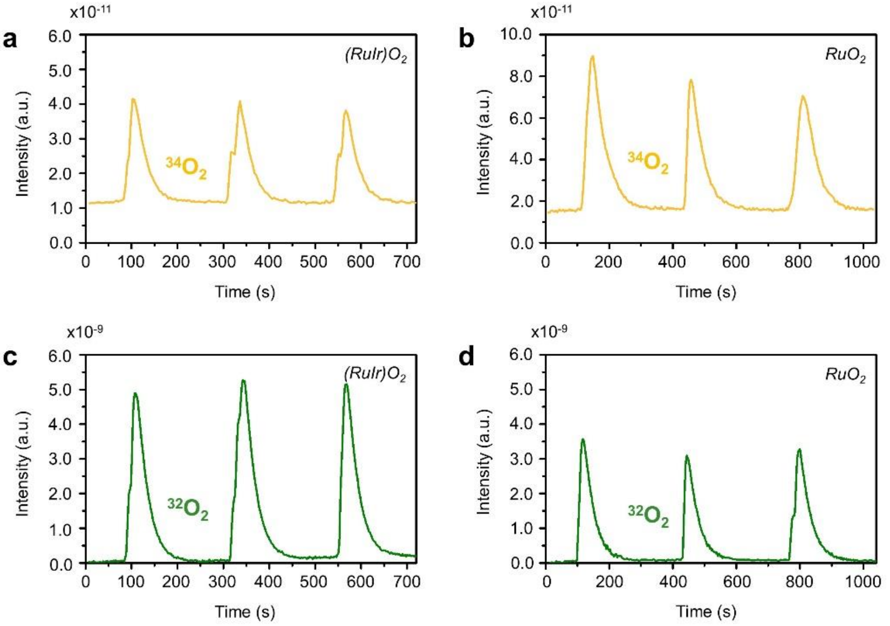

Supplementary Fig. 53 | DEMS analysis. DEMS measurement for $^{34}O_2$ ($^{16}O^{18}O$, yellow) and $^{32}O_2$ ($^{16}O^{16}O$, green) of **a**, **c** $(RuIr)O_2$/C and **b**, **d** homemade-RuO_2/C.

Supplementary Table 1 | Ni 2p XPS analysis. Binding energy (eV) used to fit the Ni 2p XPS spectra of Ni_3S_4, e-Ni_3S_4, (RuIr)O_2/C, and RuO_2@IrO_2/C.

Catalyst	Spin state	Oxidation state	Binding energy (eV)
Ni_3S_4	Ni $2p_{3/2}$	Ni^0	-
		Ni^{2+}	853.59
		Ni^{3+}	856.34
		satellite	861.61
e-Ni_3S_4	Ni $2p_{3/2}$	Ni^0	-
		Ni^{2+}	853.59
		Ni^{3+}	856.34
		satellite	861.61
(RuIr)O_2/C	Ni $2p_{3/2}$	Ni^0	851.72
		Ni^{2+}	853.59
		Ni^{3+}	856.34
		satellite	861.61
RuO_2@IrO_2/C	Ni $2p_{3/2}$	Ni^0	851.72
		Ni^{2+}	853.59
		Ni^{3+}	856.34
		satellite	861.61

Supplementary Table 2 | S 2p XPS analysis. Binding energy (eV) used to fit the S 2p XPS spectra of Ni_3S_4, and e-Ni_3S_4.

Catalyst	Spin state	Oxidation state	Binding energy (eV)
Ni_3S_4	S 2p	S $2p_{3/2}$	161.98
		S $2p_{1/2}$	163.35
		S_2^{2-}	164.60
		SO_4^{2-}	169.00
e-Ni_3S_4	S 2p	S $2p_{3/2}$	161.98
		S $2p_{1/2}$	163.35
		S_2^{2-}	164.60
		SO_4^{2-}	169.00

Supplementary Table 3 | Ru 3p XPS analysis. Binding energy (eV) used to fit the Ru 3p XPS spectra of $(RuIr)O_2/C$ and $RuO_2@IrO_2/C$ before and after OER operation.

Catalyst	Spin state	Oxidation state	Binding energy (eV)
$(RuIr)O_2/C$ before OER	Ru $3p_{3/2}$	Ru^0	461.45
		Ru^{3+}	464.10
		Ru^{4+}	462.45
$(RuIr)O_2/C$ after OER	Ru $3p_{3/2}$	Ru^0	461.25
		Ru^{3+}	463.90
		Ru^{4+}	462.25
		Ru^{6+}	464.80
$RuO_2@IrO_2/C$ before OER	Ru $3p_{3/2}$	Ru^0	461.45
		Ru^{3+}	464.10
		Ru^{4+}	462.45
$RuO_2@IrO_2/C$ after OER	Ru $3p_{3/2}$	Ru^0	461.45
		Ru^{3+}	464.10
		Ru^{4+}	462.45
		Ru^{6+}	465.00

Supplementary Table 4 | Ir 4f XPS analysis. Binding energy (eV) used to fit the Ir 4f XPS spectra of $(RuIr)O_2/C$ and $RuO_2@IrO_2/C$ before and after OER operation. The doublet separation between Ir $4f_{7/2}$ and Ir $4f_{5/2}$ was considered to be 0.25 eV.

Catalyst	Spin state	Oxidation state	Binding energy (eV)
$(RuIr)O_2/C$ before OER	If $4f_{5/2}$	Ir^0	61.05
		Ir^{3+}	62.20
		Ir^{4+}	61.70
$(RuIr)O_2/C$ after OER	If $4f_{5/2}$	Ir^0	61.30
		Ir^{3+}	62.45
		Ir^{4+}	61.95
		Ir^{5+}	63.45
$RuO_2@IrO_2/C$ before OER	If $4f_{5/2}$	Ir^0	61.05
		Ir^{3+}	62.20
		Ir^{4+}	61.70
$RuO_2@IrO_2/C$ after OER	If $4f_{5/2}$	Ir^0	61.05
		Ir^{3+}	62.20
		Ir^{4+}	61.70
		Ir^{5+}	63.20

Supplementary Table 5 | ICP-AES analysis results.

Catalyst	Ru (wt%)	Ir (wt%)	Ni (wt%)	S (wt%)
Ni_3S_4	-	-	51.70	48.30
e-NiS_4	-	1.06	51.01	47.93
$(RuIr)O_2/C$	45.74	25.02	6.08	2.75
$RuO_2@IrO_2/C$	45.27	26.18	5.84	2.17

Supplementary Table 6 | Comparison of OER performance of present and reported Ru/Ir-based catalysts in acidic media.

Catalyst	Catalyst loading (mg_{Ru+Ir}^{-2})	η [a] (mV)	MA (A mg_{Ru+Ir}^{-1}) @ V_{RHE}	TOF (s^{-1}) @ V_{RHE}	Stability [b] (hour)	Electrolyte	Ref.
$(RuIr)O_2$/C	0.050	174	6.87 @ 1.48	5.30 @ 1.48	360 [b-1]	0.1 M $HClO_4$	This study
RuO_2@IrO_2/C	0.050	225	1.75 @ 1.48	1.31 @ 1.48	40	0.1 M $HClO_4$	
Mn-RuIr	0.080	198	1.85 @ 1.48	N/A	180	0.1 M $HClO_4$	27
NiIrRuAl	0.030	237	1.63 @ 1.50	N/A	5 [b-2]	0.1 M $HClO_4$	28
Ru@IrO_x	0.050	281	0.65 @ 1.56	N/A	2	0.05 M H_2SO_4	29
Co-RuIr	0.050	235	N/A	N/A	25	0.1 M $HClO_4$	30
$Ir_{0.7}Ru_{0.3}O_2$	0.080	200	0.20 @ 1.62	N/A	10	0.1 M $HClO_4$	31
$RuIrO_x$	0.150	204	1.12 @ 1.53	0.47 @ 1.53	110	0.5 M H_2SO_4	32
$Ir_{0.17}Ru_{0.83}O_2$	0.275	177	1.10 @ 1.53	0.23 @ 1.53	20	0.5 M H_2SO_4	33
RuIr@CoNC	0.050	223	2.04 @ 1.50	N/A	40	0.5 M H_2SO_4	34
IrRu@Te	0.150	220	0.59 @ 1.48	N/A	20	0.5 M H_2SO_4	35
$Cr_{0.6}Ru_{0.4}O_2$	0.110	178	0.23 @ 1.50	0.15 @ 1.49	10	0.5 M H_2SO_4	36
$Mo_{0.15}$-RuO_2	0.230	147	0.66 @ 1.48	0.27 @ 1.48	20	0.5 M H_2SO_4	37
Mn-RuO_2	0.275	158	0.60 @ 1.50	0.39 @ 1.48	10	0.5 M H_2SO_4	38
$Re_{0.06}Ru_{0.94}O_2$	0.200	190	0.50 @ 1.50	0.17 @ 1.50	200	0.1 M $HClO_4$	39
$RuCoO_x$	0.043	200	2.28 @ 1.48	0.51 @ 1.48	100	0.1 M $HClO_4$	40
Ni-RuO_2	0.400	214	N/A	N/A	200	0.1 M $HClO_4$	41
RuIrAl	0.300	178	2.40 @ 1.53	0.04 @ 1.43	300 [b-1]	0.5 M H_2SO_4	25
PtCo-RuO_2/C	0.020	212	7.55 @ 1.48	1.98 @ 1.48	100	0.1 M $HClO_4$	42

a) overpotential (mV) at 10 mA cm^{-2} of current density

b) choronopotentiometry test at 10 mA cm^{-2} of current density

b-1) at 100 mA cm^{-2}

b-2) at 5 mA cm^{-2}

Supplementary Table 7 | O 1s XPS analysis. Binding energy (eV) used to fit the O 1s XPS spectra of $(RuIr)O_2/C$ and $RuO_2@IrO_2/C$ before and after OER operation.

Catalyst	Oxidation state	Binding energy (eV)
$(RuIr)O_2/C$ before and after OER	Lattice oxygen (M-O-M)	529.8
	Hydroxide (M-OH)	531.8
	Adsorbed H_2O (M-H_2O)	533.5
$RuO_2@IrO_2/C$ before and after OER	Lattice oxygen (M-O-M)	529.8
	Hydroxide (M-OH)	531.8
	Adsorbed H_2O (M-H_2O)	533.5

Supplementary Table 8 | Ru K-edge EXAFS analysis. Structural parameter of references (Ru foil and RuO_2) and catalysts from the EXAFS fitting for Ru K-edge. (S_0^2 = 0.80 was used to fit)

Sample	Scattering path	CN[a)]	R (Å)	σ^2 (10^{-3} Å^2)	ΔE_0 (eV)	R factor (%)
Ru foil	Ru-Ru_1	6	2.656±0.008	3.1±0.3	3.15±0.45	0.3
	Ru-Ru_2	6	2.702±0.008	2.9±0.3		
Ru(acac)$_3$	Ru-O	6	2.021±0.008	1.8±0.9	2.88±2.08	0.9
RuO_2	Ru-O	6	1.980±0.014	2.0±1.7	1.41±2.46	1.6
(RuIr)O_2 0.4 V	Ru-O	3.4±2.4	1.964±0.015	4.0±0.5	-2.50±7.46	0.3
	Ru-Ru	2.3±0.6	2.687±0.036	4.0±0.4		
	Ru-Ir	9.7±2.4	2.695±0.048	7.8±2.3		
(RuIr)O_2 1.2 V	Ru-O	5.0±1.3	1.989±0.023	6.5±3.0	3.98±3.62	3.0
(RuIr)O_2 1.45 V	Ru-O	3.5±2.9	2.025±0.026	2.0±0.7	8.83±3.91	0.9
(RuIr)O_2 1.6 V	Ru-O	4.0±1.5	1.963±0.022	2.0±0.3	1.82±6.11	2.2

a) coordination number

Supplementary Table 9 | Ir L_3-edge EXAFS analysis. Structural parameter of references (Ir black and IrO_2) and catalysts from the EXAFS fitting for Ir L_3-edge. (S_0^2 = 0.86 was used to fit)

Sample	Scattering path	CN[a)]	R (Å)	σ^2 (10^{-3} Å^2)	ΔE_0 (eV)	R factor (%)
Ir black	Ir-Ir	12	2.70±0.01	4.8±0.8	8.25±1.72	1.5
Ir(acac)$_3$	Ir-O	6	2.010±0.007	13.5±0.9	13.23±1.69	0.6
IrO_2	Ir-O	6	1.969±0.008	5.2±1.0	9.96±1.60	0.5
$(RuIr)O_2$ 0.4 V	Ir-O	1.1±0.9	1.967±0.034	10.3±0.5	5.95±4.57	0.4
	Ir-Ir	4.4±2.9	2.672±0.037	4.7±0.5		
	Ir-Ru	3.6±2.1	2.670±0.024	3.6±0.4		
$(RuIr)O_2$ 1.2 V	Ir-O	4.5±1.2	1.994±0.021	6.9±2.8	15.96±3.23	2.0
$(RuIr)O_2$ 1.45 V	Ir-O	4.7±1.4	1.987±0.023	4.3±3.0	15.98±4.13	2.0
$(RuIr)O_2$ 1.6 V	Ir-O	4.7±1.4	1.966±0.023	6.7±3.0	13.03±3.86	1.7

a) coordination number

1. Wan et al. Hierarchical porous Ni_3S_4 with enriched high-valence Ni sties as a robust electrocatalyst for efficient oxygen evolution reaction. *Adv. Funct. Mater*. **29**, 1900315 (2019).

2. Lv, J., Yan, C., Liu, W., Quan, B., Liang, X., Ji, G., Du, Y. Achieving better impedance matching by a sulfurization method through converting Ni into NiS/Ni_3S_4 composites. *J. Mater. Chem. C* **6**, 1822-1828 (2018).

3. Konkena et al. Metallic $NiPS_3$@NiOOH core-shell heterostructures as highly efficient and stable electrocatalyst for the oxygen evolution reaction. *ACS Catal.* **7**, 229-237 (2017).

4. Lian, K., Thorpe, S. J., Kirk, D. W. Electrochemical and surface characterization of electrocatalytically active amorphous Ni Co alloys. *Electrochim. Acta* **37**, 2029-2041 (1992).

5. Kresse, G., Furthmuller, J. Efficience of ab-initio total energy calculations for metals and semiconductors using a plane-wave basis set. *Comput. Mater. Sci.* **6**, 15-50 (1996).

6. Kresse, G., Hafner, J. Ab initio molecular dynamics for open-shell transition metals. *Phys. Rev. B* **48**, 13115 (1993).

7. Blochl, P. E. Projector augmented-wave method. *Phys. Rev. B* **50**, 17953 (1994)

8. Kresse, G., Joubert, D. From ultrasoft pseudopotentials to the projector augmented-wave method. *Phys. Rev. B* **59**, 1758 (1999).

9. Perdew, J. P., Burke, K., Ernzerhof, M. Generalized gradient approximation made simple, *Phys. Rev. Lett*. **77**, 3865 (1996).

10. Grimme, S. Semiempirical GGA-type density functional constructed with a long-range dispersion correction. *J. Comput. Chem.* **27**, 1787-1799 (2006).

11. Grimme, S., Antony, J., Ehrlich, S., Krieg, H. A consistent and accurate ab initio parametrization of density functional dispersion correction (DFT-D) for the 94 elements H-Pu. *J. Chem. Phys*. **132**, 154104 (2010).

12. Monkhorst, H. J., Pack, J. D. Special points for Brillouin-zone integrations. *Phys. Rev. B* **13**, 5188 (1976).

13. Larsen, A. H. et al. The atomic simulation environment – a python library for working with atoms. *J. Condens. Matter Phys*. **29**, 273002 (2017).

14. Norskov, J. K. et al. Origin of the overpotential for oxygen reduction at a fuel-cell cathode. *J. Phys. Chem. B* **108**, 17886-17892 (2004).

15. Bolzan, A. A., Fong, C., Kennedy, B. J., Howard, C. J. Structural studies of rutile-type metal dioxides. *Acta Crst.* **B53**, 373-380 (1997).

16. Ong, S. P. et al. Python materials genomics (pymatgen): a robust, open-source python library for materials analysis. *Comput. Mater. Sci*. **68**, 314-319 (2013).

17. Tang, W., Sanville, E., Henkelman, G. A grid-based bader analysis algorithm without lattice bias. *J. Condens. Matter Phys*. **21**, 084204 (2009).

18. Deringer, V. L., Tchougreeff, A. L., Dronskowski, R. Crystal orbital Hamilton population (COHP) analysis as projected form plane-wave basis sets. *J. Phys. Chem. A* **115**, 5461-5466 (2011).

19. Maintz, S., Deringer, V. L., Tchougreeff, A. L., Dronskowski, R. Lobster: a tool to extract chemical bonding from plane-wave based DFT. *J. Comput. Chem.* **37**, 1030-1035 (2016).

20. Lee, W., Kim, J., Kim, H., Back, S. Catalytic activity trends of pyrite transition metal dichalcogenides for oxygen reduction and evolution. *Phys. Chem. Chem. Phys.* **24**, 19911-19918 (2022).

21. Kim, J. et al. Structural insights into multi-metal spinel oxide nanoparticles for boosting oxygen reduction electrocatalysts. *Adv. Mater*. **34**, 2107868 (2022).

22. Hoover, W. G. Canonical dynamics: equilibrium phase-space distributions. *Phys. Rev. A* **31**, 1695 (1985).

23. Rao et al. Towards identifying the active sites on RuO_2 (110) in catalyzing oxygen evolution. *Energy Environ. Sci*. **10**, 2626-2637 (2017).

24. Zagalskaya, A., Alexandrov, V. Role of defects in the interplay between adsorbate evolving and lattice oxygen mechanisms of the oxygen evolution reaction in RuO_2 and IrO_2. *ACS Catal*. **10**, 3650-3657 (2020).

25. Lee, K. et al. Modulating the valence electronic structure using earth-abundant aluminum for high-performance acidic oxygen evolution reaction. *Chem* **9**, 3600-3612 (2023).

26. Scott, S. B., Sorensen, J. E., Rao, R. R., Moon, C., Kibsgaard, J., Shao-Horn, Y, Chorkendorff, I. The low overpotential regime of acidic water oxidation part II: trends in metal and oxygen stability numbers. *Energy Environ. Sci*. **15**, 1988-2001 (2022).

27. Joo, J. et al. Mn-dopant differentiating the Ru and Ir oxidation states in catalytic oxides toward durable oxygen evolution reaction in acidic electrolyte. *Small Methods* **6**, 2101236 (2022).

28. Liu, N. et al. Hierarchically porous nickel-iridium-ruthenium-aluminum alloys with tunable compositions and electrocatalytic activities towards the oxygen/hydrogen evolution reaction in acid electrolyte. *J. Mater. Chem. A* **8**, 6245-6255 (2020).

29. Shan, J. et al. Charge-redistribution-enhanced nanocrystalline Ru@IrO_x electrocatalysts for oxygen evolution in acidic media. *Chem.* **5**, 445-459 (2019).

30. Shan, J., Ling, T., Davey, K., Zheng, Y., Qiao, S. -Z. Transition-metal-doped RuIr bifunctional nanocrystals for overall water splitting in acidic environments. *Adv. Mater.* **31**, 1900510 (2019).

31. Zhang, J. et al. Iridium nanoparticles anchored on 3D graphite foam as a bifunctional electrocatalyst for excellent overall water splitting in acidic solution. *Nano Energy* **40**, 27-33 (2017).

32. He, J., Zhou, X., Xu, P., Sun, J. Regulating electron redistribution of intermetallic iridium oxide by incorporating Ru for efficient acidic water oxidation. *Adv. Energy Mater*. **11**, 2102883 (2021).

33. Chen, S. et al. An indium-induced-synthesis $In_{0.17}Ru_{0.83}O_2$ nanoribbon as highly active electrocatalyst for oxygen evolution in acidic media at high current densities above 400 mA cm^{-2}. *J. Mater. Chem. A* **10**, 3722-3731 (2022).

34. Xu, J. et al. Atomic-step enriched ruthenium-iridium nanocrystals anchored homogeneously on MOF-derived support for efficient and stable oxygen evolution in acidic and neutral media. *ACS catal.* **11**, 3402-3413 (2021).

35. Xu, J. et al. Strong electronic coupling between ultrafine iridium-ruthenium nanoclusters and conductive, acid-stable tellurium nanoparticle support for efficient and durable oxygen evolution in acidic and neutral media. *ACS Catal.* **10**, 3571-3579 (2020).

36. Lin, Y. et al. Chromium-ruthenium oxide solid solution electrocatalyst for highly efficient oxygen evolution reaction in acidic media. *Nat. Commun.* **10**, 162 (2019).

37. Zhang, Y. et al. Mo-doped mesoporous RuO_2 spheres as high-performance acidic oxygen evolution reaction electrocatalyst. *Small* **20**, 2305889 (2023).

38. Chen, S. et al. Mn-doped RuO_2 nanocrystals as highly active electrocatalysts for enhanced oxygen evolution in acidic media. *ACS Catal.* **10**, 1152-1160 (2020).

39. Jin, H. et al. Dynamic rhenium dopant boosts ruthenium oxide for durable oxygen evolution. *Nat. Commun.* **14**, 354 (2023).

40. Zhu, W. et al. Direct dioxygen radical coupling driven by octahedral ruthenium-oxygen-cobalt collaborative coordination for acidic oxygen evolution reaction. *J. Am. Chem. Soc.* **145**, 17995-18006 (2023).

41. Wu, Z. -Y. et al. Non-iridium-based electrocatalyst for durable acidic oxygen evolution reaction in proton exchange membrane water electrolysis. *Nat. Mater.* **22**, 100-108 (2023).

42. Jin, H. et al. Safeguarding the RuO2 phase against lattice oxygen oxidation during acidic water electrooxidation. *Energy Environ. Sci*. **15**, 1119-1130 (2022).
