# Supplementary material for: Atomic-level Ru-Ir mixing in rutile-type (RuIr)O2 for efficient and durable oxygen evolution catalysis
Source: Nat Commun. 2025 Jan 10;16:579. doi: 10.1038/s41467-025-55910-1 (PMC11723980; doi:10.1038/s41467-025-55910-1)
Supplement: Supplementary file 2 — Description of Additional Supplementary Files [file 41467_2025_55910_MOESM2_ESM.pdf]

File Name: Supplementary Data 1

Description of Contents:

This supplementary data contains CIF files that represent the structures used in the study. These files are organized into three folders based on the specific analysis conducted:

1. **M_on_Ni_3S_4_AIMD**:
   - This folder includes the initial and final structures of AIMD simulations for M (M = Ru, RuIr) on Ni_3S_4 surfaces.
   - The CIF files in this folder correspond to the structure shown in Fig. 2k in main text and Supplementary Fig. 7, 8
2. **Ir_location_bader**:
   - This folder contains CIF files of the structures used for Bader charge analysis based on the number and positions of Ir atoms.
   - The CIF files in this folder correspond to the structure shown in Supplementary Fig. 42.
3. **Ir_location_coverage**:
   - This folder includes CIF files of the structures based on the surface positions of Ir atoms and surface coverage (OH and O coverage).
   - The CIF files in this folder correspond to the structure shown in Supplementary Fig. 44.

The supplementary data is cited in the main text as **"Supplementary Data 1."**
